# SGLT2 inhibitor short-term efficacy and SYNTAX score association in coronary heart disease retinopathy: a propensity score matching study

**DOI:** 10.3389/fcvm.2025.1651251

**Published:** 2025-12-15

**Authors:** Qun Zhang, Zhenyan Wu, Xue Jiang, Yiran Wang, Yu Xin, Xinying Guo, Caixia Guo

**Affiliations:** Cardiovascular Center, Beijing Tongren Hospital, Capital Medical University, Beijing, China

**Keywords:** coronary heart disease, SYNTAX score, sodium-glucose cotransporter-2 inhibitors, coronary heart disease retinopathy, risk assessment

## Abstract

**Background:**

Type 2 diabetes (T2DM) and hypertension (HTN) are key risk factors for retinopathy and often coexist with coronary heart disease (CHD). While AI-based retinal imaging predicts CHD risk, links between CHD lesion complexity and retinopathy, and potential benefits of Sodium-Glucose Cotransporter-2 (SGLT2) inhibitors on retinopathy in CHD patients with T2DM, lack sufficient evidence.

**Methods:**

This case-control study (Jan 2024-Mar 2025) enrolled 642 subjects from Beijing Tongren Hospital (affiliated with Capital Medical University). Retinal imaging data were analyzed. Propensity score matching (PSM) created baseline and optimized datasets. Univariate/multivariate logistic regression assessed if CHD increases retinopathy risk in patients with T2DM, HTN, or both. Pearson correlation evaluated associations between CHD lesion complexity (SYNTAX score) and retinal parameters. Stratified analysis assessed SGLT2 inhibitor effects on retinopathy in hypertensive-diabetic subgroups with/without CHD. The analysis of SGLT2 inhibitor efficacy was based on a retrospective cohort study of drug utilization rather than a prospective randomized intervention.

**Results:**

Within the hypertensive-diabetic cohort, CHD patients (*n* = 97 SGLT2i treated vs. *n* = 69 controls) showed SGLT2 inhibitors reduced MRV(Venular)C risk (*P* < 0.001, OR = 0.584) but increased AVR1.5-2.0PD risk (*P* = 0.003). Post-PSM, CHD + T2DM + HTN patients had decreased MRAC vs. T2DM + HTN patients (*P* < 0.001, OR = 0.776). SYNTAX score positively correlated with retinal vein diameter and negatively with AVR (both *P* < 0.05).

**Conclusion:**

CHD elevates retinopathy risk in patients with T2DM, HTN, or both. Coronary lesion complexity correlates with retinal microvascular changes. SGLT2 inhibitors demonstrate a potential protective effect against some aspects of retinopathy development.

## Introduction

Globally, cardiovascular diseases (CVDs) remain the leading cause of morbidity and mortality (1). Although microvascular endothelial dysfunction has been proposed as an early trigger in CVD progression (2–4), and retinal microvascular changes may offer a non-invasive window into systemic vascular health (5–8), critical gaps persist in understanding the direct causal role of coronary heart disease (CHD) in retinopathy—particularly when disentangled from diabetes and hypertension. While diabetic retinopathy is well-established as a microvascular complication (9) of type 2 diabetes mellitus (T2DM) and is closely linked to coronary artery disease (CAD) (10, 11), it remains unclear whether CHD independently induces or exacerbates retinopathy, or merely represents part of systemic vascular aging.

Moreover, the absence of established treatment guidelines for retinopathy associated with CHD underscores the clinical relevance of this problem. Although the SYNTAX score (SS)—an angiographic measure of coronary lesion complexity—has been correlated with microvascular damage (12), including hypertensive and diabetic retinopathy (13), its utility in predicting or reflecting CHD-specific retinal pathology remains unvalidated. Simultaneously, SGLT2 inhibitors have emerged as cornerstone cardioprotective drugs with pleiotropic benefits (14). Beyond their metabolic and hemodynamic effects, their anti-inflammatory and anti-oxidant properties suggest potential for end-organ protection, including in the retina (15–19). However, evidence of their efficacy against CHD-associated retinopathy is lacking.

Therefore, this study aimed to resolve these interconnected clinical problems: First, to determine if CHD is an independent risk factor for retinopathy, after rigorously accounting for diabetes and hypertension using propensity score matching (PSM). Second, to evaluate whether the SYNTAX score can serve as a biomarker for retinopathy severity in this context. Finally, to explore the novel hypothesis that SGLT2 inhibitor therapy may mitigate the retinal complications of CHD.

## Materials and methods

### Study design and study population

**Figure 1:**
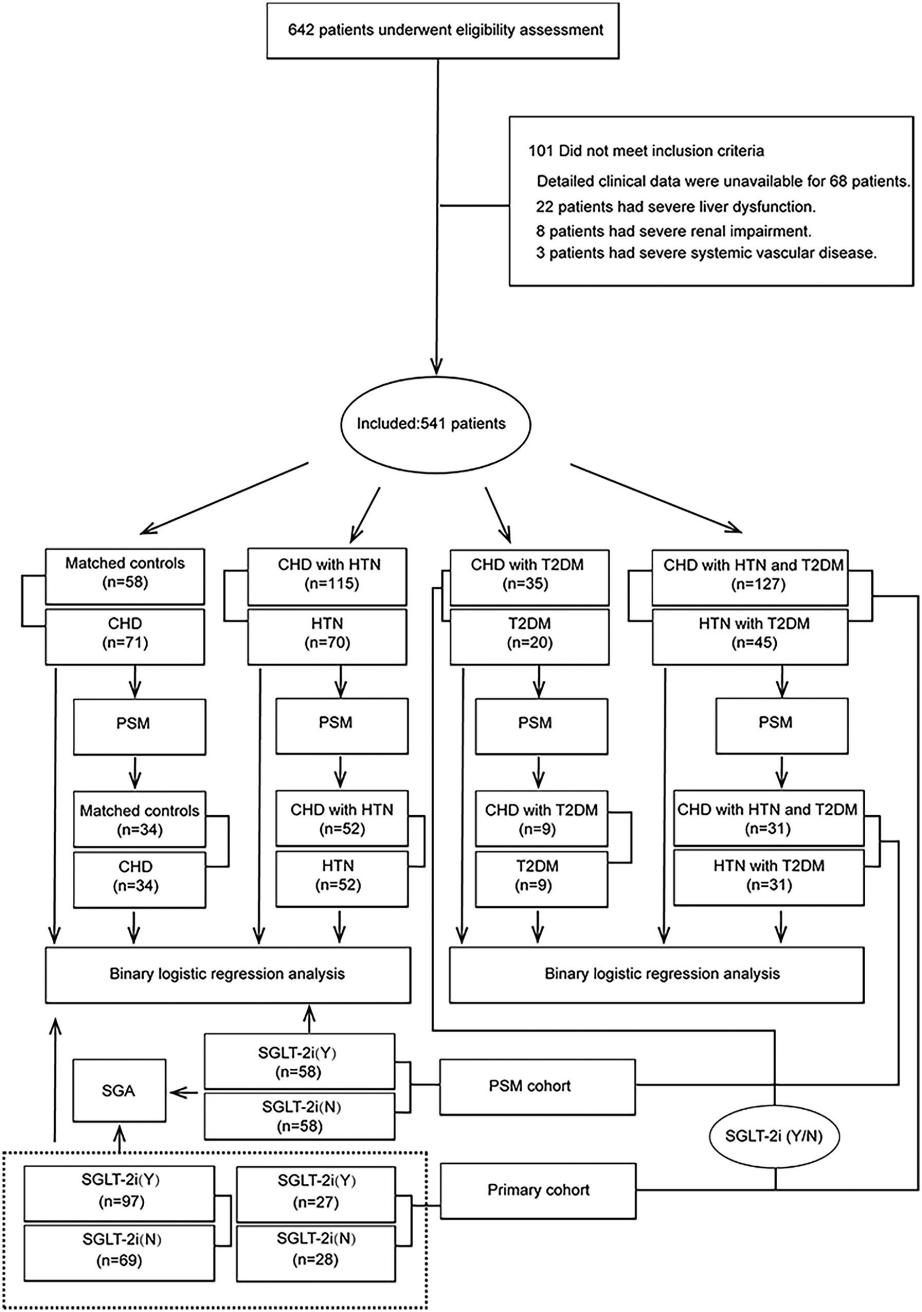
Study design and flow chart.

This case-control study gathered retinal imaging data from Beijing Tongren Hospital (affiliated with Capital Medical University) for the observation period spanning January 2024 to March 2025, with simultaneous acquisition of retinal imaging data ongoing from five tertiary centers—Beijing Tongren Hospital (affiliated with Capital Medical University), Xuanwu Hospital, Jishuitan Hospital, China-Japan Friendship Hospital, and the Department of Cardiology at Peking University First Hospital—and this multicenter data is slated for future use in validating and developing a model to identify patients at high risk of coronary heart disease. The recruitment comprised 642 subjects, incorporating 58 demographically comparable controls devoid of coronary pathology, hypertensive disorders, or type 2 diabetes. Eligibility for the coronary heart disease cohort mandated: (1) the mean age of the participants was 60.9 years; (2) availability of comprehensive ophthalmic imaging and coronary angiographic records. Confirming the diagnosis of coronary heart disease necessitated angiographic verification of ≥50% luminal narrowing in at least one major coronary artery; angiographic assessments were independently conducted by two cardiologists blinded to the study objectives, with discordant interpretations adjudicated through third-party arbitration to achieve diagnostic concordance. Exclusion parameters encompassed: (1) absence of confirmed coronary artery disease; (2) pharmacological hypersensitivity or treatment contraindications; (3) significant hepatorenal impairment; (4) hypotensive presentation (SBP <100 mmHg); (5) cognitive/psychiatric limitations hindering protocol adherence; (6) concurrent hyperkalemia or active malignancy. For granular analysis of CHD-associated retinal pathology, three experimental strata were constituted: hypertensive-CHD (*n* = 115), diabetic-CHD (*n* = 35), and hypertensive-diabetic-CHD (*n* = 127), each paired with respective comorbidity-specific control arms: hypertension-only (*n* = 70), T2DM-only (*n* = 20), and hypertensive-diabetic (*n* = 45). Assessment of the short-term efficacy of SGLT2 inhibitors on retinal manifestations led to stratification of the hypertensive diabetic CHD cohort and hypertensive diabetic control group into two arms (treatment group: *n* = 97; control group: *n* = 69) according to pre-admission SGLT2 inhibitor usage featuring a median three-month medication duration; implementation of a uniform propensity score matching protocol across all control groups established baseline and PSM-optimized analytical datasets, while Figure 1 details the complete methodological architecture.

### Selection of baseline characteristics

The analytical framework incorporated: Sociodemographic factors (age and gender); blood pressure parameters (three sequential random-zero sphygmomanometric measurements, analyzing the mean of latter two readings—operationalized hypertension as averaged resting systolic/diastolic pressures ≥140/90 mmHg from two assessments); antihypertensive treatment status (verified via prescribed medication use within 14 days pre-examination, self-declared diagnosis, or pharmaceutical documentation) (20); diabetes classification (ascertained through hypoglycemic agent prescriptions, clinician-confirmed self-reports, fasting glucose ≥126 mg/dL [7.0 mmol/L], non-fasting glucose ≥200 mg/dL [11.1 mmol/L], or HbA1c > 6.5% in untreated subjects without prior diabetes diagnosis, assigned zero-year disease duration) (13, 21); self-reported tobacco/alcohol consumption patterns; administered pharmacotherapies during hospitalization; continuous biochemical variables encompassing creatinine levels (Cr), fasting glycemia (FBS), Glycated Hemoglobin A1c (HbA1c), lipoprotein(a), triglyceride concentrations (TG), total cholesterol (TC), Low-Density Lipoprotein Cholesterol (LDL-C), High-Density Lipoprotein Cholesterol (HDL-C), complemented by retinal vascular morphometrics: angular branching characteristics, fractal complexity indices, mean vascular diameter, curvature intensity profiles, and arteriovenous tortuosity coefficients.

### Fundus image collection

Color fundus images were acquired using the Kestrel 3100 m system, with the imaging axis systematically positioned at the macular-optic disc midpoint to achieve ≥60° retinal visualization, and subsequent automated recognition and quantitative analysis of retinal vascular morphological features were performed using the proprietary EVisionAl fundus image analysis system (Yiwei Technology Co., Ltd., China) (22). Subjects were required to provide a minimum of one diagnostically valid fundus photograph meeting analytical and clinical interpretation requirements. Inclusion criteria mandated: (1) Precise anatomical registration of critical fundus structures with horizontal and vertical dimensions ≥50°, ensuring both foveal and optic disc margins maintained >2-disc-diameter distances from image peripheries; (2) Elimination of image-degrading shadows or glare artifacts, complemented by balanced exposure parameters rigorously excluding overexposed or underexposed photometric deviations.

Employing bio-inspired visual processing principles merged with artificial intelligence frameworks incorporating computational vision and deep neural networks, this approach achieves retinal vascular characterization through automated recognition and metric extraction. Algorithmic preprocessing stages encompassing noise reduction, contrast enhancement, and intensity normalization precede convolutional neural network architectures executing pixel-level vascular segmentation, facilitating geometric quantification through computational morphology to yield comprehensive vascular indices: mean branching angles, fractal dimensionality, generalized vessel diameter, curvature intensity profiles, arterial/venous tortuosity coefficients, vascular network density, arteriolar/venular caliper averages, and arteriole-to-venule diameter proportion metrics.

### Coronary angiography and SYNTAX score calculation

Cardiac catheterization procedures were conducted through standardized femoral/radial arterial approaches with 6-French diagnostic catheters. Two board-certified interventional cardiologists independently evaluated the coronary angiograms. Disease severity quantification incorporated critical left main coronary artery involvement (≥50% luminal narrowing) and the count of major epicardial vessels demonstrating ≥70% stenosis. A prospectively derived coronary stenosis scoring system (SS) systematically incorporated all clinically significant lesions (>50% diameter reduction in arteries >1.5 mm), employing the validated SS calculation methodology through an open-access digital platform (http://www.syntaxscore.com) (23). The investigation received formal ethical clearance from our institutional review board and strictly complied with international research ethics guidelines outlined in the Helsinki Declaration.

### Statistical analyses

A multiple-interpolation method was used to first process missing data for continuous and binary variables. Categorical data are presented as percentiles, whereas continuous variables with normal and non-normal distributions are presented as the mean ± standard deviation and median (interquartile range), respectively. The chi-squared test and Fisher's exact test were used to compare categorical data between the two groups, and the nonparametric Mann–Whitney *U* test and independent sample student's *t*-test were used to compare continuously distributed data between the groups. Binary logistic regression frameworks were developed to determine if coronary heart disease comorbidity potentiates retinopathy risk. Adjusted multivariate logistic models calculated odds ratios (OR) with 95% confidence intervals (95% CI) to establish directional associations between CHD comorbidity status and retinal vascular pathology likelihood compared to non-comorbid populations. Stratification analyses evaluated the ameliorative impact of SGLT2 inhibitors on microvascular ocular damage within hypertensive-diabetic subgroups, categorized by CHD diagnostic status. Propensity score matching protocols were administered across comparative cohorts to address confounding variables and reduce covariate distribution discrepancies, differentiating CHD-driven retinal pathology from focal vascular aging presentations. Within PSM-harmonized datasets, linear correlation assessments examined associations between SYNTAX score-defined coronary severity indices and quantitative retinal vascular dimensions. Individualized propensity scores were computed through covariate-adjusted logistic regression models integrating age and established risk parameters. A 1:1matching protocol with a greedy-matching algorithm was used for the match, and the caliper width of the logit standard deviation of the propensity score was 0.03.

## Results

### Baseline characteristics

**Figure 2:**
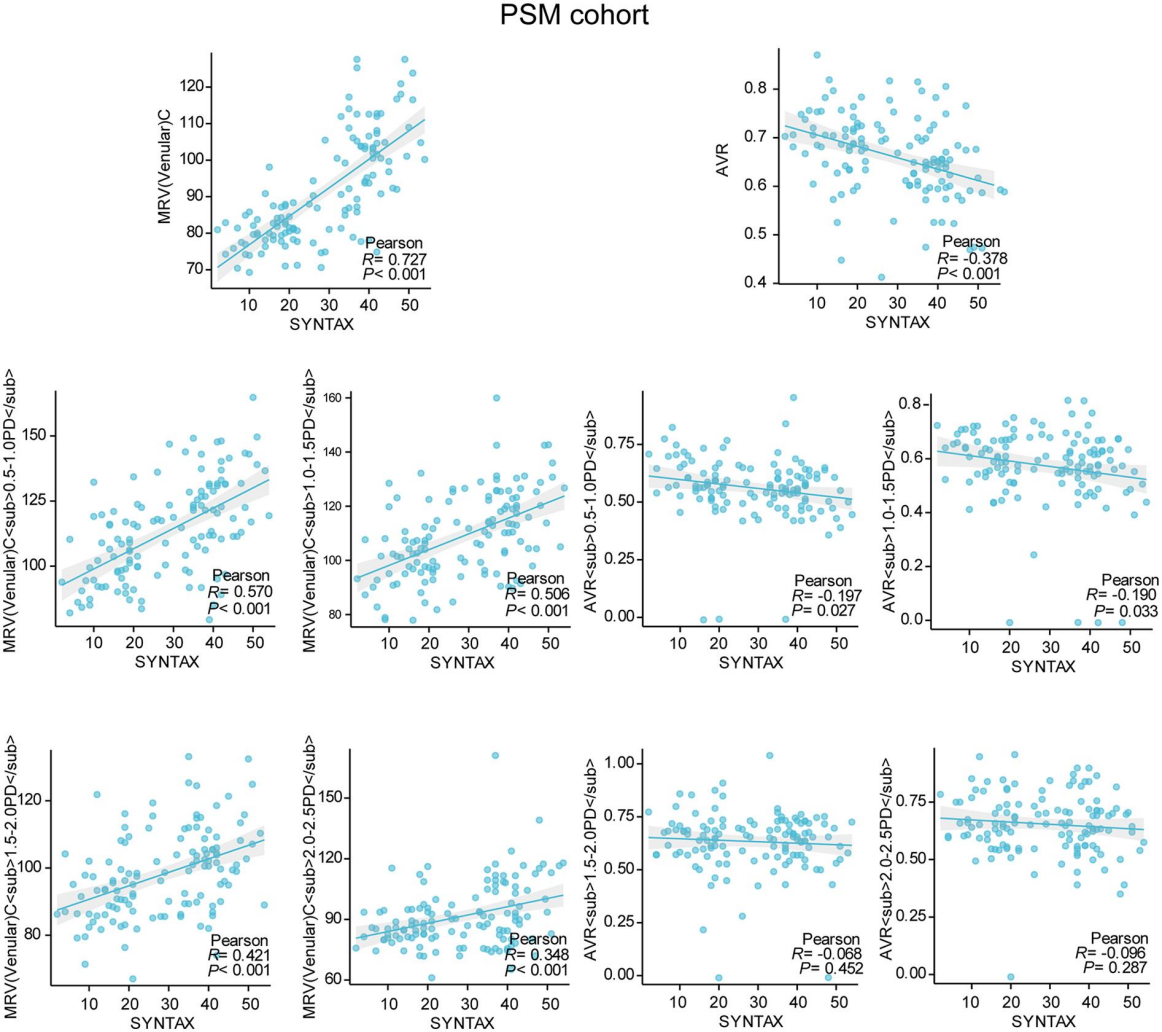
Violin plots of the primary cohort and the PSM cohort.

Of the initially planned 642 subjects, 101 were excluded for unmet inclusion criteria, yielding 541 participants in the primary cohort: 58 controls, 71 CAD, 70 HTN, 115 CAD + HTN, 20 T2DM, 35 CAD + T2DM, 45 HTN + T2DM, and 127 CAD + HTN + T2DM. The PSM cohort comprised 34 controls, 34 CAD, 52 HTN, 52 CAD + HTN, 9 T2DM, 9 CAD + T2DM, 31 HTN + T2DM, and 31 CAD + HTN + T2DM (study design: Figure 1). Ocular parameters differed significantly between groups in both cohorts (*P* < 0.05; Figure 2). While traditional risk factors (e.g., age) showed intergroup differences in the primary cohort (*P* < 0.05; Supplementary Tables S1–S4), these were eliminated after PSM for age and other factors (*P* > 0.05; same tables). Subsequent stratification by in-hospital SGLT2 inhibitor use (users vs. non-users) across diagnostic groups revealed 124 users and 97 non-users in the primary cohort, with subgroup distributions (T2DM vs. CAD + T2DM: 27 users/28 non-users; HTN + T2DM vs. CAD + HTN + T2DM: 97 users/69 non-users) showing persistent risk factor differences (*P* < 0.05; Supplementary Tables S5, S6). After PSM adjustment in the HTN + T2DM vs. CAD + HTN + T2DM subgroup (58 users/58 non-users), no significant differences remained (*P* > 0.05; Supplementary Table S5). Significantly reduced mean retinal venular diameter and arteriolar-to-venular ratio were observed in SGLT2 inhibitor users (*P* < 0.05; Figure 2).

### Univariate logistic regression analysis for retinal fundus lesion risk

To demonstrate CAD exacerbates retinal lesions in the primary cohort:

Control vs. CAD: CAD patients showed 1.370-fold higher mean retinal venular diameter (MRV) (*P* < 0.001, 95% CI: 1.218–1.541) with elevated MRV at C0.5–1.0PD (OR = 1.059, *P* < 0.001), C1.0–1.5PD (OR = 1.051, *P* = 0.004), C1.5–2.0PD (OR = 1.081, *P* < 0.001), and C2.0–2.5PD (OR = 1.060, *P* < 0.001), but reduced arteriolar-to-venular ratio (AVR) at 1.5–2.0PD (OR = 0.003, *P* = 0.001) and 2.0–2.5PD (OR = 0.002, *P* < 0.001) (Supplementary Table S7), indicating increased retinal risk.

HTN vs. CAD + HTN: CAD + HTN patients had 0.433-fold lower mean retinal arteriolar caliber (MRAC) (*P* < 0.001) and 0.001-fold lower AVR (*P* < 0.001), with decreased MRAC at Caliber0.5–1.0PD (OR = 0.860, *P* < 0.001), 1.0–1.5PD (OR = 0.818, *P* < 0.001), 1.5–2.0PD (OR = 0.836, *P* < 0.001), and 2.0–2.5PD (OR = 0.827, *P* < 0.001) (Supplementary Table S8), demonstrating compounded risk with CAD.

T2DM vs. CAD + T2DM: CAD + T2DM patients exhibited 1.094-fold higher MRV at C (OR = 1.094, *P* = 0.004) (Supplementary Table S9), confirming CAD aggravates diabetic retinopathy.

T2DM + HTN vs. CAD + HTN + T2DM: The CAD + HTN + T2DM group displayed lower MRAC (OR = 0.821, *P* < 0.001) and reduced MRAC at all caliber segments (*P* < 0.001), alongside decreased overall AVR (OR = 0.001, *P* = 0.004), AVR1.5–2.0PD (OR = 0.002, *P* = 0.001), and AVR2.0–2.5PD (OR = 0.019, *P* = 0.010) (Supplementary Table S10), establishing CAD intensifies retinal risk in T2DM + HTN patients.

To demonstrate that CAD independently exacerbates retinal fundus lesion risk beyond systemic vascular aging manifestations, PSM adjusted for age and traditional risk factors revealed:

In the control vs. CAD subgroup, CAD patients exhibited 1.320-fold higher MRV(Venular)C (*P* < 0.001, OR = 1.320, 95% CI:1.154–1.511) with increased MRVC at 0.5–1.0PD (OR = 1.070), 1.0–1.5PD (OR = 1.077), 1.5–2.0PD (OR = 1.100) and 2.0–2.5PD (OR = 1.059) (all *P* ≤ 0.008), alongside reduced AVR at 1.5–2.0PD (OR = 0.001) and 2.0–2.5PD (OR = 0.002) (both *P* ≤ 0.014; Supplementary Table S11), indicating elevated retinal lesion risk.

In HTN vs. CAD + HTN comparisons, CAD + HTN patients showed 0.220-fold lower MRAC (*P* = 0.001, 95% CI: 0.092–0.526) with decreased MRAC (Caliber) across all measured PD regions (OR = 0.764–0.858, all *P* < 0.001) and reduced AVR parameters (OR = 0.000–0.016, all *P* ≤ 0.015; Supplementary Table S12), confirming compounded retinal risk.

No significant risk increase occurred in T2DM patients with CAD (*P* > 0.05; Supplementary Table S13), potentially due to sample limitations. However, in T2DM + HTN vs. CAD + HTN + T2DM analyses, CAD + HTN + T2DM patients demonstrated 0.776-fold lower MRAC (*P* < 0.001, OR = 0.776, 95% CI: 0.677–0.889), reduced MRAC (Caliber) at all PD levels (OR = 0.882–0.924, all *P* ≤ 0.008) and lower AVR (*P* = 0.049, OR = 0.002; Supplementary Table S14), confirming CAD exacerbates retinal lesions in T2DM + HTN patients independently of systemic vascular aging.

### Multivariate logistic regression analysis for retinal fundus lesion risk

**Figure 4:**
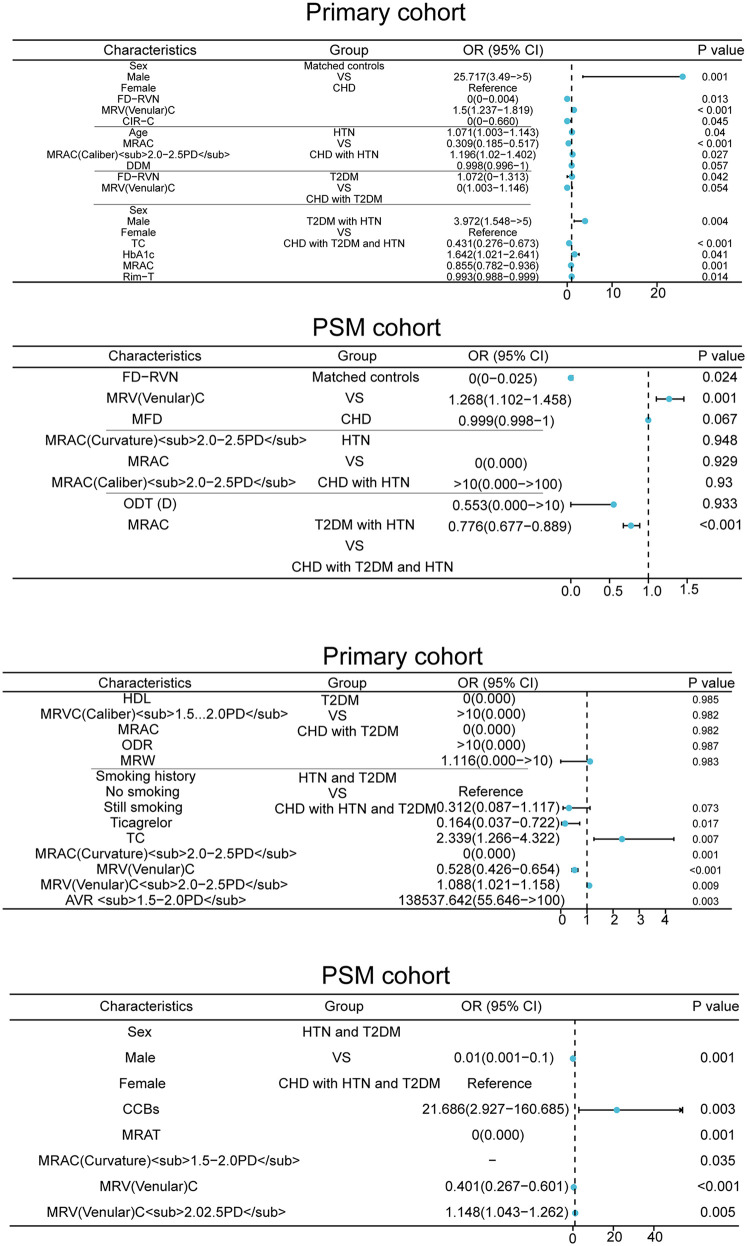
Multivariate logistic regression forest plot for both primary and PSM cohort.

The primary cohort employed multivariate logistic regression analysis using CAD presence as the dependent variable and demographic data (including age and sex), smoking/alcohol history, and fundus photography indicators as independent variables. CAD patients demonstrated a 1.5-fold elevation in MRV(Venular)C vs. controls (*P* < 0.001, 95% CI: 1.237–1.819) in the control vs. CAD subgroup, confirming increased retinal fundus lesion risk. Comparison of HTN vs. CAD + HTN groups revealed lower MRAC in CAD + HTN patients than HTN-only counterparts (*P* < 0.001, OR = 0.309, 95% CI: 0.185–0.517), indicating heightened retinal risk with CAD comorbidity. Within the T2DM + HTN vs. CAD + T2DM + HTN subgroup, CAD + T2DM + HTN patients exhibited both reduced MRAC (0.855-fold lower, *P* = 0.001, OR = 0.855, 95% CI: 0.782–0.936) and decreased Rim-T (*P* = 0.014, OR = 0.993, 95% CI: 0.988–0.999) relative to T2DM + HTN patients, establishing greater retinal lesion risk when CAD complicates T2DM + HTN (detailed in Supplementary Table S15 and Figure 4). The multivariate logistic regression model showed significant likelihood ratio test results (*P* = 0.040) with 1.00 goodness-of-fit, reflecting excellent model performance.

Analysis of the PSM cohort revealed that in the control vs. CAD case-control subgroup, CAD patients displayed elevated MRV(Venular)C levels—1.268-fold higher than controls (*P* = 0.001, 95% CI: 1.102–1.458). For the T2DM + HTN vs. CAD + T2DM + HTN comparison, CAD + T2DM + HTN patients manifested decreased MRAC relative to T2DM + HTN patients (*P* < 0.001, OR = 0.776, 95% CI: 0.677–0.889), with comprehensive data in Supplementary Table S16 and Figure 4. These outcomes demonstrate heightened retinal fundus lesion risk among CAD patients, establish CAD's role in exacerbating retinal lesion risk in T2DM + HTN patients, and confirm that this risk elevation is not attributable solely to localized manifestations of systemic vascular aging within the fundus vasculature.

### Analysis of the correlation between retinal fundus vessel diameter and coronary artery lesion complexity

**Figure 3:**
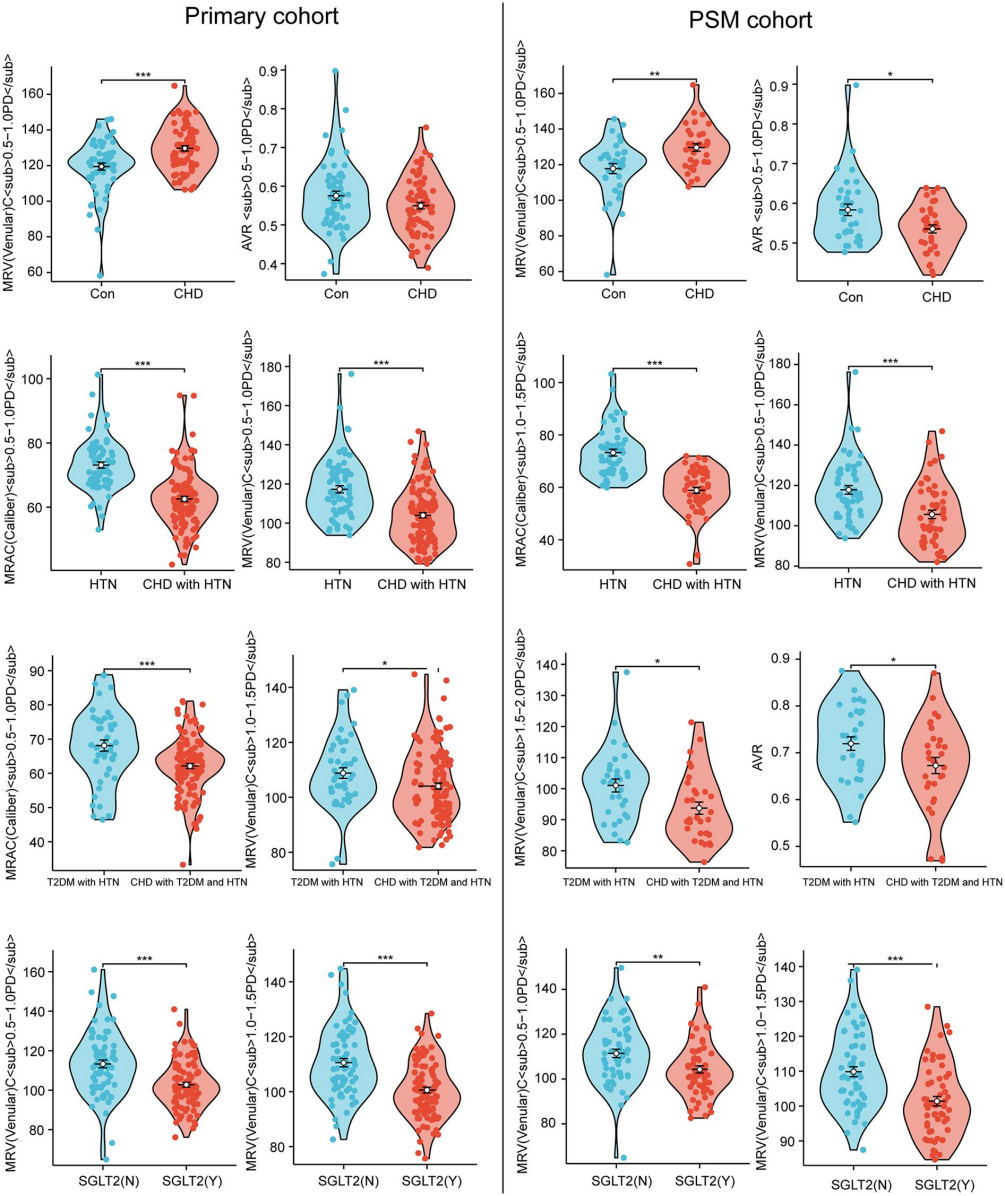
Evaluating retinal vessel diameter-coronary complexity correlation in the PSM cohort.

To evaluate whether coronary artery lesion complexity correlates with retinal fundus vessel diameter, correlation analysis was performed between SYNTAX scores and retinal fundus venular diameter as well as AVR, revealing a positive correlation between SYNTAX scores and retinal fundus venular diameter (*P* < 0.05) and a negative correlation between SYNTAX scores and AVR (*P* < 0.05), with detailed results shown in Figure 3.

### Univariate logistic regression of short-term SGLT2 inhibitor use

Stratification of T2DM and CAD + T2DM patients in the primary cohort by short-term SGLT2 inhibitor administration status revealed that SGLT2 inhibitor users had 0.917-fold lower MRV(Venular)C vs. non-users (*P* = 0.002, 95% CI: 0.867–0.969), exhibiting reduced MRV(Venular)C0.5–1.0PD (OR = 0.917, *P* < 0.001, 95% CI: 0.874–0.962) and MRV(Venular)C1.0–1.5PD (OR = 0.948, *P* = 0.034, 95% CI: 0.902–0.996) (Supplementary Table S17), signifying SGLT2 inhibitors' retinal lesion improvement effect. Similarly, categorization of T2DM + HTN and CAD + T2DM + HTN patients by short-term SGLT2 inhibitor use demonstrated 0.726-fold lower MRV(Venular)C in users than non-users (*P* < 0.001, 95% CI: 0.656–0.803) with concurrent MRV(Venular)C reductions at 0.5–1.0PD (OR = 0.947), 1.0–1.5PD (OR = 0.924), 1.5–2.0PD (OR = 0.901), and 2.0–2.5PD (OR = 0.942) (all *P* < 0.001; 95% CIs: 0.923–0.971, 0.894–0.955, 0.866–0.938, 0.916–0.970 respectively). Additionally, SGLT2 inhibitor users manifested 482,982.004-fold higher AVR (*P* < 0.001, 95% CI: 2,703.200–86,294,607.060) and elevated AVR at 0.5–1.0PD (OR = 128.142, *P* = 0.002, 95% CI: 6.315–2,600.104), 1.0–1.5PD (OR = 258.417, *P* = 0.001, 95% CI: 9.230–7,235.389), 1.5–2.0PD (OR = 716.475, *P* < 0.001, 95% CI: 21.887–23,453.502), and 2.0–2.5PD (OR = 51.324, *P* = 0.005, 95% CI: 3.205–821.754) vs. non-users (Supplementary Table S18), collectively confirming retinal fundus lesion improvement with SGLT2 inhibitor therapy.

Analysis of the PSM cohort comprising T2DM + HTN and CAD + T2DM + HTN patients categorized by short-term SGLT2 inhibitor use showed SGLT2 inhibitor recipients had 0.732-fold lower MRV(Venular)C than non-recipients (*P* < 0.001, 95% CI: 0.653–0.821), with reduced MRV(Venular)C at 0.5–1.0PD (OR = 0.959, *P* = 0.006), 1.0–1.5PD (OR = 0.926, *P* < 0.001), 1.5–2.0PD (OR = 0.921, *P* < 0.001), and 2.0–2.5PD (OR = 0.956, *P* = 0.005) (95% CIs: 0.930–0.988, 0.890–0.964, 0.881–0.963, 0.926–0.987 respectively). Additionally, SGLT2 inhibitor users exhibited 226,847.208-fold higher AVR (*P* < 0.001, 95% CI: 438.507->100) and elevated AVR at 0.5–1.0PD (OR = 61.896, *P* = 0.022), 1.0–1.5PD (OR = 75.003, *P* = 0.021), and 1.5–2.0PD (OR = 95.665, *P* = 0.023) (95% CIs: 1.803–2,125.410, 1.904–2,954.669, 1.860–4,919.231; Supplementary Table S19), confirming SGLT2 inhibitors' retinal fundus vasculopathy improvement effect.

### Subgroup analysis of patients receiving short-term SGLT2 inhibitor therapy

Subgroup analysis of the primary cohort evaluated short-term SGLT2 inhibitor use in patients with T2DM plus HTN and those with CHD plus T2DM and HTN to assess beneficial effects on retinopathy in both subgroups; results showed that in the CHD, T2DM, and HTN subgroup on SGLT2 inhibitors, MRV(Venular)C risk decreased (*P* < 0.001, OR = 0.584, 95% CI: 0.469–0.727) while AVR1.5–2.0PD risk increased (*P* = 0.003, OR = 1,785,742.893, 95% CI: 133.400–>100) (detailed in Supplementary Table S20), indicating maintained beneficial effects on retinopathy in this subgroup.Furthermore, the AMI subgroup demonstrated a 0.551-fold MRV(Venular)C reduction (*P* < 0.001, 95% CI: 0.429–0.708) and 11,058.482-fold AVR1.5–2.0PD increase (*P* = 0.024, 95% CI: 3.345–36,562,935.790) with short-term SGLT2 inhibitor use vs. non-users, demonstrating comparable retinal lesion improvement in AMI patients (Supplementary Table S21); however, the primary CHD + T2DM cohort's AMI subgroup analysis of short-term SGLT2 inhibitor use remained non-significant (*P* > 0.05) due to limited sample size (Supplementary Table S22).

### Multivariate logistic regression analysis of short-term SGLT2 inhibitor use

Multivariable logistic regression in the primary cohort of hospitalized patients with comorbid T2DM and HTN or CHD plus T2DM and HTN, using short-term SGLT2 inhibitor use (vs. non-use) as the dependent variable, showed that compared to non-users, SGLT2 inhibitor users had 0.528 times lower MRV(Venular)C risk (*P* < 0.001, 95% CI: 0.426–0.654) but 138,537.642 times higher AVR1.5–2.0PD risk (*P* = 0.003, 95% CI: 55.646–>100), detailed in Supplementary Table S23 and Figure 4.

Analyzing the PSM cohort of hospitalized patients with T2DM plus HTN or CHD plus T2DM and HTN based on short-term SGLT2 inhibitor use, SGLT2 inhibitor recipients vs. non-recipients showed 0.401 times lower MRV(Venular)C risk (*P* < 0.001, 95% CI: 0.267–0.601) (Supplementary Table S24 and Figure 4); multivariable logistic regression in both primary and PSM cohorts consistently demonstrated reduced retinopathy risk with short-term SGLT2 inhibitor use.

## Discussion

This study was conceived to address three pivotal and unresolved clinical questions: First, whether CHD independently contributes to retinopathy, distinct from the well-established effects of diabetes and hypertension. Second, whether the anatomical severity of coronary artery disease, quantified by the SS, correlates with the degree of retinal microvascular change. Third, whether SGLT2 inhibitors, with their documented cardioprotective effects, can mitigate this potential CHD-induced retinal damage. Our analyses provide compelling affirmative answers to these questions.

We definitively confirm that CHD is an independent risk factor for a specific pattern of retinal microvascular abnormalities characterized by arteriolar narrowing and venular enlargement. This pattern is a recognized biomarker of systemic microcirculatory impairment (24). Crucially, by applying PSM to rigorously control for confounders including age, diabetes, and hypertension, we demonstrated that these retinal vascular alterations are directly attributable to CHD itself, thereby decoupling them from the effects of systemic vascular aging. This finding refutes the notion that retinal changes in CHD patients are merely a localized manifestation of generalized aging and establishes CHD as an autonomous enhancer of retinal microvascular injury.

Furthermore, we established a significant quantitative link between coronary anatomy and retinal vasculature. In patients undergoing coronary angiography, our SYNTAX score-retinal vascular analyses revealed a direct correlation between higher SS and greater retinal venous diameter, complemented by a significant inverse correlation with the arterio-venous ratio. This suggests that the complexity and burden of epicardial atherosclerosis are directly mirrored in the retinal microcirculation. Mechanistically, this aligns with the pathophysiological postulate that epicardial obstructions can compromise microcirculatory perfusion through flow restriction or by impairing endothelial function, diminishing vasodilatory capacity and leading to venular dilation (24). Our findings extend the existing knowledge that retinal parameters are indicative of microvascular physiology (24) by demonstrating their specific correlation with the severity of macrovascular coronary disease.

Therapeutically, our most translatable finding is the associated protective effect of SGLT2 inhibitors. SGLT2 inhibitors are recognized for their cardioprotective and nephroprotective benefits, extending well beyond their glucose-lowering effects (25, 26). Recent reviews highlight that SGLT2 inhibitors confer retinoprotective effects by improving vascular remodeling and reducing sympathetic overactivation (27). Evaluating ocular outcomes in cohorts stratified by treatment status and validated through propensity-adjusted analyses, we observed that SGLT2 inhibitor exposure was associated with a marked reduction in the risk of retinal vascular deterioration. This substantiates their therapeutic potential in managing microvascular complications beyond their glycemic and cardiorenal benefits. While the cardioprotective effects of SGLT2 inhibitors—including reductions in cardiovascular death, myocardial infarction, and stroke in high-risk patients (28–30), as well as benefits on left ventricular remodeling and neurohormonal activation—are well-documented (31, 32), our data point to a novel application: protecting the retinal microvasculature from the specific insult of CHD. The anti-inflammatory, anti-fibrotic, and hemodynamic properties of these agents likely underpin this observed benefit, suggesting a pleiotropic mechanism of action that safeguards the microcirculation.

A central challenge in this field is dissecting the specific contribution of CHD from the powerful confounding effects of diabetes and hypertension. Our study was specifically designed to address this. We acknowledge that diabetic retinopathy, driven by sustained hyperglycemia-induced molecular cascades (13, 33), manifests in a high percentage of individuals after prolonged disease duration. Similarly, hypertension exerts profound and complex effects on the retinal microvasculature, with population-level studies (*n* = 55,000) validating blood pressure's bidirectional effects—constricting arteriolar and venular lumens while enhancing vascular tortuosity (7). Distinctive patterns exist, such as cumulative diastolic pressure inversely interacting with both vascular subtypes, contrasting systolic pressure's preferential arteriolar constriction (34–36).

It is against this established backdrop that our findings gain significance. By implementing stratified quadruple-group comparisons augmented by PSM, we were able to isolate the independent contribution of CHD. The retinal vascular alterations we observed in CHD patients—increased central retinal vein caliber, reduced central retinal artery dimensions, and diminished arteriole-venule diameter ratios—persisted robustly even after accounting for these potent confounders. This systematic approach allows us to definitively confirm CHD as an autonomous enhancer of retinal microvascular injury patterns.

The investigative findings reveal that CHD intensifies retinal microvascular abnormalities in high-risk populations, indicating that this subgroup requires prioritized implementation of preventive counseling and supervised pharmacological management. The fact that antihypertensive therapies have demonstrated reversibility of retinal arteriolar constriction (37) suggests that the retinal microvasculature is a dynamic and treatable target. Consequently, periodic fundoscopic tracking of vascular dimensions could serve as both an ancillary outcome measure in clinical trials and a tangible educational tool in practice, potentially bolstering patient engagement with lifestyle and therapeutic regimens.

The triglyceride-glucose index is a novel, accessible biomarker that independently predicts insulin resistance and cardiometabolic risk in clinical practice (38). While existing diagnostic paradigms rightly emphasize coronary CT and biochemical markers, non-invasive retinal assessments offer a unique opportunity for dynamic risk stratification and monitoring over time. Our prospective strategic objective is to leverage these ocular biomarkers, potentially integrated with AI machine learning, to construct cost-effective, preclinical detection frameworks for coronary pathology.

In the present study, the analysis of T2DM patients with concomitant CAD did not reveal a statistically significant increase in retinal risk (*P* > 0.05; Supplementary Table S13), a non-significant finding that warrants cautious interpretation as it may reflect the limited statistical power to detect modest effects in this specific subgroup rather than constituting definitive evidence for the absence of an association. Similarly, in the AMI subgroup analysis of the primary CHD + T2DM cohort, the effect of short-term SGLT2 inhibitor use did not reach statistical significance (*P* > 0.05; Supplementary Table S22), which is likely attributable to the substantially reduced sample size in this particular subgroup analysis that critically constrained the statistical power. A more plausible explanation for both non-significant findings lies in the constrained statistical power of our investigation imposed by the relatively limited cohort size; the small sample size diminished our capacity to detect small-to-moderate effect sizes, thereby increasing the risk of Type II errors. Consequently, the interpretation of these non-significant outcomes requires clarification through larger-scale, prospective studies specifically designed to elucidate these potential associations.

We must acknowledge that in this study, the *post hoc* power analysis conducted after propensity score matching revealed a statistical power (1-β error probability) of less than 75%, which was attributed to the limited sample size of the available dataset. We are actively pursuing multi-center data collection to validate these findings and to build robust clinical risk prediction models. A further major limitation of this research is that, even though our PSM successfully balanced the prevalence of diabetes and hypertension between groups, we fully acknowledge that this does not eliminate confounding from differences in disease severity and duration. Specifically, patients prescribed SGLT2 inhibitors may inherently have had more advanced or difficult-to-control diabetes, which is independently associated with both a higher SYNTAX score and the risk of retinopathy. Furthermore, the lack of detailed data on long-term glycemic control (e.g., HbA1c levels) and blood pressure management represents a potential source of unmeasured confounding, as these factors directly drive micro- and macrovascular complications and may have influenced treatment decisions. Therefore, the association we observed might partially reflect the channeling of SGLT2 inhibitors to a patient population with a greater underlying burden of disease, rather than solely a direct drug effect. Finally, it should be noted that our study did not investigate sex-specific associations, particularly regarding the relationship between MRAC/MRV(Venular)C and CHD risk, nor was gender integrated as a variable into the multivariate models or the propensity score matching protocol; this represents an additional limitation and will be a key objective in our planned future multi-center study to explore potential effect modifications and ensure more comprehensive risk stratification. The strengths of our study are its novel focus, the use of a quantitative angiographic score (SYNTAX), the application of PSM to strengthen causal inference (39–41), and the consistent results across analytical cohorts. By extending the known predictive value of retinal microvascular alterations for cardiac events (5, 25, 34, 39–48) our work provides a new, problem-oriented paradigm for understanding and treating the systemic microvascular consequences of coronary atherosclerosis.

## Data Availability

The datasets presented in this article are not readily available because the data derive from an unpublished multicenter cohort study; therefore, original data from this investigation are temporarily unavailable but will be provided in the future. Concurrently developing a clinical risk prediction model—integrating this multicenter data with AI machine learning—to identify high-risk coronary heart disease patients. Requests to access the datasets should be directed to ZhangQCMU@163.com.
