# Supplementary material for: SGLT2 inhibitor short-term efficacy and SYNTAX score association in coronary heart disease retinopathy: a propensity score matching study
Source: Front Cardiovasc Med. 2025 Dec 15;12:1651251. doi: 10.3389/fcvm.2025.1651251 (PMC12745156; doi:10.3389/fcvm.2025.1651251)
Supplement: Supplementary file 1 [file Table1.docx]

Supplemental Table 1. Demographic and clinical characteristics of matched control group and coronary heart disease (CHD) group participants.

|  | **Primary cohort** | | | |  | **PSM cohort** | | | |
| --- | --- | --- | --- | --- | --- | --- | --- | --- | --- |
|  | **CHD** | **Matched controls** | B | **P value** |  | **CHD** | **Matched controls** | B | **P value** |
| N | 71 | 58 |  |  |  | 34 | 34 |  |  |
| Age (years), mean(SD) | 59.200±11.776 | 57.55±10.827 | -0.818 | 0.415 |  | 56.650±12.487 | 56.970±11.328 | 0.112 | 0.911 |
| Sex |  |  | 20.736 | <0.0001 |  |  |  | - | 0.597 |
| Male, n(%) | 16(22.5) | 36(62.1) |  |  |  | 14(41.2) | 14(41.2) |  |  |
| Female, n(%) | 55(77.5) | 22(37.9) |  |  |  | 20(58.8) | 20(58.8) |  |  |
| Smoking history, n(%) |  |  | 13.211 | <0.0001 |  |  |  | - | 0.598 |
| No smoking | 30(42.3) | 43(74.1) |  |  |  | 21(61.8) | 21(61.8) |  |  |
| Still smoking | 41(57.7) | 15(25.9) |  |  |  | 13(38.2) | 13(38.2) |  |  |
| Drinking history, n(%) |  |  | 4.029 | 0.045 |  |  |  | 0.108 | 0.742 |
| No drinking | 54(76.1) | 52(89.7) |  |  |  | 28(82.4) | 29(85.3) |  |  |
| Still drinking | 17(23.9) | 6(10.3) |  |  |  | 6(17.6) | 5(14.7) |  |  |
| FBG (mmol/L), mean(SD) | 6.863±2.8817 | 6.300±2.183 | -1.228 | 0.415 |  | 6.171±1.690 | 6.350±2.099 | 0.388 | 0.699 |
| Cr (μmol/L), mean(SD) | 78.140±22.087 | 74.900±20.947 | -0.849 | 0.222 |  | 76.150±22.043 | 77.290±20.685 | 0.221 | 0.826 |
| Lp(a) (mmol/L), mean(SD) | 27.162±28.709 | 26.272±27.840 | -0.177 | 0.397 |  | 26.015±28.849 | 28.341±28.578 | 0.334 | 0.739 |
| TG (mmol/L), mean(SD) | 1.690±1.141 | 1.810±1.599 | 0.497 | 0.859 |  | 1.563±1.151 | 1.575±0.803 | 0.050 | 0.960 |
| TC (mmol/L), mean(SD) | 4.314±1.301 | 3.669±1.006 | -3.094 | 0.620 |  | 3.879±1.275 | 3.884±1.019 | 0.019 | 0.985 |
| LDL (mmol/L), mean(SD) | 2.539±1.116 | 1.987±0.894 | -3.051 | 0.002 |  | 2.252±1.135 | 2.195±0.911 | -0.229 | 0.820 |
| HDL (mmol/L), mean(SD) | 1.148±0.294 | 1.093±0.341 | -0.997 | 0.003 |  | 1.055±0.241 | 1.134±0.390 | 1.009 | 0.316 |
| HbA1c (%), mean(SD) | 6.565±1.008 | 6.529±1.111 | -0.190 | 0.321 |  | 6.718±1.162 | 6.547±1.082 | -0.627 | 0.533 |
| MBA, mean(SD) | 48.798±12.027 | 54.697±9.502 | 3.112 | 0.850 |  | 50.169±12.095 | 54.392±8.235 | 1.683 | 0.098 |
| FD-RVN (×10), mean(SD) | 1.434±0.042 | 1.452±0.053 | 2.121 | 0.002 |  | 1.437±0.039 | 1.466±0.050 | 2.728 | 0.008 |
| MRVC(Caliber) (μm), mean(SD) | 89.218±6.883 | 78.965±6.565 | -8.593 | 0.036 |  | 88.565±7.599 | 78.842±5.495 | -6.045 | <0.0001 |
| MRVC(Caliber)<sub>0.5–1.0PD</sub> | 97.000±10.100 | 92.663±12.362 | -2.193 | <0.0001 |  | 95.936±8.993 | 91.593±11.711 | -1.715 | 0.091 |
| MRVC(Caliber)<sub>1.0–1.5PD</sub> | 94.513±8.060 | 90.599±9.779 | -2.492 | 0.030 |  | 94.727±8.695 | 90.351±10.577 | -1.864 | 0.067 |
| MRVC(Caliber)<sub>1.5–2.0PD</sub> | 89.274±8.709 | 83.587±9.414 | -3.558 | 0.014 |  | 89.131±8.783 | 82.737±8.734 | -3.010 | 0.004 |
| MRVC(Caliber)<sub>2.0–2.5PD</sub> | 83.361±11.243 | 77.161±9.039 | -3.397 | 0.001 |  | 83.488±11.467 | 76.039±8.486 | -3.045 | 0.003 |
| MRV(Vessel)T (×10^4^) , mean(SD) | 0.0006±0.0002 | 0.0006±0.0001 | 2.003 | 0.001 |  | 0.0006±0.0002 | 0.0006±0.0001 | 1.264 | 0.211 |
| MRVκ<sub>0.5–1.0PD</sub> | 0.0006±0.0002 | 0.0006±0.0002 | 0.754 | 0.047 |  | 0.0006±0.0002 | 0.0007±0.0002 | 1.085 | 0.282 |
| MRVκ<sub>1.0–1.5PD</sub> | 0.0006±0.0003 | 0.0006±0.0002 | 0.522 | 0.452 |  | 0.0006±0.0003 | 0.0006±0.0002 | 0.461 | 0.646 |
| MRVκ<sub>1.5–2.0PD</sub> | 0.0006±0.0006 | 0.0007±0.0003 | 1.941 | 0.603 |  | 0.0006±0.0002 | 0.0007±0.0002 | 1.595 | 0.116 |
| MRVκ<sub>2.0–2.5PD</sub> | 0.0006±0.0003 | 0.0007±0.0002 | 0.740 | 0.055 |  | 0.0006±0.0003 | 0.0006±0.0002 | 0.625 | 0.534 |
| MRAT (×10^4^), mean(SD) | 0.0005±0.0002 | 0.0005±0.0001 | 0.565 | 0.460 |  | 0.0005±0.0002 | 0.0005±0.0001 | 0.363 | 0.718 |
| MRAC(Curvature)<sub>0.5-1.0PD</sub> | 0.0005±0.0002 | 0.0005±0.0003 | -0.431 | 0.573 |  | 0.0005±0.0002 | 0.0005±0.0003 | 0.150 | 0.881 |
| MRAC(Curvature)<sub>1.0-1.5PD</sub> | 0.0005±0.0003 | 0.0005±0.0002 | -0.486 | 0.667 |  | 0.0005±0.0003 | 0.0005±0.0002 | -0.089 | 0.929 |
| MRAC(Curvature)<sub>1.5-2.0PD</sub> | 0.0004±0.0002 | 0.0005±0.0002 | 0.998 | 0.628 |  | 0.0004±0.0002 | 0.0005±0.0003 | 1.435 | 0.156 |
| MRAC(Curvature)<sub>2.0-2.5PD</sub> | 0.0004±0.0002 | 0.0005±0.0002 | 0.882 | 0.320 |  | 0.0005±0.0003 | 0.0005±0.0002 | 0.088 | 0.930 |
| MRV(Venular)T (×10^4^), mean(SD) | 0.0007±0.0002 | 0.0007±0.0002 | 1.784 | 0.379 |  | 0.0007±0.0002 | 0.0007±0.0001 | 1.437 | 0.155 |
| MRVC(Curvature)<sub>0.5-1.0PD</sub> | 0.0007±0.0002 | 0.0007±0.0003 | 0.929 | 0.077 |  | 0.0007±0.0002 | 0.0007±0.0003 | 1.301 | 0.198 |
| MRVC(Curvature)<sub>1.0-1.5PD</sub> | 0.0007±0.0003 | 0.0007±0.0003 | 0.391 | 0.354 |  | 0.0007±0.0004 | 0.0007±0.0003 | 0.324 | 0.747 |
| MRVC(Curvature)<sub>1.5-2.0PD</sub> | 0.0007±0.0003 | 0.0008±0.0003 | 1.814 | 0.697 |  | 0.0007±0.0003 | 0.0008±0.0003 | 1.193 | 0.237 |
| MRVC(Curvature)<sub>2.0-2.5PD</sub> | 0.0008±0.0004 | 0.0008±0.0003 | 0.163 | 0.072 |  | 0.0007±0.0003 | 0.0007±0.0002 | 0.993 | 0.324 |
| VD (×10^2^), mean(SD) | 0.047±0.013 | 0.052±0.017 | 1.882 | 0.871 |  | 0.048±0.012 | 0.057±0.017 | 2.524 | 0.014 |
| MRAC (μm) , mean(SD) | 67.439±6.438 | 64.240±7.110 | -2.678 | 0.062 |  | 66.530±5.354 | 64.322±5.438 | -1.687 | 0.096 |
| MRAC(Caliber)<sub>0.5-1.0PD</sub> | 70.894±8.938 | 68.315±9.504 | -1.585 | 0.008 |  | 69.019±6.980 | 68.303±8.522 | -0.379 | 0.706 |
| MRAC(Caliber)<sub>1.0-1.5PD</sub> | 69.261±7.850 | 68.187±9.424 | -0.706 | 0.116 |  | 68.664±7.726 | 68.155±8.778 | -0.254 | 0.801 |
| MRAC(Caliber)<sub>1.5-2.0PD</sub> | 67.995±8.126 | 67.990±11.301 | -0.003 | 0.481 |  | 67.490±7.376 | 66.957±8.800 | -0.271 | 0.787 |
| MRAC(Caliber)<sub>2.0-2.5PD</sub> | 64.475±9.858 | 66.064±10.955 | 0.866 | 0.998 |  | 64.091±8.559 | 64.852±8.891 | 0.360 | 0.720 |
| MRV(Venular)C (μm), mean(SD) | 108.202±8.897 | 88.918±8.403 | -12.554 | 0.388 |  | 107.095±9.481 | 89.283±8.026 | -8.361 | <0.0001 |
| MRV(Venular)C<sub>0.5-1.0PD</sub> | 129.681±12.790 | 119.483±15.144 | -4.147 | <0.0001 |  | 129.621±12.262 | 117.799±16.482 | -3.356 | 0.001 |
| MRV(Venular)C<sub>1.0-1.5PD</sub> | 119.926±10.822 | 113.331±13.087 | -3.134 | <0.0001 |  | 119.933±9.624 | 112.626±10.671 | -2.965 | 0.004 |
| MRV(Venular)C<sub>1.5-2.0PD</sub> | 107.892±11.660 | 97.550±12.309 | -4.888 | 0.002 |  | 106.533±11.165 | 96.499±10.398 | -3.835 | <0.0001 |
| MRV(Venular)C<sub>2.0-2.5PD</sub> | 97.351±17.760 | 85.062±13.088 | -4.385 | <0.0001 |  | 98.840±19.800 | 85.364±13.640 | -3.268 | 0.002 |
| AVR , mean(SD) | 0.626±0.065 | 0.726±0.079 | 7.876 | <0.0001 |  | 0.624±0.054 | 0.725±0.079 | 6.141 | <0.0001 |
| AVR <sub>0.5-1.0PD</sub> | 0.550±0.072 | 0.575±0.090 | 1.804 | <0.0001 |  | 0.535±0.060 | 0.583±0.085 | 2.694 | 0.009 |
| AVR <sub>1.0-1.5PD</sub> | 0.581±0.078 | 0.606±0.097 | 1.621 | 0.074 |  | 0.575±0.071 | 0.605±0.063 | 1.834 | 0.071 |
| AVR <sub>1.5-2.0PD</sub> | 0.636±0.091 | 0.702±0.124 | 3.390 | 0.108 |  | 0.639±0.084 | 0.699±0.102 | 2.683 | 0.009 |
| AVR <sub>2.0-2.5PD</sub> | 0.675±0.127 | 0.774±0.123 | 4.476 | 0.001 |  | 0.665±0.124 | 0.765±0.133 | 3.199 | 0.002 |
| DA (μm^2^), mean(SD) | 3232952.986±664236.335 | 3052214.623±650372.610 | -1.552 | <0.0001 |  | 3294354.000±679637.524 | 3064634.256±715386.817 | -1.357 | 0.179 |
| HDD (μm), mean(SD) | 1938.717±203.984 | 1839.518±298.634 | -2.234 | 0.123 |  | 1958.409±196.924 | 1835.474±323.753 | -1.892 | 0.063 |
| VDD (μm), mean(SD) | 2117.730±239.709 | 2100.339±178.943 | -0.471 | 0.027 |  | 2144.836±259.435 | 2106.596±204.822 | -0.675 | 0.502 |
| ODR, mean(SD) | 0.852±0.057 | 0.806±0.118 | -2.726 | 0.638 |  | 0.837±0.059 | 0.808±0.117 | -1.246 | 0.217 |
| ODT (D), mean(SD) | -16.359±69.578 | -36.233±113.199 | -1.224 | 0.008 |  | -30.071±65.517 | -42.793±65.510 | -0.801 | 0.426 |
| MRW (μm), mean(SD) | 3517.388±1045.487 | 2454.726±1234.661 | -5.293 | 0.223 |  | 3377.811±929.209 | 2397.162±1095.248 | -3.981 | <0.0001 |
| DDM (μm), mean(SD) | 4788.075±528.443 | 4963.945±392.902 | 2.103 | <0.0001 |  | 4816.622±541.732 | 4968.881±394.795 | 1.324 | 0.190 |
| ODAR, mean(SD) | 1.134±0.073 | 1.229±0.286 | 2.474 | 0.037 |  | 1.152±0.081 | 1.216±0.292 | 1.227 | 0.224 |
| MFD (μm), mean(SD) | 2553.791±1457.866 | 4045.342±959.329 | 6.969 | 0.016 |  | 2704.738±1467.003 | 4154.361±803.423 | 5.054 | <0.0001 |
| OCA (μm^2^), mean(SD) | 849503.183±393789.737 | 852248.876±441827.819 | 0.037 | <0.0001 |  | 848246.765±373434.671 | 763844.871±316096.972 | -1.006 | 0.318 |
| CHD (μm), mean(SD) | 1013.057±225.861 | 967.849±260.178 | -1.056 | 0.970 |  | 1007.450±212.024 | 927.794±248.328 | -1.422 | 0.160 |
| CVD (μm), mean(SD) | 1016.239±234.191 | 1020.621±235.511 | 0.105 | 0.293 |  | 1027.789±232.371 | 984.075±224.301 | -0.789 | 0.433 |
| CIR-C, mean(SD) | 0.857±0.058 | 0.845±0.137 | -0.626 | 0.916 |  | 0.852±0.067 | 0.857±0.133 | 0.188 | 0.851 |
| CDR-A, mean(SD) | 0.256±0.076 | 0.264±0.096 | 0.568 | 0.532 |  | 0.251±0.070 | 0.240±0.080 | -0.594 | 0.554 |
| HCDR, mean(SD) | 0.520±0.084 | 0.515±0.119 | -0.274 | 0.571 |  | 0.512±0.081 | 0.497±0.103 | -0.668 | 0.506 |
| VCDR, mean(SD) | 0.477±0.074 | 0.481±0.094 | 0.280 | 0.784 |  | 0.477±0.069 | 0.464±0.086 | -0.682 | 0.498 |
| Rim-I (μm), mean(SD) | 529.695±108.734 | 508.118±136.894 | -0.998 | 0.780 |  | 537.469±101.370 | 525.154±138.033 | -0.419 | 0.676 |
| Rim-N (μm), mean(SD) | 485.764±102.442 | 456.867±141.416 | -1.344 | 0.320 |  | 476.691±106.730 | 472.861±126.072 | -0.135 | 0.893 |
| Rim-S (μm), mean(SD) | 577.911±102.380 | 539.997±131.602 | -1.840 | 0.181 |  | 577.847±97.390 | 574.799±109.810 | -0.121 | 0.904 |
| Rim-T (μm), mean(SD) | 442.877±119.835 | 423.126±140.432 | -0.862 | 0.068 |  | 471.508±109.071 | 442.919±135.064 | -0.960 | 0.340 |
| Max Rim-Cup D (μm), mean(SD) | 1685.016±211.625 | 1648.154±204.341 | -0.999 | 0.390 |  | 1697.919±206.700 | 1630.991±211.444 | -1.320 | 0.191 |
| Min Rim (μm), mean(SD) | 365.988±89.390 | 355.697±118.062 | -0.563 | 0.319 |  | 378.588±88.769 | 375.893±130.348 | -0.100 | 0.921 |

MBA, Mean Branching Angle; FD-RVN,Fractal Dimension of the Retinal Vascular Network; MRVC(Caliber) , Mean Retinal Vessel Caliber; MRVC(Caliber)<sub>0.5-1.0PD</sub>, Mean Retinal Vessel Caliber within 0.5-1.0 PD Zone; MRV(Vessel)T, Mean Retinal Vessel Tortuosity; MRVκ<sub>0.5-1.0PD</sub>, Mean Retinal Vessel Curvature within 0.5-1.0 PD Zone; MRAT, Mean Retinal Arteriolar Tortuosity; MRAC(Curvature)<sub>0.5-1.0PD</sub>, Mean Retinal Arteriolar Curvature within 0.5-1.0 PD Zone; MRV(Venular)T, Mean Retinal Venular Tortuosity; MRVC(Curvature)<sub>0.5-1.0PD</sub> ,Mean Retinal Venular Curvature within 0.5-1.0 PD Zone; VD, Vessel Density; MRAC, Mean Retinal Arteriolar Caliber ; MRAC(Caliber)<sub>0.5-1.0PD</sub> , Mean Retinal Arteriolar Caliber within 0.5-1.0 PD Zone; MRV(Venular)C, Mean Retinal Venular Caliber ; MRV(Venular)C<sub>0.5-1.0PD</sub>, Mean Retinal Venular Caliber within 0.5-1.0 PD Zone; AVR, Arteriolar-to-Venular Ratio; DA, Optic Disc Area; HDD, Horizontal Diameter of the Optic Disc; VDD, Vertical Optic Disc Diameter; ODR, Optic Disc Roundness; ODT (D), Optic Disc Tilt; MRW, Minimum Rim Width; DDM, Disc-Macula Distance; ODAR, Optic Disc Axial Ratio; MFD, Macular Fovea Distance; OCA, Optic Cup Area; CHD, Cup Horizontal Diameter; CVD, Cup Vertical Diameter; CIR-C, Cup Circularity; CDR-A, Cup-to-Disc Area Ratio; HCDR, Horizontal Cup-to-Disc Ratio; VCDR, Vertical Cup-to-Disc Ratio; Rim-I, Inferior Neuroretinal Rim Width; Rim-N, Nasal Neuroretinal Rim Width; Rim-S, Superior Neuroretinal Rim Width; Rim-T, Temporal Neuroretinal Rim Width; Max Rim-Cup D, Maximum Rim-to-Cup Distance; Min Rim, Minimum Neuroretinal Rim Width; Data are mean±SD or n (%). Continuous and categorical variables were compared using the independent samples t-test/Mann-Whitney U test and Chi-squared/Fisher's exact test, respectively. P < 0.05 was considered significant.

Supplemental Table 2. Demographic and clinical characteristics of HTN group and CHD with HTN group participants.

|  | **Primary cohort** | | | |  | **PSM cohort** | | | |
| --- | --- | --- | --- | --- | --- | --- | --- | --- | --- |
|  | **CHD with HTN** | **HTN** | B | **P value** |  | **CHD with HTN** | **HTN** | B | **P value** |
| N | 115 | 70 |  |  |  | 52 | 52 |  |  |
| Age (years), mean(SD) | 62.800±10.662 | 60.170±11.925 | -1.554 | 0.122 |  | 63.710±10.959 | 60.920±11.212 | -1.283 | 0.203 |
| Sex |  |  | 7.770 | 0.005 |  |  |  | 0.158 | 0.691 |
| Male, n(%) | 34(29.6) | 35(50) |  |  |  | 23(44.2) | 21(40.4) |  |  |
| Female, n(%) | 81(70.4) | 35(50) |  |  |  | 29(55.8) | 31(59.6) |  |  |
| Smoking history, n(%) |  |  | 2.353 | 0.125 |  |  |  | 0.156 | 0.693 |
| No smoking | 59(51.3) | 44(62.9) |  |  |  | 28(53.8) | 30(57.7) |  |  |
| Still smoking | 56(48.7) | 26(37.1) |  |  |  | 24(46.2) | 22(42.3) |  |  |
| Drinking history, n(%) |  |  | 1.984 | 0.159 |  |  |  | 0.060 | 0.807 |
| No drinking | 86(73.9) | 58(82.9) |  |  |  | 42(80.8) | 41(7.8) |  |  |
| Still drinking | 30(26.1) | 12(17.1) |  |  |  | 10(19.2) | 11(21.2) |  |  |
| FBG (mmol/L), mean(SD) | 5.555±0.915 | 5.531±0.918 | -0.170 | 0.865 |  | 5.575±0.956 | 5.562±0.813 | -0.077 | 0.938 |
| Cr (μmol/L), mean(SD) | 85.237±77.478 | 71.729±14.329 | -1.442 | 0.151 |  | 71.681±17.671 | 73.923±14.403 | 0.709 | 0.480 |
| Lp(a) (mmol/L), mean(SD) | 25.623±27.807 | 19.439±22.187 | -1.667 | 0.097 |  | 21.590±25.322 | 21.225±24.886 | -0.074 | 0.941 |
| TG (mmol/L), mean(SD) | 1.575±0.868 | 1.449±1.100 | -0.865 | 0.388 |  | 1.489±0.856 | 1.556±1.195 | 0.327 | 0.744 |
| TC (mmol/L), mean(SD) | 3.893±1.303 | 4.461±.0.872 | 3.546 | <0.0001 |  | 4.403±1.371 | 4.374±0.933 | -0.126 | 0.900 |
| LDL (mmol/L), mean(SD) | 2.159±1.098 | 2.569±0.766 | 2.983 | 0.003 |  | 2.518±1.178 | 2.524±0.806 | 0.030 | 0.976 |
| HDL (mmol/L), mean(SD) | 1.182±0.357 | 1.349±0.361 | 3.072 | 0.002 |  | 1.322±0.420 | 1.288±0.327 | -0.464 | 0.644 |
| HbA1c (%), mean(SD) | 6.023±0.505 | 5.933±0.526 | -1.165 | 0.246 |  | 5.985±.474 | 5.975±0.525 | -0.098 | 0.922 |
| MBA, mean(SD) | 61.555±10.822 | 56.173±12.050 | -3.141 | 0.002 |  | 59.583±11.988 | 55.395±11.735 | -1.800 | 0.075 |
| FD-RVN (×10), mean(SD) | 1.478±0.067 | 1.464±0.055 | -1.450 | 0.149 |  | 1.464±0.079 | 1.459±0.053 | -0.336 | 0.738 |
| MRVC(Caliber) (μm), mean(SD) | 70.549±6.326 | 82.446±8.781 | 9.882 | <0.0001 |  | 71.881±6.531 | 82.753±9.348 | 6.875 | <0.0001 |
| MRVC(Caliber)<sub>0.5–1.0PD</sub> | 86.127±11.561 | 96.797±8.770 | 6.643 | <0.0001 |  | 86.801±13.559 | 97.225±9.155 | 4.595 | <0.0001 |
| MRVC(Caliber)<sub>1.0–1.5PD</sub> | 85.017±11.208 | 95.424±10.313 | 6.310 | <0.0001 |  | 85.893±13.299 | 96.130±10.900 | 4.293 | <0.0001 |
| MRVC(Caliber)<sub>1.5–2.0PD</sub> | 79.996±8.248 | 89.539±10.149 | 6.985 | <0.0001 |  | 79.936±9.410 | 89.935±10.809 | 5.031 | <0.0001 |
| MRVC(Caliber)<sub>2.0–2.5PD</sub> | 75.121±7.328 | 84.148±11.672 | 5.812 | <0.0001 |  | 75.803±7.684 | 83.953±12.980 | 3.896 | <0.0001 |
| MRV(Vessel)T (×10^4^) , mean(SD) | 0.0008±0.0002 | 0.0007±0.0002 | -2.732 | 0.007 |  | 0.0008±0.0002 | 0.0007±0.0002 | -2.323 | 0.022 |
| MRVκ<sub>0.5–1.0PD</sub> | 0.0008±0.0002 | 0.0007±0.0002 | -2.512 | 0.013 |  | 0.0008±0.0002 | 0.0007±0.0002 | -2.901 | 0.005 |
| MRVκ<sub>1.0–1.5PD</sub> | 0.0007±0.0002 | 0.0007±0.0002 | -1.399 | 0.163 |  | 0.0007±0.0002 | 0.0007±0.0002 | -1.223 | 0.224 |
| MRVκ<sub>1.5–2.0PD</sub> | 0.0008±0.0003 | 0.0007±0.0002 | -2.703 | 0.008 |  | 0.0007±0.0002 | 0.0006±0.0002 | -2.020 | 0.046 |
| MRVκ<sub>2.0–2.5PD</sub> | 0.0008±0.0002 | 0.0008±0.0002 | -0.848 | 0.397 |  | 0.0008±0.0002 | 0.0007±0.0002 | -0.965 | 0.337 |
| MRAT (×10^4^), mean(SD) | 0.0006±0.0002 | 0.0006±0.0002 | -1.586 | 0.114 |  | 0.0006±0.0002 | 0.0006±0.0002 | -2.189 | 0.031 |
| MRAC(Curvature)<sub>0.5-1.0PD</sub> | 0.0006±0.0003 | 0.0006±0.0003 | -1.326 | 0.187 |  | 0.0006±0.0004 | 0.0005±0.0002 | -1.780 | 0.079 |
| MRAC(Curvature)<sub>1.0-1.5PD</sub> | 0.0006±0.0002 | 0.0006±0.0003 | -0.413 | 0.680 |  | 0.0006±0.0003 | 0.0006±0.0003 | 0.041 | 0.968 |
| MRAC(Curvature)<sub>1.5-2.0PD</sub> | 0.0006±0.0002 | 0.0005±0.0002 | -2.637 | 0.009 |  | 0.0006±0.0002 | 0.0005±0.0002 | -2.991 | 0.003 |
| MRAC(Curvature)<sub>2.0-2.5PD</sub> | 0.0006±0.0002 | 0.0006±0.0003 | 0.756 | 0.451 |  | 0.0006±0.0002 | 0.0006±0.0002 | 0.260 | 0.795 |
| MRV(Venular)T (×10^4^), mean(SD) | 0.0009±0.0002 | 0.0008±0.0002 | -2.580 | 0.011 |  | 0.0008±0.0002 | 0.0008±0.0002 | -1.569 | 0.120 |
| MRVC(Curvature)<sub>0.5-1.0PD</sub> | 0.0008±0.0003 | 0.0007±0.0002 | -2.373 | 0.019 |  | 0.0008±0.0003 | 0.0007±0.0002 | -1.851 | 0.067 |
| MRVC(Curvature)<sub>1.0-1.5PD</sub> | 0.0008±0.0003 | 0.0007±0.0002 | -1.292 | 0.198 |  | 0.0008±0.0003 | 0.0007±0.0003 | -1.194 | 0.235 |
| MRVC(Curvature)<sub>1.5-2.0PD</sub> | 0.0009±0.0003 | 0.0008±0.0003 | -1.753 | 0.081 |  | 0.0008±0.0003 | 0.0008±0.0003 | -0.668 | 0.506 |
| MRVC(Curvature)<sub>2.0-2.5PD</sub> | 0.0009±0.0003 | 0.0008±0.0003 | -1.510 | 0.133 |  | 0.0009±0.0003 | 0.0008±0.0003 | -1.169 | 0.245 |
| VD (×10^2^), mean(SD) | 0.061±0.019 | 0.059±0.017 | -0.561 | 0.575 |  | 0.057±0.021 | 0.058±0.017 | 0.097 | 0.923 |
| MRAC (μm) , mean(SD) | 55.718±4.739 | 67.650±6.160 | 13.896 | <0.0001 |  | 55.426±5.298 | 67.423±6.691 | 10.135 | <0.0001 |
| MRAC(Caliber)<sub>0.5-1.0PD</sub> | 62.546±8.886 | 73.116±8.126 | 8.100 | <0.0001 |  | 60.266±7.626 | 73.077±8.840 | 7.913 | <0.0001 |
| MRAC(Caliber)<sub>1.0-1.5PD</sub> | 60.545±8.242 | 73.237±9.155 | 9.738 | <0.0001 |  | 58.861±8.427 | 73.246±9.418 | 8.208 | <0.0001 |
| MRAC(Caliber)<sub>1.5-2.0PD</sub> | 59.666±8.622 | 72.119±10.260 | 8.858 | <0.0001 |  | 58.765±10.169 | 72.778±11.027 | 6.736 | <0.0001 |
| MRAC(Caliber)<sub>2.0-2.5PD</sub> | 57.236±7.199 | 69.161±11.716 | 7.679 | <0.0001 |  | 56.962±7.75 | 68.833±13.152 | 5.607 | <0.0001 |
| MRV(Venular)C (μm), mean(SD) | 81.633±7.765 | 93.224±12.607 | 6.933 | <0.0001 |  | 83.510±7.526 | 93.467±13.079 | 4.759 | <0.0001 |
| MRV(Venular)C<sub>0.5-1.0PD</sub> | 103.976±14.326 | 117.266±15.579 | 5.919 | <0.0001 |  | 105.632±15.165 | 117.803±15.310 | 4.073 | <0.0001 |
| MRV(Venular)C<sub>1.0-1.5PD</sub> | 102.203±13.170 | 113.374±14.981 | 5.308 | <0.0001 |  | 104.539±15.525 | 114.861±15.720 | 3.369 | 0.001 |
| MRV(Venular)C<sub>1.5-2.0PD</sub> | 93.807±11.011 | 101.942±9.997 | 5.043 | <0.0001 |  | 94.151±12.057 | 102.382±10.971 | 3.641 | <0.0001 |
| MRV(Venular)C<sub>2.0-2.5PD</sub> | 87.358±10.863 | 94.593±13.350 | 4.023 | <0.0001 |  | 88.566±11.854 | 94.563±14.620 | 2.298 | 0.024 |
| AVR , mean(SD) | 0.688±0.078 | 0.735±0.090 | 3.786 | <0.0001 |  | 0.669±0.085 | 0.731±0.092 | 3.574 | 0.001 |
| AVR <sub>0.5-1.0PD</sub> | 0.599±0.147 | 0.635±0.111 | 1.752 | 0.081 |  | 0.555±0.172 | 0.633±0.119 | 2.680 | 0.009 |
| AVR <sub>1.0-1.5PD</sub> | 0.592±0.123 | 0.655±0.108 | 3.559 | <0.0001 |  | 0.555±0.151 | 0.647±0.107 | 3.600 | <0.0001 |
| AVR <sub>1.5-2.0PD</sub> | 0.639±0.132 | 0.711±0.096 | 3.970 | <0.0001 |  | 0.622±0.159 | 0.715±0.101 | 3.552 | 0.001 |
| AVR <sub>2.0-2.5PD</sub> | 0.656±0.123 | 0.735±0.112 | 4.384 | <0.0001 |  | 0.638±0.150 | 0.731±0.121 | 3.514 | 0.001 |
| DA (μm^2^), mean(SD) | 1099457.427±1302481.675 | 1986200.621±1462014.412 | 4.286 | <0.0001 |  | 1161562.922±1282818.441 | 2143982.595±1473208.108 | 3.627 | <0.0001 |
| HDD (μm), mean(SD) | 1613.739±252.874 | 1696.470±330.877 | 1.916 | 0.057 |  | 1587.782±283.229 | 1727.364±340.647 | 2.272 | 0.025 |
| VDD (μm), mean(SD) | 1752.685±226.887 | 1861.218±315.431 | 2.510 | 0.013 |  | 1730.606±243.201 | 1906.133±320.204 | 3.148 | 0.002 |
| ODR, mean(SD) | 0.846±0.104 | 0.844±0.065 | -0.170 | 0.865 |  | 0.829±0.129 | 0.848±0.066 | 0.978 | 0.330 |
| ODT (D), mean(SD) | -29.929±63.637 | -12.322±70.020 | 1.716 | 0.088 |  | -22.806±63.001 | -5.688±72.091 | 1.289 | 0.200 |
| MRW (μm), mean(SD) | 2726.963±674.836 | 2940.621±799.903 | 1.945 | 0.053 |  | 2709.504±760.045 | 2892.108±865.906 | 1.143 | 0.256 |
| DDM (μm), mean(SD) | 4290.459±343.555 | 4501.746±446.549 | 3.394 | 0.001 |  | 4288.425±318.529 | 4569.660±462.061 | 3.614 | <0.0001 |
| ODAR, mean(SD) | 1.162±0.236 | 1.156±0.111 | -0.207 | 0.836 |  | 1.196±0.311 | 1.157±0.121 | -0.840 | 0.404 |
| MFD (μm), mean(SD) | 4652.525±893.021 | 4203.845±1244.646 | -2.632 | 0.010 |  | 4546.773±959.237 | 4251.346±1196.246 | -1.389 | 0.168 |
| OCA (μm^2^), mean(SD) | 276248.915±374516.702 | 560145.277±561921.827 | 3.751 | <0.0001 |  | 272850.359±341856.596 | 618911.124±600415.055 | 3.612 | 0.001 |
| CHD (μm), mean(SD) | 784.480±227.810 | 867.120±326.431 | 1.860 | 0.066 |  | 729.957±208.968 | 889.164±348.817 | 2.823 | 0.006 |
| CVD (μm), mean(SD) | 793.916±221.064 | 866.947±311.772 | 1.715 | 0.089 |  | 729.239±193.505 | 886.399±334.176 | 2.935 | 0.004 |
| CIR-C, mean(SD) | 0.855±0.111 | 0.856±0.060 | 0.036 | 0.971 |  | 0.841±0.126 | 0.860±0.051 | 1.022 | 0.311 |
| CDR-A, mean(SD) | 0.227±0.088 | 0.238±0.105 | 0.823 | 0.412 |  | 0.199±0.071 | 0.239±0.109 | 2.227 | 0.029 |
| HCDR, mean(SD) | 0.480±0.098 | 0.497±0.113 | 1.058 | 0.291 |  | 0.454±0.090 | 0.498±0.116 | 2.162 | 0.033 |
| VCDR, mean(SD) | 0.448±0.091 | 0.454±0.101 | 0.401 | 0.689 |  | 0.419±0.076 | 0.451±0.106 | 1.812 | 0.073 |
| Rim-I (μm), mean(SD) | 466.615±88.754 | 487.119±89.565 | 1.519 | 0.131 |  | 480.048±93.521 | 501.299±84.386 | 1.217 | 0.227 |
| Rim-N (μm), mean(SD) | 451.514±109.191 | 450.873±111.446 | -0.038 | 0.969 |  | 448.099±130.710 | 460.489±109.564 | 0.524 | 0.602 |
| Rim-S (μm), mean(SD) | 501.137±101.519 | 520.081±85.654 | 1.304 | 0.194 |  | 519.070±105.060 | 530.378±84.762 | 0.604 | 0.547 |
| Rim-T (μm), mean(SD) | 377.856±116.307 | 374.690±96.549 | -0.191 | 0.849 |  | 406.393±121.774 | 376.020±97.454 | -1.404 | 0.163 |
| Max Rim-Cup D (μm), mean(SD) | 1385.013±217.376 | 1476.322±304.144 | 2.194 | 0.030 |  | 1354.773±219.007 | 1502.613±326.093 | 2.714 | 0.008 |
| Min Rim (μm), mean(SD) | 304.968±92.473 | 314.720±83.544 | 0.721 | 0.472 |  | 307.324±106.199 | 320.675±84.101 | 0.711 | 0.479 |

MBA, Mean Branching Angle; FD-RVN,Fractal Dimension of the Retinal Vascular Network; MRVC(Caliber) , Mean Retinal Vessel Caliber; MRVC(Caliber)<sub>0.5-1.0PD</sub>, Mean Retinal Vessel Caliber within 0.5-1.0 PD Zone; MRV(Vessel)T, Mean Retinal Vessel Tortuosity; MRVκ<sub>0.5-1.0PD</sub>, Mean Retinal Vessel Curvature within 0.5-1.0 PD Zone; MRAT, Mean Retinal Arteriolar Tortuosity; MRAC(Curvature)<sub>0.5-1.0PD</sub>, Mean Retinal Arteriolar Curvature within 0.5-1.0 PD Zone; MRV(Venular)T, Mean Retinal Venular Tortuosity; MRVC(Curvature)<sub>0.5-1.0PD</sub> ,Mean Retinal Venular Curvature within 0.5-1.0 PD Zone; VD, Vessel Density; MRAC, Mean Retinal Arteriolar Caliber ; MRAC(Caliber)<sub>0.5-1.0PD</sub> , Mean Retinal Arteriolar Caliber within 0.5-1.0 PD Zone; MRV(Venular)C, Mean Retinal Venular Caliber ; MRV(Venular)C<sub>0.5-1.0PD</sub>, Mean Retinal Venular Caliber within 0.5-1.0 PD Zone; AVR, Arteriolar-to-Venular Ratio; DA, Optic Disc Area; HDD, Horizontal Diameter of the Optic Disc; VDD, Vertical Optic Disc Diameter; ODR, Optic Disc Roundness; ODT (D), Optic Disc Tilt; MRW, Minimum Rim Width; DDM, Disc-Macula Distance; ODAR, Optic Disc Axial Ratio; MFD, Macular Fovea Distance; OCA, Optic Cup Area; CHD, Cup Horizontal Diameter; CVD, Cup Vertical Diameter; CIR-C, Cup Circularity; CDR-A, Cup-to-Disc Area Ratio; HCDR, Horizontal Cup-to-Disc Ratio; VCDR, Vertical Cup-to-Disc Ratio; Rim-I, Inferior Neuroretinal Rim Width; Rim-N, Nasal Neuroretinal Rim Width; Rim-S, Superior Neuroretinal Rim Width; Rim-T, Temporal Neuroretinal Rim Width; Max Rim-Cup D, Maximum Rim-to-Cup Distance; Min Rim, Minimum Neuroretinal Rim Width; Data are mean±SD or n (%). Continuous and categorical variables were compared using the independent samples t-test/Mann-Whitney U test and Chi-squared/Fisher's exact test, respectively. P < 0.05 was considered significant.

Supplemental Table 3. Demographic and clinical characteristics of T2DM with HTN group and CHD with T2DM and HTN group participants.

|  | **Primary cohort** | | | |  | **PSM cohort** | | | |
| --- | --- | --- | --- | --- | --- | --- | --- | --- | --- |
|  | **CHD with HTN and T2DM** | **HTN and T2DM** | B | **P value** |  | **CHD with HTN and T2DM** | **HTN and T2DM** | B | **P value** |
| N | 127 | 45 |  |  |  | 31 | 31 |  |  |
| Age (years), mean(SD) | 61.92±10.079 | 65.53±110.114 | 0.921 | 0.358 |  | 64.032±10.886 | 63.387±9.608 | -0.247 | 0.805 |
| Sex |  |  | 21.426 | <0.001 |  |  |  | 0.067 | 0.796 |
| Male, n(%) | 87(68.5) | 13(28.9) |  |  |  | 18(58.1) | 19(61.3) |  |  |
| Female, n(%) | 40(31.5) | 32(71.1) |  |  |  | 13(41.9) | 12(38.7) |  |  |
| Smoking history, n(%) |  |  | 14.453 | <0.001 |  |  |  | 0.081 | 0.776 |
| No smoking | 57(44.9) | 35(77.8) |  |  |  | 22(71) | 23(74.2) |  |  |
| Still smoking | 70((55.1) | 10(22.2) |  |  |  | 9(29) | 8(25.8) |  |  |
| Drinking history, n(%) |  |  | 7.039 | 0.008 |  |  |  | - | 0.694 |
| No drinking | 95((74.8) | 42((93.3) |  |  |  | 29(93.5) | 29(93.5) |  |  |
| Still drinking | 32((25.2) | 3(6.7) |  |  |  | 2(6.5) | 2(6.5) |  |  |
| FBG (mmol/L), mean(SD) | 7.365±2.705 | 7.709±2.368 | 0.755 | 0.451 |  | 7.206±2.527 | 7.745±2.107 | 0.912 | 0.366 |
| Cr (μmol/L), mean(SD) | 84.139±76.624 | 66.8±26.108 | -1.523 | 0.130 |  | 70.052±18.252 | 72.194±29.566 | 0.343 | 0.733 |
| Lp(a) (mmol/L), mean(SD) | 25.08±28.775 | 20.938±22.636 | -0.874 | 0.383 |  | 20.639±27.408 | 21.358±22.282 | 0.113 | 0.910 |
| TG (mmol/L), mean(SD) | 1.72±1.368 | 2.10±3.163 | 1.097 | 0.274 |  | 1.521±1.280 | 1.577±1.363 | 0.167 | 0.868 |
| TC (mmol/L), mean(SD) | 3.628±1.048 | 4.605±1.271 | 5.069 | <0.001 |  | 4.155±1.173 | 4.215±0.856 | 0.230 | 0.819 |
| LDL (mmol/L), mean(SD) | 1.910±0.927 | 2.568±1.022 | 3.981 | <0.001 |  | 2.376±1.074 | 2.364±0.807 | -0.052 | 0.959 |
| HDL (mmol/L), mean(SD) | 1.123±0.230 | 1.281±0.331 | 2.959 | 0.004 |  | 1.204±0.325 | 1.246±0.345 | 0.504 | 0.616 |
| HbA1c (%), mean(SD) | 7.303±1.164 | 7.027±1.184 | -1.363 | 0.175 |  | 6.706±0.660 | 7.006±1.051 | 1.345 | 0.184 |
| MBA, mean(SD) | 62.834±9.812 | 57.683±11.562 | -2.886 | 0.004 |  | 62.507±11.217 | 56.714±12.124 | -1.953 | 0.055 |
| FD-RVN (×10), mean(SD) | 1.475±0.074 | 1.452±0.061 | -1.892 | 0.060 |  | 1.467±0.078 | 1.450±0.059 | -0.972 | 0.335 |
| MRVC(Caliber) (μm), mean(SD) | 72.880±8.724 | 79.383±10.717 | 3.663 | <0.001 |  | 73.502±11.215 | 80.963±11.205 | 2.620 | 0.011 |
| MRVC(Caliber)<sub>0.5–1.0PD</sub> | 88.159±13.326 | 91.844±13.411 | 1.591 | 0.113 |  | 87.731±11.953 | 92.799±14.714 | 1.489 | 0.142 |
| MRVC(Caliber)<sub>1.0–1.5PD</sub> | 87.451±10.733 | 91.613±11.790 | 2.178 | 0.031 |  | 88.007±12.608 | 91.411±12.041 | 1.087 | 0.281 |
| MRVC(Caliber)<sub>1.5–2.0PD</sub> | 83.194±10.187 | 87.400±11.291 | 2.312 | 0.022 |  | 81.500±8.522 | 88.348±12.763 | 2.484 | 0.016 |
| MRVC(Caliber)<sub>2.0–2.5PD</sub> | 78.453±9.799 | 83.209±13.828 | 2.496 | 0.014 |  | 76.778±10.899 | 84.433±15.463 | 2.253 | 0.028 |
| MRV(Vessel)T (×10^4^) , mean(SD) | 0.0008±0.0002 | 0.0007±0.0002 | -1.643 | 0.102 |  | 0.0008±0.0001 | 0.0007±0.0002 | -1.672 | 0.100 |
| MRVκ<sub>0.5–1.0PD</sub> | 0.0007±0.0002 | 0.0007±0.0002 | -0.867 | 0.387 |  | 0.0007±0.0002 | 0.0007±0.0002 | 0.675 | 0.502 |
| MRVκ<sub>1.0–1.5PD</sub> | 0.0007±0.0002 | 0.0007±0.0002 | -1.205 | 0.230 |  | 0.0007±0.0002 | 0.0007±0.0002 | -0.593 | 0.556 |
| MRVκ<sub>1.5–2.0PD</sub> | 0.0008±0.0002 | 0.0007±0.0003 | -0.746 | 0.457 |  | 0.0008±0.0002 | 0.0007±0.0002 | -1.498 | 0.139 |
| MRVκ<sub>2.0–2.5PD</sub> | 0.0008±0.0002 | 0.0007±0.0002 | -2.389 | 0.018 |  | 0.0008±0.0002 | 0.0007±0.0002 | -2.042 | 0.046 |
| MRAT (×10^4^), mean(SD) | 0.0006±0.0002 | 0.0006±0.0002 | -1.090 | 0.277 |  | 0.0006±0.0002 | 0.0006±0.0002 | -1.643 | 0.106 |
| MRAC(Curvature)<sub>0.5-1.0PD</sub> | 0.0006±0.0003 | 0.0006±0.0002 | -0.446 | 0.656 |  | 0.0007±0.0004 | 0.0006±0.0002 | -0.948 | 0.347 |
| MRAC(Curvature)<sub>1.0-1.5PD</sub> | 0.0006±0.0002 | 0.0006±0.0003 | -0.697 | 0.487 |  | 0.0006±0.0003 | 0.0006±0.0004 | -0.727 | 0.470 |
| MRAC(Curvature)<sub>1.5-2.0PD</sub> | 0.0006±0.0003 | 0.0005±0.0003 | -1.366 | 0.174 |  | 0.0006±0.0003 | 0.0005±0.0002 | -1.548 | 0.127 |
| MRAC(Curvature)<sub>2.0-2.5PD</sub> | 0.0006±0.0002 | 0.0006±0.0002 | -1.093 | 0.276 |  | 0.0006±0.0002 | 0.0005±0.0002 | -1.090 | 0.280 |
| MRV(Venular)T (×10^4^), mean(SD) | 0.0009±0.0002 | 0.0008±0.0002 | -1.714 | 0.088 |  | 0.0009±0.0001 | 0.0008±0.0002 | -1.305 | 0.197 |
| MRVC(Curvature)<sub>0.5-1.0PD</sub> | 0.0008±0.0002 | 0.0008±0.0002 | -1.179 | 0.240 |  | 0.0007±0.0002 | 0.0008±0.0002 | 1.383 | 0.172 |
| MRVC(Curvature)<sub>1.0-1.5PD</sub> | 0.0008±0.0003 | 0.0007±0.0003 | -1.363 | 0.175 |  | 0.0007±0.0002 | 0.0007±0.0003 | -0.359 | 0.721 |
| MRVC(Curvature)<sub>1.5-2.0PD</sub> | 0.0008±0.0003 | 0.0008±0.0003 | -0.653 | 0.515 |  | 0.0009±0.0003 | 0.0008±0.0003 | -1.253 | 0.215 |
| MRVC(Curvature)<sub>2.0-2.5PD</sub> | 0.0009±0.0003 | 0.0008±0.0003 | -2.490 | 0.014 |  | 0.0009±0.0003 | 0.0008±0.0003 | -1.699 | 0.095 |
| VD (×10^2^), mean(SD) | 0.062±0.200 | 0.055±0.017 | -1.992 | 0.048 |  | 0.058±0.021 | 0.055±0.017 | -0.729 | 0.469 |
| MRAC (μm) , mean(SD) | 56.154±4.703 | 63.599±9.224 | 5.181 | <0.001 |  | 56.235±4.825 | 65.332±8.950 | 4.981 | 0.000 |
| MRAC(Caliber)<sub>0.5-1.0PD</sub> | 62.134±8.122 | 68.103±10.816 | 3.380 | 0.001 |  | 60.715±8.075 | 68.153±11.296 | 2.983 | 0.004 |
| MRAC(Caliber)<sub>1.0-1.5PD</sub> | 61.508±8.655 | 67.665±10.150 | 3.915 | <0.001 |  | 61.690±8.482 | 69.123±10.186 | 3.122 | 0.003 |
| MRAC(Caliber)<sub>1.5-2.0PD</sub> | 60.510±8.063 | 67.725±11.772 | 3.807 | <0.001 |  | 60.004±9.401 | 68.926±12.947 | 3.105 | 0.003 |
| MRAC(Caliber)<sub>2.0-2.5PD</sub> | 58.269±7.655 | 66.380±14.2021 | 3.648 | 0.001 |  | 56.853±8.453 | 68.171±14.849 | 3.688 | 0.000 |
| MRV(Venular)C (μm), mean(SD) | 84.764±9.651 | 89.904±11.378 | 2.705 | 0.009 |  | 85.149±14.037 | 91.357±12.187 | 1.859 | 0.068 |
| MRV(Venular)C<sub>0.5-1.0PD</sub> | 106.557±15.092 | 111.163±15.597 | 1.744 | 0.083 |  | 107.080±15.879 | 114.054±16.113 | 1.716 | 0.091 |
| MRV(Venular)C<sub>1.0-1.5PD</sub> | 104.073±12.460 | 108.818±12.884 | 2.176 | 0.031 |  | 103.380±15.238 | 109.898±13.351 | 1.791 | 0.078 |
| MRV(Venular)C<sub>1.5-2.0PD</sub> | 98.062±12.135 | 99.701±10.791 | 0.801 | 0.424 |  | 93.717±10.951 | 101.022±11.717 | 2.536 | 0.014 |
| MRV(Venular)C<sub>2.0-2.5PD</sub> | 90.202±12.284 | 94.091±15.952 | 1.682 | 0.094 |  | 87.103±12.536 | 95.389±18.130 | 2.093 | 0.041 |
| AVR , mean(SD) | 0.669±0.079 | 0.710±0.080 | 3.002 | 0.003 |  | 0.673±0.096 | 0.720±0.080 | 2.078 | 0.042 |
| AVR <sub>0.5-1.0PD</sub> | 0.572±0.140 | 0.602±0.138 | 1.230 | 0.220 |  | 0.576±0.094 | 0.604±0.107 | 1.090 | 0.280 |
| AVR <sub>1.0-1.5PD</sub> | 0.588±0.125 | 0.627±0.095 | 1.883 | 0.061 |  | 0.594±0.147 | 0.634±0.097 | 1.293 | 0.201 |
| AVR <sub>1.5-2.0PD</sub> | 0.613±0.127 | 0.680±0.091 | 3.256 | 0.001 |  | 0.627±0.168 | 0.683±0.099 | 1.601 | 0.115 |
| AVR <sub>2.0-2.5PD</sub> | 0.650±0.128 | 0.707±0.114 | 2.615 | 0.010 |  | 0.665±0.128 | 0.718±0.117 | 1.702 | 0.094 |
| DA (μm^2^), mean(SD) | 1033873.88±1246963.83 | 1562540.80±1300112.31 | 2.417 | 0.017 |  | 1187932.79±1462490.94 | 1774403.39±1273221.67 | 1.684 | 0.097 |
| HDD (μm), mean(SD) | 1596.932±250.940 | 1632.461±340.268 | 0.740 | 0.460 |  | 1653.840±282.069 | 1642.685±362.418 | -0.135 | 0.893 |
| VDD (μm), mean(SD) | 1744.079±231.341 | 1782.400±367.855 | 0.808 | 0.420 |  | 1866.668±244.090 | 1808.421±420.314 | -0.667 | 0.507 |
| ODR, mean(SD) | 0.836±0.079 | 0.845±0.0735 | 0.649 | 0.517 |  | 0.816±0.098 | 0.845±0.059 | 1.392 | 0.169 |
| ODT (D), mean(SD) | -29.524±63.467 | -6.145±72.064 | 1.927 | 0.058 |  | -25.039±69.257 | -1.893±75.757 | 1.256 | 0.214 |
| MRW (μm), mean(SD) | 2783.321±680.555 | 2795.902±640.189 | 0.108 | 0.914 |  | 2626.410±854.509 | 2818.563±751.162 | 0.940 | 0.351 |
| DDM (μm), mean(SD) | 4276.037±281.699 | 4451.095±387.090 | 2.784 | 0.007 |  | 4363.603±262.593 | 4494.350±418.938 | 1.472 | 0.147 |
| ODAR, mean(SD) | 1.167±0.149 | 1.1569±0.132 | -0.399 | 0.690 |  | 1.214±0.207 | 1.1528±0.091 | -1.502 | 0.138 |
| MFD (μm), mean(SD) | 4686.727±825.089 | 4351.622±1169.941 | -1.772 | 0.082 |  | 4541.662±1135.860 | 4168.300±1345.381 | -1.181 | 0.242 |
| OCA (μm^2^), mean(SD) | 268426.982±400560.725 | 380758.998±376832.325 | 1.641 | 0.103 |  | 347787.13±545108.74 | 420394.43±352792.35 | 0.623 | 0.536 |
| CHD (μm), mean(SD) | 804.158±241.865 | 844.793±228.367 | 0.982 | 0.327 |  | 875.529±289.822 | 840.679±201.712 | -0.550 | 0.585 |
| CVD (μm), mean(SD) | 815.745±222.757 | 840.613±227.869 | 0.640 | 0.523 |  | 907.809±242.421 | 852.773±226.095 | -0.924 | 0.359 |
| CIR-C, mean(SD) | 0.859±0.069 | 0.852±0.060 | -0.618 | 0.537 |  | 0.830±0.093 | 0.858±0.059 | 1.459 | 0.150 |
| CDR-A, mean(SD) | 0.238±0.087 | 0.238±0.086 | -0.058 | 0.954 |  | 0.262±0.091 | 0.231±0.082 | -1.399 | 0.167 |
| HCDR, mean(SD) | 0.496±0.100 | 0.502±0.100 | 0.312 | 0.755 |  | 0.522±0.096 | 0.495±0.099 | -1.086 | 0.282 |
| VCDR, mean(SD) | 0.461±0.086 | 0.456±0.083 | -0.347 | 0.729 |  | 0.482±0.084 | 0.452±0.077 | -1.430 | 0.158 |
| Rim-I (μm), mean(SD) | 451.459±84.125 | 466.323±87.167 | 1.009 | 0.314 |  | 472.893±80.694 | 479.965±95.841 | 0.314 | 0.754 |
| Rim-N (μm), mean(SD) | 435.788±111.395 | 423.488±107.141 | -0.643 | 0.521 |  | 427.781±107.200 | 428.006±117.304 | 0.008 | 0.994 |
| Rim-S (μm), mean(SD) | 484.545±85.760 | 516.847±91.784 | 2.131 | 0.034 |  | 495.077±91.463 | 533.092±97.870 | 1.580 | 0.119 |
| Rim-T (μm), mean(SD) | 352.708±102.225 | 395.355±113.809 | 2.334 | 0.021 |  | 336.919±129.291 | 420.143±123.559 | 2.591 | 0.012 |
| Max Rim-Cup D (μm), mean(SD) | 1388.280±227.469 | 1425.170±224.490 | 0.938 | 0.350 |  | 1497.328±251.387 | 1445.112±224.826 | -0.862 | 0.392 |
| Min Rim (μm), mean(SD) | 295.420±78.862 | 320.759±69.729 | 1.907 | 0.058 |  | 270.822±97.835 | 332.154±77.130 | 2.741 | 0.008 |

MBA, Mean Branching Angle; FD-RVN,Fractal Dimension of the Retinal Vascular Network; MRVC(Caliber) , Mean Retinal Vessel Caliber; MRVC(Caliber)<sub>0.5-1.0PD</sub>, Mean Retinal Vessel Caliber within 0.5-1.0 PD Zone; MRV(Vessel)T, Mean Retinal Vessel Tortuosity; MRVκ<sub>0.5-1.0PD</sub>, Mean Retinal Vessel Curvature within 0.5-1.0 PD Zone; MRAT, Mean Retinal Arteriolar Tortuosity; MRAC(Curvature)<sub>0.5-1.0PD</sub>, Mean Retinal Arteriolar Curvature within 0.5-1.0 PD Zone; MRV(Venular)T, Mean Retinal Venular Tortuosity; MRVC(Curvature)<sub>0.5-1.0PD</sub> ,Mean Retinal Venular Curvature within 0.5-1.0 PD Zone; VD, Vessel Density; MRAC, Mean Retinal Arteriolar Caliber ; MRAC(Caliber)<sub>0.5-1.0PD</sub> , Mean Retinal Arteriolar Caliber within 0.5-1.0 PD Zone; MRV(Venular)C, Mean Retinal Venular Caliber ; MRV(Venular)C<sub>0.5-1.0PD</sub>, Mean Retinal Venular Caliber within 0.5-1.0 PD Zone; AVR, Arteriolar-to-Venular Ratio; DA, Optic Disc Area; HDD, Horizontal Diameter of the Optic Disc; VDD, Vertical Optic Disc Diameter; ODR, Optic Disc Roundness; ODT (D), Optic Disc Tilt; MRW, Minimum Rim Width; DDM, Disc-Macula Distance; ODAR, Optic Disc Axial Ratio; MFD, Macular Fovea Distance; OCA, Optic Cup Area; CHD, Cup Horizontal Diameter; CVD, Cup Vertical Diameter; CIR-C, Cup Circularity; CDR-A, Cup-to-Disc Area Ratio; HCDR, Horizontal Cup-to-Disc Ratio; VCDR, Vertical Cup-to-Disc Ratio; Rim-I, Inferior Neuroretinal Rim Width; Rim-N, Nasal Neuroretinal Rim Width; Rim-S, Superior Neuroretinal Rim Width; Rim-T, Temporal Neuroretinal Rim Width; Max Rim-Cup D, Maximum Rim-to-Cup Distance; Min Rim, Minimum Neuroretinal Rim Width; Data are mean±SD or n (%). Continuous and categorical variables were compared using the independent samples t-test/Mann-Whitney U test and Chi-squared/Fisher's exact test, respectively. P < 0.05 was considered significant.

Supplemental Table 4. Demographic and clinical characteristics of T2DM group and CHD with T2DM group participants.

|  | **Primary cohort** | | | |  | **PSM cohort** | | | |
| --- | --- | --- | --- | --- | --- | --- | --- | --- | --- |
|  | **CHD with T2DM** | **T2DM** | **B** | **P value** |  | **CHD with T2DM** | **T2DM** | **B** | **P value** |
| N | 35 | 20 |  |  |  | 9 | 9 |  |  |
| Age (years), mean(SD) | 58.140±11.293 | 62.100±8.596 | 1.357 | 0.181 |  | 62.670±7.762 | 64.330±9.695 | 0.403 | 0.693 |
| Sex |  |  | 1.650 | 0.199 |  |  |  | - | 0.681 |
| Male, n(%) | 13(37.1) | 11(55) |  |  |  | 4(44.4) | 4(44.4) |  |  |
| Female, n(%) | 22(62.9) | 9(45) |  |  |  | 5(55.6) | 5(55.6) |  |  |
| Smoking history, n(%) |  |  | 0.025 | 0.874 |  |  |  | 3.600 | 0.058 |
| No smoking | 22(62.9) | 13(65) |  |  |  | 9(100) | 6(66.7) |  |  |
| Still smoking | 13(37.1) | 7(35) |  |  |  | 0(0) | 3(33.3) |  |  |
| Drinking history, n(%) |  |  | 1.713 | **0.191** |  |  |  | 2.225 | 0.134 |
| No drinking | 31(88.6) | 15(75) |  |  |  | 9(100) | 7(77.8) |  |  |
| Still drinking | 4(11.4) | 5(25) |  |  |  | 0(0) | 2(22.2) |  |  |
| FBG (mmol/L), mean(SD) | 7.811±2.972 | 7.710±2.119 | -0.134 | 0.894 |  | 7.233±2.929 | 7.867±2.294 | 0.511 | 0.617 |
| Cr (μmol/L), mean(SD) | 68.600±22.373 | 66.550±10.971 | -0.383 | 0.703 |  | 67.220±14.078 | 61.780±6.996 | -1.039 | 0.314 |
| Lp(a) (mmol/L), mean(SD) | 29.666±28.531 | 21.235±21.739 | -1.144 | 0.258 |  | 24.356±32.424 | 26.111±27.970 | 0.123 | 0.903 |
| TG (mmol/L), mean(SD) | 1.423±1.244 | 1.874±1.420 | 1.227 | 0.225 |  | 1.453±2.190 | 1.668±1.406 | 0.247 | 0.808 |
| TC (mmol/L), mean(SD) | 3.615±1.058 | 4.649±1.303 | 3.200 | 0.002 |  | 3.830±1.189 | 3.937±0.737 | 0.229 | 0.822 |
| LDL (mmol/L), mean(SD) | 1.937±0.887 | 2.765±1.061 | 3.098 | 0.003 |  | 1.980±0.923 | 2.150±0.614 | 0.460 | 0.652 |
| HDL (mmol/L), mean(SD) | 1.167±0.339 | 1.304±0.354 | 1.419 | 0.162 |  | 1.399±0.436 | 1.243±0.314 | -0.868 | 0.398 |
| HbA1c (%), mean(SD) | 7.800±1.030 | 7.110±0.795 | -2.585 | 0.013 |  | 7.522±1.066 | 7.367±1.001 | -0.319 | 0.754 |
| MBA, mean(SD) | 56.425±12.895 | 59.761±10.056 | 0.995 | 0.324 |  | 59.884±8.589 | 55.786±12.481 | -0.811 | 0.429 |
| FD-RVN (×10), mean(SD) | 1.436±0.067 | 1.498±0.038 | 4.383 | 0.000 |  | 1.409±0.085 | 1.483±0.049 | 2.259 | 0.038 |
| MRVC(Caliber) (μm), mean(SD) | 86.761±9.513 | 77.387±8.847 | -3.604 | 0.001 |  | 88.625±9.279 | 81.380±9.482 | -1.638 | 0.121 |
| MRVC(Caliber)<sub>0.5–1.0PD</sub> | 97.759±9.826 | 92.138±9.713 | -2.049 | 0.045 |  | 95.092±10.370 | 94.433±12.723 | -0.120 | 0.906 |
| MRVC(Caliber)<sub>1.0–1.5PD</sub> | 97.100±9.258 | 90.297±9.316 | -2.616 | 0.012 |  | 98.816±10.217 | 93.393±11.417 | -1.062 | 0.304 |
| MRVC(Caliber)<sub>1.5–2.0PD</sub> | 91.374±9.779 | 85.550±8.042 | -2.260 | 0.028 |  | 95.955±12.100 | 89.733±8.869 | -1.244 | 0.231 |
| MRVC(Caliber)<sub>2.0–2.5PD</sub> | 85.519±10.247 | 81.076±8.468 | -1.643 | 0.106 |  | 85.435±14.244 | 85.234±7.154 | -0.038 | 0.970 |
| MRV(Vessel)T (×10^4^) , mean(SD) | 0.0007±0.0001 | 0.0007±0.0002 | 1.519 | 0.135 |  | 0.0007±0.0001 | 0.0007±0.0001 | 0.438 | 0.667 |
| MRVκ<sub>0.5–1.0PD</sub> | 0.0006±0.0001 | 0.0007±0.0002 | 2.156 | 0.036 |  | 0.0007±0.0002 | 0.0007±0.0002 | 0.176 | 0.862 |
| MRVκ<sub>1.0–1.5PD</sub> | 0.0006±0.0002 | 0.0006±0.0001 | 0.590 | 0.557 |  | 0.0006±0.0002 | 0.0006±0.0002 | 0.077 | 0.940 |
| MRVκ<sub>1.5–2.0PD</sub> | 0.0006±0.0002 | 0.0007±0.0002 | 1.463 | 0.149 |  | 0.0007±0.0002 | 0.0007±0.0002 | 0.269 | 0.792 |
| MRVκ<sub>2.0–2.5PD</sub> | 0.0006±0.0001 | 0.0007±0.0002 | 1.377 | 0.179 |  | 0.0006±0.0001 | 0.0006±0.0002 | 0.338 | 0.740 |
| MRAT (×10^4^), mean(SD) | 0.0006±0.0001 | 0.0006±0.0002 | 0.772 | 0.443 |  | 0.0006±0.0002 | 0.0006±0.0002 | -0.130 | 0.898 |
| MRAC(Curvature)<sub>0.5-1.0PD</sub> | 0.0005±0.0002 | 0.0006±0.0003 | 1.152 | 0.259 |  | 0.0006±0.0002 | 0.0006±0.0003 | -0.056 | 0.956 |
| MRAC(Curvature)<sub>1.0-1.5PD</sub> | 0.0005±0.0003 | 0.0006±0.0002 | 0.827 | 0.412 |  | 0.0005±0.0002 | 0.0006±0.0002 | 1.573 | 0.135 |
| MRAC(Curvature)<sub>1.5-2.0PD</sub> | 0.0005±0.0003 | 0.0006±0.0002 | 1.323 | 0.192 |  | 0.0005±0.0003 | 0.0006±0.0002 | 0.850 | 0.408 |
| MRAC(Curvature)<sub>2.0-2.5PD</sub> | 0.0006±0.0004 | 0.0006±0.0002 | 0.114 | 0.910 |  | 0.0007±0.0007 | 0.0005±0.0002 | -0.710 | 0.488 |
| MRV(Venular)T (×10^4^), mean(SD) | 0.0007±0.0002 | 0.0008±0.0002 | 1.535 | 0.131 |  | 0.0007±0.0002 | 0.0007±0.0002 | 0.366 | 0.720 |
| MRVC(Curvature)<sub>0.5-1.0PD</sub> | 0.0007±0.0003 | 0.0008±0.0002 | 1.287 | 0.204 |  | 0.0009±0.0005 | 0.0008±0.0001 | -0.261 | 0.797 |
| MRVC(Curvature)<sub>1.0-1.5PD</sub> | 0.0007±0.0002 | 0.0007±0.0002 | 0.003 | 0.998 |  | 0.0007±0.0003 | 0.0006±0.0002 | -0.544 | 0.594 |
| MRVC(Curvature)<sub>1.5-2.0PD</sub> | 0.0007±0.0002 | 0.0008±0.0003 | 1.323 | 0.191 |  | 0.0007±0.0002 | 0.0008±0.0003 | 0.153 | 0.880 |
| MRVC(Curvature)<sub>2.0-2.5PD</sub> | 0.0006±0.0002 | 0.0007±0.0002 | 1.481 | 0.145 |  | 0.0006±0.0001 | 0.0006±0.0001 | 0.903 | 0.380 |
| VD (×10^2^), mean(SD) | 0.050±0.017 | 0.067±0.015 | 3.709 | <0.0001 |  | 0.045±0.021 | 0.065±0.019 | 2.096 | 0.052 |
| MRAC (μm) , mean(SD) | 68.785±7.935 | 63.807±7.329 | -2.299 | 0.025 |  | 72.537±9.147 | 66.223±6.808 | -1.661 | 0.116 |
| MRAC(Caliber)<sub>0.5-1.0PD</sub> | 71.469±11.428 | 69.883±8.619 | -0.539 | 0.592 |  | 74.999±8.962 | 71.330±11.537 | -0.753 | 0.462 |
| MRAC(Caliber)<sub>1.0-1.5PD</sub> | 73.019±9.502 | 70.727±11.031 | -0.812 | 0.421 |  | 74.984±10.258 | 74.139±12.656 | -0.156 | 0.878 |
| MRAC(Caliber)<sub>1.5-2.0PD</sub> | 69.886±9.563 | 67.920±7.167 | -0.799 | 0.428 |  | 74.010±10.953 | 69.715±7.536 | -0.969 | 0.347 |
| MRAC(Caliber)<sub>2.0-2.5PD</sub> | 65.633±8.643 | 64.495±8.480 | -0.473 | 0.638 |  | 65.160±10.461 | 67.790±4.990 | 0.681 | 0.506 |
| MRV(Venular)C (μm), mean(SD) | 100.828±11.633 | 90.180±10.596 | -3.370 | 0.001 |  | 100.432±7.624 | 94.736±12.126 | -1.193 | 0.250 |
| MRV(Venular)C<sub>0.5-1.0PD</sub> | 121.347±17.748 | 113.293±14.404 | -1.728 | 0.090 |  | 120.746±21.903 | 117.374±17.237 | -0.363 | 0.721 |
| MRV(Venular)C<sub>1.0-1.5PD</sub> | 114.736±12.160 | 108.469±10.948 | -1.904 | 0.062 |  | 114.164±11.871 | 113.172±10.458 | -0.188 | 0.853 |
| MRV(Venular)C<sub>1.5-2.0PD</sub> | 105.801±11.430 | 100.355±10.029 | -1.775 | 0.082 |  | 105.964±10.235 | 106.548±9.904 | 0.123 | 0.904 |
| MRV(Venular)C<sub>2.0-2.5PD</sub> | 100.307±15.131 | 96.832±12.591 | -0.869 | 0.389 |  | 98.186±19.398 | 102.336±9.960 | 0.571 | 0.576 |
| AVR , mean(SD) | 0.685±0.067 | 0.710±0.066 | 1.341 | 0.186 |  | 0.722±0.069 | 0.703±0.061 | -0.626 | 0.540 |
| AVR <sub>0.5-1.0PD</sub> | 0.594±0.115 | 0.621±0.069 | 0.956 | 0.343 |  | 0.641±0.145 | 0.612±0.086 | -0.515 | 0.613 |
| AVR <sub>1.0-1.5PD</sub> | 0.625±0.144 | 0.652±0.080 | 0.778 | 0.440 |  | 0.610±0.252 | 0.656±0.098 | 0.504 | 0.621 |
| AVR <sub>1.5-2.0PD</sub> | 0.666±0.094 | 0.680±0.071 | 0.570 | 0.571 |  | 0.698±0.069 | 0.658±0.077 | -1.158 | 0.264 |
| AVR <sub>2.0-2.5PD</sub> | 0.669±0.107 | 0.672±0.088 | 0.101 | 0.920 |  | 0.675±0.109 | 0.665±0.040 | -0.263 | 0.796 |
| DA (μm^2^), mean(SD) | 2038635.387±1745552.179 | 1224730.655±1456960.381 | -1.762 | 0.084 |  | 1901008.217±1867602.794 | 1814988.394±1459344.560 | -0.109 | 0.915 |
| HDD (μm), mean(SD) | 1804.684±317.910 | 1656.866±242.251 | -1.800 | 0.078 |  | 1849.208±270.304 | 1686.860±327.993 | -1.146 | 0.268 |
| VDD (μm), mean(SD) | 1948.753±368.804 | 1821.800±202.890 | -1.647 | 0.106 |  | 1949.831±335.852 | 1828.286±234.740 | -0.890 | 0.387 |
| ODR, mean(SD) | 0.839±0.045 | 0.849±0.038 | 0.827 | 0.412 |  | 0.846±0.029 | 0.846±0.037 | -0.008 | 0.993 |
| ODT (D), mean(SD) | -19.436±68.108 | -34.044±71.724 | -0.751 | 0.456 |  | 0.940±68.289 | -48.893±59.250 | -1.654 | 0.118 |
| MRW (μm), mean(SD) | 3026.698±643.993 | 2768.847±467.979 | -1.567 | 0.123 |  | 2854.058±792.135 | 2811.243±521.343 | -0.135 | 0.894 |
| DDM (μm), mean(SD) | 4507.818±505.976 | 4480.767±408.714 | -0.204 | 0.839 |  | 4697.769±475.553 | 4472.152±322.376 | -1.178 | 0.256 |
| ODAR, mean(SD) | 1.149±0.072 | 1.138±0.056 | -0.600 | 0.551 |  | 1.141±0.059 | 1.138±0.060 | -0.111 | 0.913 |
| MFD (μm), mean(SD) | 4032.801±1274.272 | 4564.176±1016.625 | 1.595 | 0.117 |  | 4343.899±1056.944 | 4411.935±1421.849 | 0.115 | 0.910 |
| OCA (μm^2^), mean(SD) | -574022.383±6889681.632 | 297019.006±393657.397 | 0.563 | 0.576 |  | -4012110.816±13510260.990 | 444500.589±420314.103 | 0.989 | 0.352 |
| CHD (μm), mean(SD) | -1247.630±12825.384 | 762.313±232.815 | 0.698 | 0.488 |  | -7512.420±25284.294 | 827.038±273.735 | 0.989 | 0.351 |
| CVD (μm), mean(SD) | -1112.459±12154.221 | 777.012±216.580 | 0.692 | 0.492 |  | -7058.828±23956.171 | 835.180±270.965 | 0.988 | 0.352 |
| CIR-C, mean(SD) | 0.469±2.247 | 0.878±0.047 | 0.809 | 0.422 |  | -0.621±4.433 | 0.863±0.045 | 1.004 | 0.345 |
| CDR-A, mean(SD) | -0.672±5.421 | 0.202±0.089 | 0.718 | 0.476 |  | -3.337±10.683 | 0.230±0.110 | 1.002 | 0.346 |
| HCDR, mean(SD) | -0.465±5.684 | 0.455±0.104 | 0.721 | 0.474 |  | -3.254±11.203 | 0.485±0.123 | 1.001 | 0.346 |
| VCDR, mean(SD) | -0.633±6.522 | 0.422±0.087 | 0.720 | 0.475 |  | -3.825±12.858 | 0.448±0.106 | 0.997 | 0.348 |
| Rim-I (μm), mean(SD) | 503.030±104.802 | 530.139±81.620 | 0.996 | 0.324 |  | 502.983±124.046 | 502.948±75.957 | -0.001 | 0.999 |
| Rim-N (μm), mean(SD) | 463.510±107.582 | 498.695±103.252 | 1.184 | 0.242 |  | 484.010±159.545 | 447.306±102.699 | -0.580 | 0.570 |
| Rim-S (μm), mean(SD) | 511.280±105.558 | 532.338±78.636 | 0.776 | 0.441 |  | 516.086±137.027 | 504.578±77.867 | -0.219 | 0.829 |
| Rim-T (μm), mean(SD) | 417.434±130.242 | 392.538±126.325 | -0.689 | 0.494 |  | 443.228±111.676 | 407.244±167.449 | -0.536 | 0.599 |
| Max Rim-Cup D (μm), mean(SD) | 352.359±7225.436 | 1395.040±223.198 | 0.643 | 0.523 |  | -3151.200±14243.309 | 1434.022±288.961 | 0.966 | 0.363 |
| Min Rim (μm), mean(SD) | 971.221±3748.911 | 341.259±77.190 | -0.748 | 0.458 |  | 2859.396±7369.452 | 336.900±91.078 | -1.027 | 0.335 |

MBA, Mean Branching Angle; FD-RVN, Fractal Dimension of the Retinal Vascular Network; MRVC(Caliber) , Mean Retinal Vessel Caliber; MRVC(Caliber)<sub>0.5-1.0PD</sub>, Mean Retinal Vessel Caliber within 0.5-1.0 PD Zone; MRV(Vessel)T, Mean Retinal Vessel Tortuosity; MRVκ<sub>0.5-1.0PD</sub>, Mean Retinal Vessel Curvature within 0.5-1.0 PD Zone; MRAT, Mean Retinal Arteriolar Tortuosity; MRAC(Curvature)<sub>0.5-1.0PD</sub>, Mean Retinal Arteriolar Curvature within 0.5-1.0 PD Zone; MRV(Venular)T, Mean Retinal Venular Tortuosity; MRVC(Curvature)<sub>0.5-1.0PD</sub> ,Mean Retinal Venular Curvature within 0.5-1.0 PD Zone; VD, Vessel Density; MRAC, Mean Retinal Arteriolar Caliber ; MRAC(Caliber)<sub>0.5-1.0PD</sub> , Mean Retinal Arteriolar Caliber within 0.5-1.0 PD Zone; MRV(Venular)C, Mean Retinal Venular Caliber ; MRV(Venular)C<sub>0.5-1.0PD</sub>, Mean Retinal Venular Caliber within 0.5-1.0 PD Zone; AVR, Arteriolar-to-Venular Ratio; DA, Optic Disc Area; HDD, Horizontal Diameter of the Optic Disc; VDD, Vertical Optic Disc Diameter; ODR, Optic Disc Roundness; ODT (D), Optic Disc Tilt; MRW, Minimum Rim Width; DDM, Disc-Macula Distance; ODAR, Optic Disc Axial Ratio; MFD, Macular Fovea Distance; OCA, Optic Cup Area; CHD, Cup Horizontal Diameter; CVD, Cup Vertical Diameter; CIR-C, Cup Circularity; CDR-A, Cup-to-Disc Area Ratio; HCDR, Horizontal Cup-to-Disc Ratio; VCDR, Vertical Cup-to-Disc Ratio; Rim-I, Inferior Neuroretinal Rim Width; Rim-N, Nasal Neuroretinal Rim Width; Rim-S, Superior Neuroretinal Rim Width; Rim-T, Temporal Neuroretinal Rim Width; Max Rim-Cup D, Maximum Rim-to-Cup Distance; Min Rim, Minimum Neuroretinal Rim Width; Data are mean±SD or n (%). Continuous and categorical variables were compared using the independent samples t-test/Mann-Whitney U test and Chi-squared/Fisher's exact test, respectively. P < 0.05 was considered significant.

Supplemental Table 5. Clinical and demographic characteristics of hospitalized CHD patients with HTN and T2DM stratified by short-term SGLT2 inhibitor use versus non-use.

|  | **Primary cohort** | | | |  | **PSM cohort** | | | |
| --- | --- | --- | --- | --- | --- | --- | --- | --- | --- |
|  | **SGLT2i(Y)** | **SGLT2i(N)** | B | **P value** |  | **SGLT2i(Y)** | **SGLT2i(N)** | B | **P value** |
| N | 97 | 69 |  |  |  | 58 | 58 |  |  |
| Age (years), mean(SD) | 61.320±10.261 | 63.350±9.972 | 1.270 | 0.206 |  | 62.660±9.068 | 62.780±9.403 | 0.070 | 0.944 |
| Sex |  |  | 1.527 | 0.217 |  |  |  | 0.586 | 0.444 |
| Male, n(%) | 54(55.7) | 45(65.2) |  |  |  | 34(58.6) | 38(65.5) |  |  |
| Female, n(%) | 43(44.3) | 24(34.8) |  |  |  | 24(41.4) | 20(34.5) |  |  |
| Smoking history, n(%) |  |  | 4.309 | 0.038 |  |  |  | 1.690 | 0.194 |
| No smoking | 58(59.8) | 30(43.5) |  |  |  | 33(56.9) | 26(44.8) |  |  |
| Still smoking | 39(40.2) | 39(56.5) |  |  |  | 25(43.1) | 32(55.2) |  |  |
| Drinking history, n(%) |  |  | 2.278 | 0.131 |  |  |  | 0.793 | 0.373 |
| No drinking | 81(83.5) | 51(73.9) |  |  |  | 47(81) | 43(74.1) |  |  |
| Still drinking | 16(16.5) | 18(26.1) |  |  |  | 11(19) | 15(25.9) |  |  |
| CHD, n(%) |  |  | 0.279 | 0.597 |  |  |  | 0.000 | 1.000 |
| NO | 26(26.8) | 16(23.2) |  |  |  | 15(25.9) | 15(25.9) |  |  |
| YES | 71(73.2) | 53(76.8) |  |  |  | 43(74.1) | 43(74.1) |  |  |
| AMI, n(%) |  |  | 0.047 | 0.828 |  |  |  | - | - |
| NO | 17(17.5) | 13(18.8) |  |  |  | - | - |  |  |
| YES | 80(82.5) | 56(81.2) |  |  |  | - | - |  |  |
| Aspirin, n(%) | 83(85.6) | 63(91.3) | 1.252 | 0.263 |  | 46(79.3) | 52(89.7) | 2.367 | 0.124 |
| Clopidogrel, n(%) | 50(51.5) | 30(43.5) | 2.680 | 0.262 |  | 34(58.6) | 20(34.5) | 6.791 | 0.009 |
| Ticagrelor, n(%) | 18(18.6) | 16(23.2) | 0.531 | 0.466 |  | 7(12.1) | 16(27.6) | 4.393 | 0.036 |
| Statins, n(%) | 92(94.8) | 65(94.2) | 0.032 | 0.857 |  | 55(94.8) | 55(94.8) | 0.000 | 1.000 |
| ARNI, ACEI or ARB, n(%) | 63(64.9) | 50(72.5) | 1.048 | 0.306 |  | 40(69) | 41(70.7) | 0.041 | 0.840 |
| β-Blockers, n(%) | 60(61.9) | 42(60.9) | 0.017 | 0.898 |  | 37(63.8) | 38(65.5) | 0.038 | 0.846 |
| CCBs, n(%) | 47(48.5) | 26(37.3) | 1.899 | 1.168 |  | 29(50) | 21(36.2) | 2.250 | 0.134 |
| Nitrates, n(%) | 35(36.1) | 26(37.7) | 0.044 | 0.833 |  | 22(37.9) | 22(37.9) | 0.000 | 1.000 |
| MRAs, n(%) | 3(3.1) | 9(13) | 5.953 | 0.015 |  | 0 | 8(13.8) | 8.593 | 0.003 |
| FBG (mmol/L), mean(SD) | 7.243±2.331 | 7.833±3.014 | 1.362 | 0.176 |  | 7.583±2.681 | 7.538±2.844 | -0.087 | 0.931 |
| Cr (μmol/L), mean(SD) | 80.835±85.534 | 78.820±23.204 | -0.191 | 0.849 |  | 87.810±109.523 | 79.648±23.475 | -0.555 | 0.580 |
| Lp(a) (mmol/L), mean(SD) | 22.048±26.605 | 26.742±27.824 | 1.099 | 0.273 |  | 26.143±30.258 | 26.605±28.470 | 0.085 | 0.933 |
| TG (mmol/L), mean(SD) | 1.786±1.467 | 1.907±2.629 | 0.378 | 0.706 |  | 1.897±1.546 | 1.576±1.165 | -1.263 | 0.209 |
| TC (mmol/L), mean(SD) | 3.938±1.161 | 3.797±1.259 | -0.744 | 0.458 |  | 3.779±1.121 | 3.791±1.175 | 0.057 | 0.955 |
| LDL (mmol/L), mean(SD) | 2.146±1.035 | 1.980±0.957 | -1.052 | 0.294 |  | 1.973±0.998 | 2.075±0.986 | 0.551 | 0.582 |
| HDL (mmol/L), mean(SD) | 1.166±0.323 | 1.157±0.306 | -0.178 | 0.859 |  | 1.147±0.346 | 1.156±0.318 | 0.137 | 0.891 |
| HbA1c (%), mean(SD) | 7.078±1.098 | 7.491±1.252 | 2.252 | 0.026 |  | 7.247±1.083 | 7.310±1.119 | 0.312 | 0.756 |
| MBA, mean(SD) | 62.894±8.904 | 59.808±12.196 | -1.789 | 0.076 |  | 61.451±9.674 | 59.108±11.712 | -1.175 | 0.243 |
| FD-RVN (×10), mean(SD) | 1.484±0.068 | 1.448±0.073 | -3.236 | 0.001 |  | 1.476±0.073 | 1.448±0.073 | -2.067 | 0.041 |
| MRVC(Caliber) (μm), mean(SD) | 70.178±5.628 | 79.888±10.454 | 7.025 | 0.000 |  | 70.456±5.595 | 79.077±9.649 | 5.887 | 0.000 |
| MRVC(Caliber)<sub>0.5–1.0PD</sub> | 84.679±9.670 | 94.602±15.624 | 4.677 | 0.000 |  | 85.812±9.023 | 92.414±11.116 | 3.512 | 0.001 |
| MRVC(Caliber)<sub>1.0–1.5PD</sub> | 84.978±8.838 | 92.883±12.251 | 4.579 | 0.000 |  | 85.068±8.426 | 91.574±10.936 | 3.589 | 0.000 |
| MRVC(Caliber)<sub>1.5–2.0PD</sub> | 80.949±7.552 | 88.449±12.592 | 4.414 | 0.000 |  | 81.123±8.091 | 87.378±10.663 | 3.559 | 0.001 |
| MRVC(Caliber)<sub>2.0–2.5PD</sub> | 75.819±7.510 | 84.390±13.050 | 4.908 | 0.000 |  | 75.927±8.057 | 83.207±12.615 | 3.704 | 0.000 |
| MRV(Vessel)T (×10^4^) , mean(SD) | 0.0008±0.0001 | 0.0008±0.0002 | -0.512 | 0.610 |  | 0.0008±0.0001 | 0.0008±0.0002 | -0.089 | 0.929 |
| MRVκ<sub>0.5–1.0PD</sub> | 0.0007±0.0002 | 0.0007±0.0002 | 0.130 | 0.897 |  | 0.0007±0.0002 | 0.0007±0.0002 | 0.906 | 0.367 |
| MRVκ<sub>1.0–1.5PD</sub> | 0.0007±0.0002 | 0.0007±0.0003 | 0.851 | 0.397 |  | 0.0007±0.0002 | 0.0007±0.0003 | 0.766 | 0.445 |
| MRVκ<sub>1.5–2.0PD</sub> | 0.0008±0.0002 | 0.0007±0.0003 | -1.224 | 0.223 |  | 0.0008±0.0002 | 0.0007±0.0003 | -0.593 | 0.554 |
| MRVκ<sub>2.0–2.5PD</sub> | 0.0008±0.0002 | 0.0008±0.0003 | -0.731 | 0.466 |  | 0.0008±0.0002 | 0.0008±0.0003 | -0.049 | 0.961 |
| MRAT (×10^4^), mean(SD) | 0.0006±0.0001 | 0.0006±0.0002 | 1.095 | 0.276 |  | 0.0006±0.0001 | 0.0006±0.0002 | 1.411 | 0.161 |
| MRAC(Curvature)<sub>0.5-1.0PD</sub> | 0.0006±0.0002 | 0.0006±0.0003 | 0.014 | 0.989 |  | 0.0006±0.0002 | 0.0006±0.0003 | 1.508 | 0.135 |
| MRAC(Curvature)<sub>1.0-1.5PD</sub> | 0.0006±0.0002 | 0.0006±0.0003 | 1.670 | 0.098 |  | 0.0005±0.0002 | 0.0006±0.0003 | 1.817 | 0.072 |
| MRAC(Curvature)<sub>1.5-2.0PD</sub> | 0.0006±0.0003 | 0.0006±0.0003 | 0.746 | 0.456 |  | 0.0006±0.0002 | 0.0006±0.0003 | 1.171 | 0.244 |
| MRAC(Curvature)<sub>2.0-2.5PD</sub> | 0.0006±0.0002 | 0.0006±0.0002 | 0.492 | 0.624 |  | 0.0006±0.0002 | 0.0006±0.0002 | 0.707 | 0.481 |
| MRV(Venular)T (×10^4^), mean(SD) | 0.0009±0.0002 | 0.0008±0.0002 | -1.610 | 0.110 |  | 0.0009±0.0002 | 0.0008±0.0003 | -1.047 | 0.298 |
| MRVC(Curvature)<sub>0.5-1.0PD</sub> | 0.0008±0.0002 | 0.0008±0.0003 | -0.287 | 0.774 |  | 0.0008±0.0002 | 0.0008±0.0003 | 0.316 | 0.753 |
| MRVC(Curvature)<sub>1.0-1.5PD</sub> | 0.0008±0.0003 | 0.0008±0.0003 | -0.239 | 0.811 |  | 0.0008±0.0003 | 0.0008±0.0003 | -0.375 | 0.708 |
| MRVC(Curvature)<sub>1.5-2.0PD</sub> | 0.0009±0.0003 | 0.0008±0.0003 | -2.171 | 0.031 |  | 0.0009±0.0003 | 0.0008±0.0003 | -1.750 | 0.083 |
| MRVC(Curvature)<sub>2.0-2.5PD</sub> | 0.0009±0.0003 | 0.0009±0.0003 | -1.669 | 0.097 |  | 0.0009±0.0003 | 0.0008±0.0003 | -0.959 | 0.340 |
| VD (×10^2^), mean(SD) | 0.064±0.019 | 0.055±0.020 | -3.149 | 0.002 |  | 0.062±0.020 | 0.054±0.019 | -2.209 | 0.029 |
| MRAC (μm) , mean(SD) | 56.944±5.533 | 59.114±8.081 | 1.932 | 0.056 |  | 56.575±5.295 | 59.356±8.186 | 2.173 | 0.032 |
| MRAC(Caliber)<sub>0.5-1.0PD</sub> | 62.853±8.991 | 64.324±9.412 | 1.018 | 0.310 |  | 63.817±8.943 | 64.390±9.814 | 0.329 | 0.743 |
| MRAC(Caliber)<sub>1.0-1.5PD</sub> | 62.590±8.490 | 63.465±10.609 | 0.590 | 0.556 |  | 61.507±8.561 | 63.122±9.845 | 0.943 | 0.348 |
| MRAC(Caliber)<sub>1.5-2.0PD</sub> | 61.799±8.605 | 62.665±10.640 | 0.579 | 0.564 |  | 61.107±7.796 | 62.665±10.837 | 0.889 | 0.376 |
| MRAC(Caliber)<sub>2.0-2.5PD</sub> | 59.510±8.317 | 61.092±12.516 | 0.979 | 0.329 |  | 58.649±6.692 | 61.222±12.936 | 1.346 | 0.182 |
| MRV(Venular)C (μm), mean(SD) | 80.303±5.067 | 92.900±10.211 | 9.453 | 0.000 |  | 80.315±5.118 | 91.996±8.938 | 8.637 | 0.000 |
| MRV(Venular)C<sub>0.5-1.0PD</sub> | 102.761±11.949 | 113.297±16.506 | 4.525 | 0.000 |  | 104.295±12.285 | 111.435±14.070 | 2.911 | 0.004 |
| MRV(Venular)C<sub>1.0-1.5PD</sub> | 100.595±10.197 | 110.583±12.645 | 5.624 | 0.000 |  | 101.378±10.298 | 109.845±10.843 | 4.312 | 0.000 |
| MRV(Venular)C<sub>1.5-2.0PD</sub> | 93.808±8.986 | 104.489±12.576 | 6.385 | 0.000 |  | 94.974±9.815 | 102.416±9.795 | 4.087 | 0.000 |
| MRV(Venular)C<sub>2.0-2.5PD</sub> | 86.998±10.296 | 96.114±15.216 | 4.322 | 0.000 |  | 87.570±11.155 | 95.001±15.062 | 3.020 | 0.003 |
| AVR , mean(SD) | 0.710±0.067 | 0.640±0.083 | -6.048 | 0.000 |  | 0.706±0.064 | 0.647±0.076 | -4.514 | 0.000 |
| AVR <sub>0.5-1.0PD</sub> | 0.614±0.091 | 0.534±0.182 | -3.341 | 0.001 |  | 0.614±0.099 | 0.556±0.149 | -2.468 | 0.015 |
| AVR <sub>1.0-1.5PD</sub> | 0.627±0.094 | 0.560±0.141 | -3.660 | 0.000 |  | 0.612±0.101 | 0.556±0.140 | -2.480 | 0.015 |
| AVR <sub>1.5-2.0PD</sub> | 0.661±0.088 | 0.585±0.146 | -3.845 | 0.000 |  | 0.646±0.078 | 0.591±0.151 | -2.461 | 0.015 |
| AVR <sub>2.0-2.5PD</sub> | 0.691±0.111 | 0.632±0.142 | -2.972 | 0.003 |  | 0.679±0.105 | 0.637±0.142 | -1.811 | 0.073 |
| DA (μm^2^), mean(SD) | 961125.912±1087209.774 | 1402111.890±1438431.779 | 2.147 | 0.034 |  | 1033903.538±1064081.762 | 1440253.470±1364311.344 | 1.789 | 0.076 |
| HDD (μm), mean(SD) | 1548.208±251.870 | 1677.762±295.253 | 3.039 | 0.003 |  | 1542.325±213.129 | 1690.804±260.653 | 3.358 | 0.001 |
| VDD (μm), mean(SD) | 1712.523±272.567 | 1791.130±259.744 | 1.867 | 0.064 |  | 1712.006±217.633 | 1785.645±238.609 | 1.737 | 0.085 |
| ODR, mean(SD) | 0.835±0.085 | 0.844±0.070 | 0.732 | 0.465 |  | 0.833±0.097 | 0.851±0.051 | 1.239 | 0.218 |
| ODT (D), mean(SD) | -25.878±67.634 | -21.760±63.375 | 0.397 | 0.692 |  | -21.486±68.681 | -23.724±61.587 | -0.185 | 0.854 |
| MRW (μm), mean(SD) | 2680.049±625.293 | 2906.005±708.142 | 2.171 | 0.031 |  | 2695.623±638.082 | 2985.757±680.831 | 2.368 | 0.020 |
| DDM (μm), mean(SD) | 4313.041±308.188 | 4316.870±315.847 | 0.078 | 0.938 |  | 4265.541±293.053 | 4318.173±305.477 | 0.947 | 0.346 |
| ODAR, mean(SD) | 1.170±0.159 | 1.155±0.128 | -0.645 | 0.520 |  | 1.185±0.195 | 1.142±0.076 | -1.544 | 0.127 |
| MFD (μm), mean(SD) | 4816.586±307.840 | 4352.350±1298.400 | -2.912 | 0.005 |  | 4863.797±184.152 | 4296.483±1401.943 | -3.056 | 0.003 |
| OCA (μm^2^), mean(SD) | 223454.169±307035.342 | 386595.656±479096.778 | 2.488 | 0.014 |  | 231071.851±286865.959 | 386389.571±441350.221 | 2.247 | 0.027 |
| CHD (μm), mean(SD) | 777.041±211.340 | 863.247±270.732 | 2.302 | 0.023 |  | 751.606±210.102 | 858.425±258.191 | 2.444 | 0.016 |
| CVD (μm), mean(SD) | 790.377±219.229 | 858.811±224.461 | 1.963 | 0.051 |  | 757.694±213.065 | 849.152±212.770 | 2.313 | 0.023 |
| CIR-C, mean(SD) | 0.864±0.068 | 0.850±0.064 | -1.353 | 0.178 |  | 0.861±0.079 | 0.851±0.061 | -0.767 | 0.445 |
| CDR-A, mean(SD) | 0.233±0.087 | 0.246±0.089 | 0.961 | 0.338 |  | 0.221±0.082 | 0.244±0.087 | 1.501 | 0.136 |
| HCDR, mean(SD) | 0.494±0.105 | 0.503±0.097 | 0.594 | 0.553 |  | 0.484±0.104 | 0.500±0.093 | 0.887 | 0.377 |
| VCDR, mean(SD) | 0.452±0.087 | 0.472±0.084 | 1.468 | 0.144 |  | 0.438±0.087 | 0.471±0.083 | 2.126 | 0.036 |
| Rim-I (μm), mean(SD) | 451.634±76.448 | 453.912±95.034 | 0.171 | 0.864 |  | 447.030±78.951 | 452.657±95.513 | 0.346 | 0.730 |
| Rim-N (μm), mean(SD) | 429.914±113.660 | 437.351±106.833 | 0.426 | 0.671 |  | 417.133±115.273 | 437.915±108.345 | 1.000 | 0.319 |
| Rim-S (μm), mean(SD) | 483.339±85.106 | 497.767±89.068 | 1.056 | 0.293 |  | 495.241±84.588 | 500.630±91.069 | 0.330 | 0.742 |
| Rim-T (μm), mean(SD) | 347.006±104.596 | 381.456±98.766 | 2.140 | 0.034 |  | 361.262±102.650 | 391.621±102.600 | 1.593 | 0.114 |
| Max Rim-Cup D (μm), mean(SD) | 1354.832±198.053 | 1446.759±247.809 | 2.653 | 0.009 |  | 1328.411±204.813 | 1440.988±244.731 | 2.687 | 0.008 |
| Min Rim (μm), mean(SD) | 294.765±82.287 | 311.940±70.552 | 1.405 | 0.162 |  | 302.112±79.396 | 312.095±70.858 | 0.714 | 0.476 |

MBA, Mean Branching Angle; FD-RVN,Fractal Dimension of the Retinal Vascular Network; MRVC(Caliber) , Mean Retinal Vessel Caliber; MRVC(Caliber)<sub>0.5-1.0PD</sub>, Mean Retinal Vessel Caliber within 0.5-1.0 PD Zone; MRV(Vessel)T, Mean Retinal Vessel Tortuosity; MRVκ<sub>0.5-1.0PD</sub>, Mean Retinal Vessel Curvature within 0.5-1.0 PD Zone; MRAT, Mean Retinal Arteriolar Tortuosity; MRAC(Curvature)<sub>0.5-1.0PD</sub>, Mean Retinal Arteriolar Curvature within 0.5-1.0 PD Zone; MRV(Venular)T, Mean Retinal Venular Tortuosity; MRVC(Curvature)<sub>0.5-1.0PD</sub> ,Mean Retinal Venular Curvature within 0.5-1.0 PD Zone; VD, Vessel Density; MRAC, Mean Retinal Arteriolar Caliber ; MRAC(Caliber)<sub>0.5-1.0PD</sub> , Mean Retinal Arteriolar Caliber within 0.5-1.0 PD Zone; MRV(Venular)C, Mean Retinal Venular Caliber ; MRV(Venular)C<sub>0.5-1.0PD</sub>, Mean Retinal Venular Caliber within 0.5-1.0 PD Zone; AVR, Arteriolar-to-Venular Ratio; DA, Optic Disc Area; HDD, Horizontal Diameter of the Optic Disc; VDD, Vertical Optic Disc Diameter; ODR, Optic Disc Roundness; ODT (D), Optic Disc Tilt; MRW, Minimum Rim Width; DDM, Disc-Macula Distance; ODAR, Optic Disc Axial Ratio; MFD, Macular Fovea Distance; OCA, Optic Cup Area; CHD, Cup Horizontal Diameter; CVD, Cup Vertical Diameter; CIR-C, Cup Circularity; CDR-A, Cup-to-Disc Area Ratio; HCDR, Horizontal Cup-to-Disc Ratio; VCDR, Vertical Cup-to-Disc Ratio; Rim-I, Inferior Neuroretinal Rim Width; Rim-N, Nasal Neuroretinal Rim Width; Rim-S, Superior Neuroretinal Rim Width; Rim-T, Temporal Neuroretinal Rim Width; Max Rim-Cup D, Maximum Rim-to-Cup Distance; Min Rim, Minimum Neuroretinal Rim Width; Data are mean±SD or n (%). Continuous and categorical variables were compared using the independent samples t-test/Mann-Whitney U test and Chi-squared/Fisher's exact test, respectively. P < 0.05 was considered significant.

Supplemental Table 6. Clinical and demographic characteristics of CHD patients with T2DM stratified by short-term SGLT2 inhibitor use versus non-use.

|  | **Primary cohort** | | | |
| --- | --- | --- | --- | --- |
|  | **SGLT2i(N)** | **SGLT2i(Y)** | **B** | **P value** |
| N | 28 | 27 |  |  |
| Age (years), mean(SD) | 60.290±10.780 | 58.850±10.328 | 0.503 | 0.617 |
| Sex |  |  | 0.439 | 0.508 |
| Male, n(%) | 17(60.7) | 14(51.9) |  |  |
| Female, n(%) | 11(39.3) | 13(48.1) |  |  |
| Smoking history, n(%) |  |  | 0.439 | 0.508 |
| No smoking | 19(67.9) | 16(59.3) |  |  |
| Still smoking | 9(32.1) | 11(40.7) |  |  |
| Drinking history, n(%) |  |  | 0.093 | **0.760** |
| No drinking | 23(82.1) | 23(85.2) |  |  |
| Still drinking | 5(17.9) | 4(14.8) |  |  |
| CHD, n(%) |  |  | 0.439 | 0.508 |
| NO | 9(32.1) | 11(40.7) |  |  |
| YES | 19(67.9) | 16(59.3) |  |  |
| AMI, n(%) |  |  | 0.164 | 0.686 |
| NO | 5(17.9) | 6(22.2) |  |  |
| YES | 23(82.1) | 21(77.8) |  |  |
| Aspirin, n(%) | 22(78.6) | 23(85.2) | 0.404 | 0.525 |
| Clopidogrel, n(%) | 14(50) | 12(44.4) | 0.170 | 0.680 |
| Ticagrelor, n(%) | 5(17.9) | 4(14.8) | 0.093 | 0.760 |
| Statins, n(%) | 27(96.4) | 26(96.3) | 0.001 | 0.979 |
| ARNI, ACEI or ARB, n(%) | 3(10.7) | 3(11.1) | 0.002 | 0.962 |
| β-Blockers, n(%) | 16(57.1) | 12(44.4) | 0.887 | 0.346 |
| CCBs, n(%) | 0 | 0 | 0 | 0 |
| Nitrates, n(%) | 6(21.4) | 10(37) | 1.623 | 0.203 |
| MRAs, n(%) | 0 | 2(7.4) | 2.152 | 0.142 |
| FBG (mmol/L), mean(SD) | 8.321±3.210 | 7.207±1.864 | 1.566 | 0.123 |
| Cr (μmol/L), mean(SD) | 67.820±19.760 | 67.890±18.415 | -0.013 | 0.990 |
| Lp(a) (mmol/L), mean(SD) | 24.329±27.512 | 28.956±25.443 | -0.647 | 0.520 |
| TG (mmol/L), mean(SD) | 1.606±1.557 | 1.568±1.039 | 0.105 | 0.917 |
| TC (mmol/L), mean(SD) | 3.947±1.262 | 4.036±1.253 | -0.263 | 0.794 |
| LDL (mmol/L), mean(SD) | 2.135±0.967 | 2.346±1.092 | -0.759 | 0.451 |
| HDL (mmol/L), mean(SD) | 1.275±0.428 | 1.156±0.231 | 1.295 | 0.202 |
| HbA1c (%), mean(SD) | 7.596±1.066 | 7.500±0.947 | 0.354 | 0.725 |
| MBA, mean(SD) | 55.968±10.387 | 59.371±13.365 | -1.056 | 0.296 |
| FD-RVN (×10), mean(SD) | 1.460±0.064 | 1.458±0.068 | 0.092 | 0.927 |
| MRVC(Caliber) (μm), mean(SD) | 88.782±9.107 | 77.722±8.231 | 4.720 | 0.000 |
| MRVC(Caliber)<sub>0.5–1.0PD</sub> | 100.713±7.094 | 90.532±10.180 | 4.316 | 0.000 |
| MRVC(Caliber)<sub>1.0–1.5PD</sub> | 98.206±9.208 | 90.914±9.055 | 2.960 | 0.005 |
| MRVC(Caliber)<sub>1.5–2.0PD</sub> | 90.784±9.674 | 87.672±9.307 | 1.215 | 0.230 |
| MRVC(Caliber)<sub>2.0–2.5PD</sub> | 84.586±11.257 | 83.195±8.170 | 0.523 | 0.603 |
| MRV(Vessel)T (×10^4^) , mean(SD) | 0.0006±0.0001 | 0.0007±0.0001 | -3.468 | 0.001 |
| MRVκ<sub>0.5–1.0PD</sub> | 0.0006±0.0001 | 0.0007±0.0002 | -2.298 | 0.026 |
| MRVκ<sub>1.0–1.5PD</sub> | 0.0006±0.0002 | 0.0007±0.0002 | -1.810 | 0.076 |
| MRVκ<sub>1.5–2.0PD</sub> | 0.0006±0.0002 | 0.0007±0.0002 | -2.278 | 0.027 |
| MRVκ<sub>2.0–2.5PD</sub> | 0.0006±0.0002 | 0.0007±0.0002 | -1.612 | 0.113 |
| MRAT (×10^4^), mean(SD) | 0.0006±0.0002 | 0.0006±0.0001 | -1.005 | 0.320 |
| MRAC(Curvature)<sub>0.5-1.0PD</sub> | 0.0006±0.0002 | 0.0006±0.0002 | -0.049 | 0.961 |
| MRAC(Curvature)<sub>1.0-1.5PD</sub> | 0.0006±0.0003 | 0.0006±0.0002 | -0.291 | 0.772 |
| MRAC(Curvature)<sub>1.5-2.0PD</sub> | 0.0005±0.0002 | 0.0005±0.0003 | 0.337 | 0.737 |
| MRAC(Curvature)<sub>2.0-2.5PD</sub> | 0.0006±0.0004 | 0.0006±0.0002 | 0.277 | 0.783 |
| MRV(Venular)T (×10^4^), mean(SD) | 0.0007±0.0002 | 0.0008±0.0002 | -3.661 | 0.001 |
| MRVC(Curvature)<sub>0.5-1.0PD</sub> | 0.0007±0.0002 | 0.0008±0.0003 | -2.287 | 0.026 |
| MRVC(Curvature)<sub>1.0-1.5PD</sub> | 0.0006±0.0002 | 0.0007±0.0003 | -2.064 | 0.045 |
| MRVC(Curvature)<sub>1.5-2.0PD</sub> | 0.0007±0.0002 | 0.0008±0.0003 | -2.323 | 0.025 |
| MRVC(Curvature)<sub>2.0-2.5PD</sub> | 0.0006±0.0002 | 0.0007±0.0002 | -1.574 | 0.121 |
| VD (×10^2^), mean(SD) | 0.057±0.020 | 0.055±0.017 | 0.363 | 0.718 |
| MRAC (μm) , mean(SD) | 72.244±6.426 | 61.510±5.472 | 6.658 | 0.000 |
| MRAC(Caliber)<sub>0.5-1.0PD</sub> | 76.749±6.967 | 64.818±10.032 | 5.138 | 0.000 |
| MRAC(Caliber)<sub>1.0-1.5PD</sub> | 76.502±9.122 | 67.709±9.071 | 3.584 | 0.001 |
| MRAC(Caliber)<sub>1.5-2.0PD</sub> | 72.643±8.222 | 65.571±7.898 | 3.252 | 0.002 |
| MRAC(Caliber)<sub>2.0-2.5PD</sub> | 67.588±8.428 | 62.763±8.050 | 2.170 | 0.035 |
| MRV(Venular)C (μm), mean(SD) | 102.320±12.382 | 91.393±9.600 | 3.648 | 0.001 |
| MRV(Venular)C<sub>0.5-1.0PD</sub> | 127.497±14.256 | 109.003±14.261 | 4.809 | 0.000 |
| MRV(Venular)C<sub>1.0-1.5PD</sub> | 115.920±11.250 | 108.866±11.935 | 2.256 | 0.028 |
| MRV(Venular)C<sub>1.5-2.0PD</sub> | 104.297±11.155 | 103.327±11.363 | 0.319 | 0.751 |
| MRV(Venular)C<sub>2.0-2.5PD</sub> | 97.132±15.400 | 101.026±12.917 | -1.014 | 0.315 |
| AVR , mean(SD) | 0.712±0.073 | 0.676±0.055 | 2.059 | 0.044 |
| AVR <sub>0.5-1.0PD</sub> | 0.609±0.087 | 0.598±0.115 | 0.432 | 0.667 |
| AVR <sub>1.0-1.5PD</sub> | 0.647±0.152 | 0.623±0.089 | 0.705 | 0.484 |
| AVR <sub>1.5-2.0PD</sub> | 0.700±0.078 | 0.641±0.085 | 2.691 | 0.010 |
| AVR <sub>2.0-2.5PD</sub> | 0.704±0.087 | 0.634±0.100 | 2.750 | 0.008 |
| DA (μm^2^), mean(SD) | 2542065.931±1566258.504 | 913666.873±1382240.611 | 4.082 | 0.000 |
| HDD (μm), mean(SD) | 1883.510±288.294 | 1613.444±246.731 | 3.726 | 0.000 |
| VDD (μm), mean(SD) | 2036.728±325.686 | 1763.481±257.324 | 3.444 | 0.001 |
| ODR, mean(SD) | 0.847±0.046 | 0.839±0.039 | 0.698 | 0.488 |
| ODT (D), mean(SD) | -17.320±69.395 | -32.451±69.338 | 0.809 | 0.422 |
| MRW (μm), mean(SD) | 2873.490±709.617 | 2994.579±451.937 | -0.752 | 0.456 |
| DDM (μm), mean(SD) | 4673.286±509.569 | 4316.184±346.629 | 3.048 | 0.004 |
| ODAR, mean(SD) | 1.149±0.072 | 1.140±0.061 | 0.535 | 0.595 |
| MFD (μm), mean(SD) | 3912.279±1501.450 | 4551.399±678.138 | -2.046 | 0.048 |
| OCA (μm^2^), mean(SD) | 686846.427±580734.098 | 1236374.194±7762225.497 | 1.284 | 0.210 |
| CHD (μm), mean(SD) | 952.539±299.545 | -2040.440±14570.214 | 1.067 | 0.296 |
| CVD (μm), mean(SD) | 970.409±318.640 | -1872.861±13805.127 | 1.070 | 0.294 |
| CIR-C, mean(SD) | 0.854±0.060 | 0.373±2.562 | 0.977 | 0.338 |
| CDR-A, mean(SD) | 0.245±0.099 | -0.976±6.165 | 1.028 | 0.313 |
| HCDR, mean(SD) | 0.497±0.108 | -0.781±6.465 | 1.027 | 0.314 |
| VCDR, mean(SD) | 0.466±0.098 | -0.992±7.419 | 1.021 | 0.317 |
| Rim-I (μm), mean(SD) | 537.660±89.835 | 487.198±99.319 | 1.978 | 0.053 |
| Rim-N (μm), mean(SD) | 481.530±111.892 | 470.885±102.325 | 0.368 | 0.714 |
| Rim-S (μm), mean(SD) | 536.333±92.931 | 500.897±98.360 | 1.374 | 0.175 |
| Rim-T (μm), mean(SD) | 443.296±135.746 | 372.173±111.074 | 2.122 | 0.039 |
| Max Rim-Cup D (μm), mean(SD) | 1622.216±317.627 | -192.174±8184.988 | 1.172 | 0.246 |
| Min Rim (μm), mean(SD) | 363.827±77.356 | 1134.472±4272.842 | -0.937 | 0.357 |

MBA, Mean Branching Angle; FD-RVN,Fractal Dimension of the Retinal Vascular Network; MRVC(Caliber) , Mean Retinal Vessel Caliber; MRVC(Caliber)<sub>0.5-1.0PD</sub>, Mean Retinal Vessel Caliber within 0.5-1.0 PD Zone; MRV(Vessel)T, Mean Retinal Vessel Tortuosity; MRVκ<sub>0.5-1.0PD</sub>, Mean Retinal Vessel Curvature within 0.5-1.0 PD Zone; MRAT, Mean Retinal Arteriolar Tortuosity; MRAC(Curvature)<sub>0.5-1.0PD</sub>, Mean Retinal Arteriolar Curvature within 0.5-1.0 PD Zone; MRV(Venular)T, Mean Retinal Venular Tortuosity; MRVC(Curvature)<sub>0.5-1.0PD</sub> ,Mean Retinal Venular Curvature within 0.5-1.0 PD Zone; VD, Vessel Density; MRAC, Mean Retinal Arteriolar Caliber ; MRAC(Caliber)<sub>0.5-1.0PD</sub> , Mean Retinal Arteriolar Caliber within 0.5-1.0 PD Zone; MRV(Venular)C, Mean Retinal Venular Caliber; MRV(Venular)C<sub>0.5-1.0PD</sub>, Mean Retinal Venular Caliber within 0.5-1.0 PD Zone; AVR, Arteriolar-to-Venular Ratio; DA, Optic Disc Area; HDD, Horizontal Diameter of the Optic Disc; VDD, Vertical Optic Disc Diameter; ODR, Optic Disc Roundness; ODT (D), Optic Disc Tilt; MRW, Minimum Rim Width; DDM, Disc-Macula Distance; ODAR, Optic Disc Axial Ratio; MFD, Macular Fovea Distance; OCA, Optic Cup Area; CHD, Cup Horizontal Diameter; CVD, Cup Vertical Diameter; CIR-C, Cup Circularity; CDR-A, Cup-to-Disc Area Ratio; HCDR, Horizontal Cup-to-Disc Ratio; VCDR, Vertical Cup-to-Disc Ratio; Rim-I, Inferior Neuroretinal Rim Width; Rim-N, Nasal Neuroretinal Rim Width; Rim-S, Superior Neuroretinal Rim Width; Rim-T, Temporal Neuroretinal Rim Width; Max Rim-Cup D, Maximum Rim-to-Cup Distance; Min Rim, Minimum Neuroretinal Rim Width; Data are mean±SD or n (%). Continuous and categorical variables were compared using the independent samples t-test/Mann-Whitney U test and Chi-squared/Fisher's exact test, respectively. P < 0.05 was considered significant.

Supplemental Table 7. Univariable logistic regression analysis of the primary cohort for the matched control group and the CHD group.

|  | B | SE | Wald | *P* | OR | 95％*CI* |
| --- | --- | --- | --- | --- | --- | --- |
| Age |  |  |  |  |  |  |
| Sex | 0.013 | 0.016 | 0.673 | 0.412 | 1.013 | 0.982-1.045 |
| Male | 1.727 | 0.392 | 19.383 | - | 5.625 | 2.607-12.136 |
| Female | Ref. |  |  |  |  |  |
| Smoking history |  |  |  |  |  |  |
| No smoking | Ref. |  |  |  |  |  |
| Still smoking | 1.366 | 0.384 | 12.629 | - | 3.918 | 1.845-8.20 |
| Drinking history |  |  |  |  |  |  |
| No drinking | Ref. |  |  |  |  |  |
| Still drinking | 1.004 | 0.513 | 3.827 | 0.05 | 2.728 | 0.998-7.458 |
| FBG | 0.091 | 0.076 | 1.448 | 0.229 | 1.095 | 0.944-1.271 |
| Cr | 0.007 | 0.008 | 0.722 | 0.395 | 1.007 | 0.991-1.024 |
| Lp(a) | 0.001 | 0.006 | 0.032 | 0.858 | 1.001 | 0.989-1.014 |
| TG | -0.065 | 0.131 | 0.248 | 0.619 | 0.937 | 0.725-1.211 |
| TC | 0.471 | 0.161 | 8.526 | 0.004 | 1.601 | 1.167-2.196 |
| LDL | 0.538 | 0.187 | 8.293 | 0.004 | 1.712 | 1.187-2.469 |
| HDL | 0.575 | 0.577 | 0.991 | 0.319 | 1.777 | 0.573-5.507 |
| HbA1c | 0.032 | 0.17 | 0.037 | 0.848 | 1.033 | 0.741-1.440 |
| MBA | -0.05 | 0.017 | 8.208 | 0.004 | 0.951 | 0.92-.0984 |
| FD-RVN | -8.287 | 3.922 | 4.465 | 0.035 | 0.000 | 0-0.549 |
| MRVC(Caliber) | 0.245 | 0.045 | 29.131 | <0.001 | 1.278 | 1.169-1.396 |
| MRVC(Caliber)<sub>0.5–1.0PD</sub> | 0.036 | 0.017 | 4.438 | 0.035 | 1.037 | 1.003-1.072 |
| MRVC(Caliber)<sub>1.0–1.5PD</sub> | 0.051 | 0.021 | 5.668 | 0.017 | 1.052 | 1.009-1.097 |
| MRVC(Caliber)<sub>1.5–2.0PD</sub> | 0.071 | 0.022 | 10.596 | 0.001 | 1.074 | 1.029-1.121 |
| MRVC(Caliber)<sub>2.0–2.5PD</sub> | 0.063 | 0.02 | 9.606 | 0.002 | 1.065 | 1.024-1.109 |
| MRV(Vessel)T | -2508.981 | 1330.714 | 3.555 | 0.059 | 0.000 | 0->100 |
| MRVκ<sub>0.5–1.0PD</sub> | -670.413 | 889.921 | 0.568 | 0.451 | 0.000 | - |
| MRVκ<sub>1.0–1.5PD</sub> | -362.119 | 692.288 | 0.274 | 0.601 | 0.000 | - |
| MRVκ<sub>1.5–2.0PD</sub> | -1500.338 | 802.933 | 3.492 | 0.062 | 0.000 | 0->100 |
| MRVκ<sub>2.0–2.5PD</sub> | -535.631 | 723.235 | 0.548 | 0.459 | 0.000 | - |
| MRAT | -689.913 | 1221.159 | 0.319 | 0.572 | 0.000 | - |
| MRAC(Curvature)<sub>0.5-1.0PD</sub> | 323.959 | 748.296 | 0.187 | 0.665 | >100 | - |
| MRAC(Curvature)<sub>1.0-1.5PD</sub> | 380.285 | 779.977 | 0.238 | 0.626 | >100 | - |
| MRAC(Curvature)<sub>1.5-2.0PD</sub> | -779.753 | 784.561 | 0.988 | 0.32 | 0.000 | - |
| MRAC(Curvature)<sub>2.0-2.5PD</sub> | -731.222 | 834.055 | 0.769 | 0.381 | 0.000 | - |
| MRV(Venular)T | -1728.924 | 1017.583 | 2.887 | 0.089 | 0.000 | 0->100 |
| MRVC(Curvature)<sub>0.5-1.0PD</sub> | -710.627 | 765.335 | 0.862 | 0.353 | 0.000 | - |
| MRVC(Curvature)<sub>1.0-1.5PD</sub> | -206.445 | 525.713 | 0.154 | 0.695 | 0.000 | - |
| MRVC(Curvature)<sub>1.5-2.0PD</sub> | -1126.096 | 643.489 | 3.062 | 0.08 | 0.000 | 0->100 |
| MRVC(Curvature)<sub>2.0-2.5PD</sub> | -86.219 | 524.578 | 0.027 | 0.869 | 0.000 | - |
| VD | -22.834 | 12.37 | 3.407 | 0.065 | 0.000 | 0-4.101 |
| MRAC | 0.074 | 0.029 | 6.519 | 0.011 | 1.077 | 1.017-1.14 |
| MRAC(Caliber)<sub>0.5-1.0PD</sub> | 0.031 | 0.02 | 2.441 | 0.118 | 1.032 | 0.992-1.073 |
| MRAC(Caliber)<sub>1.0-1.5PD</sub> | 0.015 | 0.021 | 0.503 | 0.478 | 1.015 | 0.974-1.057 |
| MRAC(Caliber)<sub>1.5-2.0PD</sub> | 0 | 0.018 | 0 | 0.998 | 1.000 | 0.965-1.037 |
| MRAC(Caliber)<sub>2.0-2.5PD</sub> | -0.015 | 0.017 | 0.752 | 0.386 | 0.985 | 0.952-1.019 |
| MRV(Venular)C | 0.315 | 0.06 | 27.588 | <0.001 | 1.370 | 1.218-1.541 |
| MRV(Venular)C<sub>0.5-1.0PD</sub> | 0.058 | 0.016 | 13.25 | <0.001 | 1.059 | 1.027-1.093 |
| MRV(Venular)C<sub>1.0-1.5PD</sub> | 0.05 | 0.017 | 8.446 | 0.004 | 1.051 | 1.016-1.087 |
| MRV(Venular)C<sub>1.5-2.0PD</sub> | 0.078 | 0.019 | 16.804 | <0.001 | 1.081 | 1.041-1.122 |
| MRV(Venular)C<sub>2.0-2.5PD</sub> | 0.059 | 0.015 | 14.589 | <0.001 | 1.060 | 1.029-1.093 |
| AVR | -21.616 | 4.035 | 28.696 | <0.001 | 0.000 | - |
| AVR <sub>0.5-1.0PD</sub> | -4.035 | 2.29 | 3.104 | 0.078 | 0.018 | 0-1.574 |
| AVR <sub>1.0-1.5PD</sub> | -3.386 | 2.135 | 2.515 | 0.113 | 0.034 | 0.001-2.222 |
| AVR <sub>1.5-2.0PD</sub> | -5.838 | 1.819 | 10.303 | 0.001 | 0.003 | 0-0.103 |
| AVR <sub>2.0-2.5PD</sub> | -6.319 | 1.603 | 15.546 | <0.001 | 0.002 | 0-0.042 |
| DA | 0 | 0 | 2.334 | 0.127 | 1.000 | 1-1 |
| HDD | 0.002 | 0.001 | 4.447 | 0.035 | 1.002 | 1-1.003 |
| VDD | 0 | 0.001 | 0.212 | 0.645 | 1.000 | 0.999-1.002 |
| ODR | 6.759 | 2.619 | 6.662 | 0.01 | 862.117 | 5.086-146122.3 |
| ODT (D) | 0.003 | 0.002 | 1.388 | 0.239 | 1.003 | 0.998-1.007 |
| MRW | 0.001 | 0 | 19.882 | <0.001 | 1.001 | 1-1.001 |
| DDM | -0.001 | 0 | 4.18 | 0.041 | 0.999 | 0.998-1 |
| ODAR | -4.795 | 2.138 | 5.028 | 0.025 | 0.008 | 0-0.547 |
| MFD | -0.001 | 0 | 25.486 | <0.001 | 0.999 | 0.999-0.999 |
| OCA | 0 | 0 | 0.001 | 0.97 | 1.000 | 1-1 |
| CHD | 0.001 | 0.001 | 1.108 | 0.292 | 1.001 | 0.999-1.002 |
| CVD | 0 | 0.001 | 0.011 | 0.915 | 1.000 | 0.998-1.001 |
| CIR-C | 1.109 | 1.783 | 0.387 | 0.534 | 3.032 | 0.092-99.895 |
| CDR-A | -1.194 | 2.091 | 0.326 | 0.568 | 0.303 | 0.005-18.247 |
| HCDR | 0.486 | 1.762 | 0.076 | 0.783 | 1.626 | 0.051-51.416 |
| VCDR | -0.601 | 2.134 | 0.079 | 0.778 | 0.548 | 0.008-35.956 |
| Rim-I | 0.001 | 0.001 | 0.989 | 0.32 | 1.001 | 0.999-1.004 |
| Rim-N | 0.002 | 0.001 | 1.768 | 0.184 | 1.002 | 0.999-1.005 |
| Rim-S | 0.003 | 0.002 | 3.238 | 0.072 | 1.003 | 1-1.006 |
| Rim-T | 0.001 | 0.001 | 0.745 | 0.388 | 1.001 | 0.998-1.004 |
| Max Rim-Cup D | 0.001 | 0.001 | 0.996 | 0.318 | 1.001 | 0.999-1.003 |
| Min Rim | 0.001 | 0.002 | 0.319 | 0.572 | 1.001 | 0.998-1.004 |

MBA, Mean Branching Angle; FD-RVN,Fractal Dimension of the Retinal Vascular Network; MRVC(Caliber) , Mean Retinal Vessel Caliber; MRVC(Caliber)<sub>0.5-1.0PD</sub>, Mean Retinal Vessel Caliber within 0.5-1.0 PD Zone; MRV(Vessel)T, Mean Retinal Vessel Tortuosity; MRVκ<sub>0.5-1.0PD</sub>, Mean Retinal Vessel Curvature within 0.5-1.0 PD Zone; MRAT, Mean Retinal Arteriolar Tortuosity; MRAC(Curvature)<sub>0.5-1.0PD</sub>, Mean Retinal Arteriolar Curvature within 0.5-1.0 PD Zone; MRV(Venular)T, Mean Retinal Venular Tortuosity; MRVC(Curvature)<sub>0.5-1.0PD</sub> ,Mean Retinal Venular Curvature within 0.5-1.0 PD Zone; VD, Vessel Density; MRAC, Mean Retinal Arteriolar Caliber ; MRAC(Caliber)<sub>0.5-1.0PD</sub> , Mean Retinal Arteriolar Caliber within 0.5-1.0 PD Zone; MRV(Venular)C, Mean Retinal Venular Caliber ; MRV(Venular)C<sub>0.5-1.0PD</sub>, Mean Retinal Venular Caliber within 0.5-1.0 PD Zone; AVR, Arteriolar-to-Venular Ratio; DA, Optic Disc Area; HDD, Horizontal Diameter of the Optic Disc; VDD, Vertical Optic Disc Diameter; ODR, Optic Disc Roundness; ODT (D), Optic Disc Tilt; MRW, Minimum Rim Width; DDM, Disc-Macula Distance; ODAR, Optic Disc Axial Ratio; MFD, Macular Fovea Distance; OCA, Optic Cup Area; CHD, Cup Horizontal Diameter; CVD, Cup Vertical Diameter; CIR-C, Cup Circularity; CDR-A, Cup-to-Disc Area Ratio; HCDR, Horizontal Cup-to-Disc Ratio; VCDR, Vertical Cup-to-Disc Ratio; Rim-I, Inferior Neuroretinal Rim Width; Rim-N, Nasal Neuroretinal Rim Width; Rim-S, Superior Neuroretinal Rim Width; Rim-T, Temporal Neuroretinal Rim Width; Max Rim-Cup D, Maximum Rim-to-Cup Distance; Min Rim, Minimum Neuroretinal Rim Width; Results of univariate logistic regression are presented as odds ratios (OR) with 95% confidence intervals (CI). An OR > 1 indicates increased odds of the outcome, and an OR < 1 indicates decreased odds; P < 0.05 was considered significant.

Supplemental Table 8. Univariable logistic regression analysis of the primary cohort for the HTN group and the CHD with HTN group.

|  | B | SE | Wald | *P* | OR | 95％*CI* |
| --- | --- | --- | --- | --- | --- | --- |
| Age | 0.021 | 0.014 | 2.379 | 0.123 | 1.021 | 0.994-1.049 |
| Sex |  |  |  |  |  |  |
| Male | 0.868 | 0.314 | 7.620 | 0.006 | 2.382 | 1.286-4.413 |
| Female | Ref. |  |  |  |  |  |
| Smoking history |  |  |  |  |  |  |
| No smoking | Ref. |  |  |  |  |  |
| Still smoking | 0.474 | 0.310 | 2.340 | 0.126 | 1.606 | 0.875-2.948 |
| Drinking history |  |  |  |  |  |  |
| No drinking | Ref. |  |  |  |  |  |
| Still drinking | 0.534 | 0.382 | 1.958 | 0.162 | 1.706 | 0.807-3.604 |
| FBG | 0.029 | 0.167 | 0.029 | 0.864 | 1.029 | 0.742-1.428 |
| Cr | 0.022 | 0.009 | 5.592 | 0.018 | 1.022 | 1.004-1.040 |
| Lp(a) | 0.010 | 0.006 | 2.425 | 0.119 | 1.010 | 0.997-1.023 |
| TG | 0.146 | 0.170 | 0.740 | 0.390 | 1.158 | 0.829-1.616 |
| TC | -0.422 | 0.138 | 9.377 | 0.002 | 0.655 | 0.500-0.859 |
| LDL | -0.419 | 0.158 | 6.977 | 0.008 | 0.658 | 0.482-0.898 |
| HDL | -1.266 | 0.435 | 8.473 | 0.004 | 0.282 | 0.120-0.661 |
| HbA1c | 0.353 | 0.303 | 1.350 | 0.245 | 1.423 | 0.785-2.579 |
| MBA | 0.041 | 0.014 | 9.009 | 0.003 | 1.042 | 1.014-1.070 |
| FD-RVN | 3.479 | 2.420 | 2.066 | 0.151 | 32.426 | 0.282-3723.660 |
| MRVC(Caliber) | -0.204 | 0.030 | 46.954 | 0.000 | 0.815 | 0.769-0.864 |
| MRVC(Caliber)<sub>0.5–1.0PD</sub> | -0.096 | 0.018 | 28.516 | 0.000 | 0.909 | 0.877-0.941 |
| MRVC(Caliber)<sub>1.0–1.5PD</sub> | -0.088 | 0.017 | 25.733 | 0.000 | 0.916 | 0.885-0.948 |
| MRVC(Caliber)<sub>1.5–2.0PD</sub> | -0.126 | 0.023 | 29.954 | 0.000 | 0.881 | 0.842-0.922 |
| MRVC(Caliber)<sub>2.0–2.5PD</sub> | -0.121 | 0.023 | 27.950 | 0.000 | 0.886 | 0.847-0.927 |
| MRV(Vessel)T | 2697.059 | 981.169 | 7.556 | 0.006 | - | - |
| MRVκ<sub>0.5–1.0PD</sub> | 1924.440 | 789.042 | 5.949 | 0.015 | - | >100- |
| MRVκ<sub>1.0–1.5PD</sub> | 1050.313 | 755.205 | 1.934 | 0.164 | - | 0.000 |
| MRVκ<sub>1.5–2.0PD</sub> | 1839.074 | 705.188 | 6.801 | 0.009 | - | >100- |
| MRVκ<sub>2.0–2.5PD</sub> | 612.699 | 721.463 | 0.721 | 0.396 | >100 | 0.000 |
| MRAT | 1370.850 | 872.304 | 2.470 | 0.116 | - | 0.000 |
| MRAC(Curvature)<sub>0.5-1.0PD</sub> | 769.519 | 586.080 | 1.724 | 0.189 | - | 0.000 |
| MRAC(Curvature)<sub>1.0-1.5PD</sub> | 238.120 | 574.165 | 0.172 | 0.678 | >100 | 0.000 |
| MRAC(Curvature)<sub>1.5-2.0PD</sub> | 1869.606 | 733.527 | 6.496 | 0.011 | - | >100 |
| MRAC(Curvature)<sub>2.0-2.5PD</sub> | -502.066 | 615.970 | 0.664 | 0.415 | 0.000 | 0.000->100 |
| MRV(Venular)T | 2098.103 | 839.390 | 6.248 | 0.012 | - | >100- |
| MRVC(Curvature)<sub>0.5-1.0PD</sub> | 1449.553 | 628.230 | 5.324 | 0.021 | - | >100- |
| MRVC(Curvature)<sub>1.0-1.5PD</sub> | 799.474 | 622.227 | 1.651 | 0.199 | - | 0.000 |
| MRVC(Curvature)<sub>1.5-2.0PD</sub> | 897.497 | 519.227 | 2.988 | 0.084 | - | 0.000 |
| MRVC(Curvature)<sub>2.0-2.5PD</sub> | 887.958 | 592.910 | 2.243 | 0.134 | - | 0.000 |
| VD | 4.724 | 8.387 | 0.317 | 0.573 | 112.625 | 0.000->100 |
| MRAC | -0.837 | 0.151 | 30.613 | <0.001 | 0.433 | 0.322-0.583 |
| MRAC(Caliber)<sub>0.5-1.0PD</sub> | -0.150 | 0.025 | 35.532 | <0.001 | 0.860 | 0.819-0.904 |
| MRAC(Caliber)<sub>1.0-1.5PD</sub> | -0.201 | 0.032 | 40.569 | <0.001 | 0.818 | 0.769-0.870 |
| MRAC(Caliber)<sub>1.5-2.0PD</sub> | -0.179 | 0.029 | 37.822 | <0.001 | 0.836 | 0.790-0.885 |
| MRAC(Caliber)<sub>2.0-2.5PD</sub> | -0.190 | 0.031 | 37.744 | <0.001 | 0.827 | 0.778-0.879 |
| MRV(Venular)C | -0.115 | 0.020 | 33.157 | <0.001 | 0.892 | 0.857-0.927 |
| MRV(Venular)C<sub>0.5-1.0PD</sub> | -0.060 | 0.012 | 24.792 | <0.001 | 0.942 | 0.920-0.964 |
| MRV(Venular)C<sub>1.0-1.5PD</sub> | -0.057 | 0.013 | 21.007 | <0.001 | 0.944 | 0.921-0.968 |
| MRV(Venular)C<sub>1.5-2.0PD</sub> | -0.073 | 0.017 | 19.617 | <0.001 | 0.929 | 0.900-0.960 |
| MRV(Venular)C<sub>2.0-2.5PD</sub> | -0.051 | 0.014 | 13.501 | <0.001 | 0.950 | 0.924-0.976 |
| AVR | -7.336 | 2.099 | 12.217 | <0.001 | 0.001 | 0.000-0.040 |
| AVR <sub>0.5-1.0PD</sub> | -2.133 | 1.240 | 2.959 | 0.085 | 0.118 | 0.010-1.346 |
| AVR <sub>1.0-1.5PD</sub> | -5.514 | 1.644 | 11.249 | 0.001 | 0.004 | 0.000-0.101 |
| AVR <sub>1.5-2.0PD</sub> | -5.721 | 1.545 | 13.708 | <0.001 | 0.003 | 0.000-0.068 |
| AVR <sub>2.0-2.5PD</sub> | -6.144 | 1.515 | 16.454 | <0.001 | 0.002 | 0.000-0.042 |
| DA | 0.000 | 0.000 | 15.825 | <0.001 | 1.000 | 1.000-1.000 |
| HDD | -0.001 | 0.001 | 3.553 | 0.059 | 0.999 | 0.998-1.000 |
| VDD | -0.002 | 0.001 | 6.862 | 0.009 | 0.998 | 0.997-1.000 |
| ODR | 0.283 | 1.656 | 0.029 | 0.864 | 1.327 | 0.052-34.113 |
| ODT (D) | -0.004 | 0.002 | 3.036 | 0.081 | 0.996 | 0.992-1.000 |
| MRW | 0.000 | 0.000 | 3.598 | 0.058 | 1.000 | 0.999-1.000 |
| DDM | -0.001 | 0.000 | 11.387 | 0.001 | 0.999 | 0.998-0.999 |
| ODAR | 0.164 | 0.788 | 0.043 | 0.835 | 1.178 | 0.252-5.517 |
| MFD | 0.000 | 0.000 | 6.993 | 0.008 | 1.000 | 1.000-1.001 |
| OCA | 0.000 | 0.000 | 13.808 | 0.000 | 1.000 | 1.000-1.000 |
| CHD | -0.001 | 0.001 | 3.936 | 0.047 | 0.999 | 0.998-1.000 |
| CVD | -0.001 | 0.001 | 3.358 | 0.067 | 0.999 | 0.9981.000 |
| CIR-C | -0.059 | 1.607 | 0.001 | 0.971 | 0.943 | 0.040-21.989 |
| CDR-A | -1.320 | 1.602 | 0.680 | 0.410 | 0.267 | 0.012-6.165 |
| HCDR | -1.556 | 1.472 | 1.118 | 0.290 | 0.211 | 0.012-3.776 |
| VCDR | -0.647 | 1.607 | 0.162 | 0.687 | 0.524 | 0.022-12.210 |
| Rim-I | -0.003 | 0.002 | 2.275 | 0.131 | 0.997 | 0.994-1.001 |
| Rim-N | 0.000 | 0.001 | 0.001 | 0.969 | 1.000 | 0.997-1.003 |
| Rim-S | -0.002 | 0.002 | 1.682 | 0.195 | 0.998 | 0.995-1.001 |
| Rim-T | 0.000 | 0.001 | 0.037 | 0.848 | 1.000 | 0.998-1.003 |
| Max Rim-Cup D | -0.001 | 0.001 | 5.346 | 0.021 | 0.999 | 0.997-1.000 |
| Min Rim | -0.001 | 0.002 | 0.522 | 0.470 | 0.999 | 0.995-1.002 |

MBA, Mean Branching Angle; FD-RVN,Fractal Dimension of the Retinal Vascular Network; MRVC(Caliber) , Mean Retinal Vessel Caliber; MRVC(Caliber)<sub>0.5-1.0PD</sub>, Mean Retinal Vessel Caliber within 0.5-1.0 PD Zone; MRV(Vessel)T, Mean Retinal Vessel Tortuosity; MRVκ<sub>0.5-1.0PD</sub>, Mean Retinal Vessel Curvature within 0.5-1.0 PD Zone; MRAT, Mean Retinal Arteriolar Tortuosity; MRAC(Curvature)<sub>0.5-1.0PD</sub>, Mean Retinal Arteriolar Curvature within 0.5-1.0 PD Zone; MRV(Venular)T, Mean Retinal Venular Tortuosity; MRVC(Curvature)<sub>0.5-1.0PD</sub> ,Mean Retinal Venular Curvature within 0.5-1.0 PD Zone; VD, Vessel Density; MRAC, Mean Retinal Arteriolar Caliber ; MRAC(Caliber)<sub>0.5-1.0PD</sub> , Mean Retinal Arteriolar Caliber within 0.5-1.0 PD Zone; MRV(Venular)C, Mean Retinal Venular Caliber ; MRV(Venular)C<sub>0.5-1.0PD</sub>, Mean Retinal Venular Caliber within 0.5-1.0 PD Zone; AVR, Arteriolar-to-Venular Ratio; DA, Optic Disc Area; HDD, Horizontal Diameter of the Optic Disc; VDD, Vertical Optic Disc Diameter; ODR, Optic Disc Roundness; ODT (D), Optic Disc Tilt; MRW, Minimum Rim Width; DDM, Disc-Macula Distance; ODAR, Optic Disc Axial Ratio; MFD, Macular Fovea Distance; OCA, Optic Cup Area; CHD, Cup Horizontal Diameter; CVD, Cup Vertical Diameter; CIR-C, Cup Circularity; CDR-A, Cup-to-Disc Area Ratio; HCDR, Horizontal Cup-to-Disc Ratio; VCDR, Vertical Cup-to-Disc Ratio; Rim-I, Inferior Neuroretinal Rim Width; Rim-N, Nasal Neuroretinal Rim Width; Rim-S, Superior Neuroretinal Rim Width; Rim-T, Temporal Neuroretinal Rim Width; Max Rim-Cup D, Maximum Rim-to-Cup Distance; Min Rim, Minimum Neuroretinal Rim Width; Results of univariate logistic regression are presented as odds ratios (OR) with 95% confidence intervals (CI). An OR > 1 indicates increased odds of the outcome, and an OR < 1 indicates decreased odds; P < 0.05 was considered significant.

Supplemental Table 9. Univariable logistic regression analysis of the primary cohort for the T2DM group and the CHD with T2DM group.

|  | B | SE | Wald | *P* | OR | 95％*CI* |
| --- | --- | --- | --- | --- | --- | --- |
| Age | -0.040 | 0.030 | 1.772 | 0.183 | 0.961 | 0.906-1.019 |
| Sex |  |  |  |  |  |  |
| Male | 0.727 | 0.570 | 1.628 | 0.202 | 2.068 | 0.677-6.316 |
| Female | Ref. |  |  |  |  |  |
| Smoking history |  |  |  |  |  |  |
| No smoking | Ref. |  |  |  |  |  |
| Still smoking | 0.093 | 0.585 | 0.025 | 0.874 | 1.097 | 0.349-3.454 |
| Drinking history |  |  |  |  |  |  |
| No drinking | Ref. |  |  |  |  |  |
| Still drinking | -0.949 | 0.741 | 1.641 | 0.200 | 0.387 | 0.091-1.654 |
| FBG | 0.015 | 0.107 | 0.019 | 0.891 | 1.015 | 0.823-1.251 |
| Cr | 0.006 | 0.016 | 0.151 | 0.698 | 1.006 | 0.976-1.037 |
| Lp(a) | 0.014 | 0.012 | 1.275 | 0.259 | 1.014 | 0.990-1.038 |
| TG | -0.259 | 0.219 | 1.402 | 0.236 | 0.772 | 0.503-1.185 |
| TC | -0.755 | 0.277 | 7.450 | 0.006 | 0.470 | 0.273-0.808 |
| LDL | -0.901 | 0.340 | 7.049 | 0.008 | 0.406 | 0.209-0.790 |
| HDL | -1.165 | 0.849 | 1.885 | 0.170 | 0.312 | 0.059-1.646 |
| HbA1c | 0.880 | 0.379 | 5.390 | 0.020 | 2.412 | 1.147-5.072 |
| MBA | -0.024 | 0.024 | 0.994 | 0.319 | 0.976 | 0.930-1.024 |
| FD-RVN | -24.853 | 8.481 | 8.588 | 0.003 | 0.000 | 0.000 |
| MRVC(Caliber) | 0.112 | 0.037 | 9.103 | 0.003 | 1.118 | 1.040-1.202 |
| MRVC(Caliber)<sub>0.5–1.0PD</sub> | 0.061 | 0.031 | 3.789 | 0.052 | 1.063 | 1.000-1.131 |
| MRVC(Caliber)<sub>1.0–1.5PD</sub> | 0.085 | 0.035 | 5.716 | 0.017 | 1.088 | 1.015-1.166 |
| MRVC(Caliber)<sub>1.5–2.0PD</sub> | 0.078 | 0.037 | 4.455 | 0.035 | 1.081 | 1.006-1.162 |
| MRVC(Caliber)<sub>2.0–2.5PD</sub> | 0.050 | 0.031 | 2.545 | 0.111 | 1.051 | 0.989-1.118 |
| MRV(Vessel)T | -3013.122 | 2034.302 | 2.194 | 0.139 | 0.000 | 0.000 |
| MRVκ<sub>0.5–1.0PD</sub> | -3865.044 | 1916.274 | 4.068 | 0.044 | 0.000 | 0.000 |
| MRVκ<sub>1.0–1.5PD</sub> | -1047.404 | 1752.723 | 0.357 | 0.550 | 0.000 | 0.000 |
| MRVκ<sub>1.5–2.0PD</sub> | -2289.431 | 1598.935 | 2.050 | 0.152 | 0.000 | 0.000 |
| MRVκ<sub>2.0–2.5PD</sub> | -2687.915 | 1792.065 | 2.250 | 0.134 | 0.000 | 0.000 |
| MRAT | -1465.134 | 1880.542 | 0.607 | 0.436 | 0.000 | 0.000 |
| MRAC(Curvature)<sub>0.5-1.0PD</sub> | -1794.744 | 1389.045 | 1.669 | 0.196 | 0.000 | 0.000 |
| MRAC(Curvature)<sub>1.0-1.5PD</sub> | -943.531 | 1139.531 | 0.686 | 0.408 | 0.000 | 0.000 |
| MRAC(Curvature)<sub>1.5-2.0PD</sub> | -1765.127 | 1360.959 | 1.682 | 0.195 | 0.000 | 0.000 |
| MRAC(Curvature)<sub>2.0-2.5PD</sub> | -96.424 | 829.959 | 0.013 | 0.908 | 0.000 | 0.000 |
| MRV(Venular)T | -2496.372 | 1672.926 | 2.227 | 0.136 | 0.000 | 0.000 |
| MRVC(Curvature)<sub>0.5-1.0PD</sub> | -1487.586 | 1236.510 | 1.447 | 0.229 | 0.000 | 0.000 |
| MRVC(Curvature)<sub>1.0-1.5PD</sub> | -3.500 | 1293.776 | 0.000 | 0.998 | 0.030 | 0.000 |
| MRVC(Curvature)<sub>1.5-2.0PD</sub> | -1488.628 | 1140.334 | 1.704 | 0.192 | 0.000 | 0.000 |
| MRVC(Curvature)<sub>2.0-2.5PD</sub> | -2020.837 | 1397.746 | 2.090 | 0.148 | 0.000 | 0.000 |
| VD | -66.278 | 22.363 | 8.783 | 0.003 | 0.000 | 0.000 |
| MRAC | 0.090 | 0.042 | 4.586 | 0.032 | 1.094 | 1.008-1.189 |
| MRAC(Caliber)<sub>0.5-1.0PD</sub> | 0.015 | 0.027 | 0.297 | 0.586 | 1.015 | 0.962-1.070 |
| MRAC(Caliber)<sub>1.0-1.5PD</sub> | 0.024 | 0.029 | 0.666 | 0.414 | 1.024 | 0.967-1.084 |
| MRAC(Caliber)<sub>1.5-2.0PD</sub> | 0.027 | 0.033 | 0.645 | 0.422 | 1.027 | 0.962-1.096 |
| MRAC(Caliber)<sub>2.0-2.5PD</sub> | 0.016 | 0.033 | 0.230 | 0.631 | 1.016 | 0.952-1.085 |
| MRV(Venular)C | 0.090 | 0.032 | 8.129 | 0.004 | 1.094 | 1.028-1.164 |
| MRV(Venular)C<sub>0.5-1.0PD</sub> | 0.030 | 0.018 | 2.810 | 0.094 | 1.030 | 0.995-1.067 |
| MRV(Venular)C<sub>1.0-1.5PD</sub> | 0.046 | 0.025 | 3.333 | 0.068 | 1.047 | 0.997-1.101 |
| MRV(Venular)C<sub>1.5-2.0PD</sub> | 0.049 | 0.029 | 2.918 | 0.088 | 1.050 | 0.993-1.110 |
| MRV(Venular)C<sub>2.0-2.5PD</sub> | 0.018 | 0.020 | 0.761 | 0.383 | 1.018 | 0.978-1.059 |
| AVR | -5.913 | 4.464 | 1.754 | 0.185 | 0.003 | 0.000-17.061 |
| AVR <sub>0.5-1.0PD</sub> | -2.762 | 2.901 | 0.906 | 0.341 | 0.063 | 0.000-18.611 |
| AVR <sub>1.0-1.5PD</sub> | -2.024 | 2.618 | 0.598 | 0.439 | 0.132 | 0.001-22.356 |
| AVR <sub>1.5-2.0PD</sub> | -1.927 | 3.338 | 0.333 | 0.564 | 0.146 | 0.000-101.108 |
| AVR <sub>2.0-2.5PD</sub> | -0.292 | 2.840 | 0.011 | 0.918 | 0.747 | 0.003-195.028 |
| DA | 0.000 | 0.000 | 2.945 | 0.086 | 1.000 | 1.000-1.000 |
| HDD | 0.002 | 0.001 | 2.999 | 0.083 | 1.002 | 1.000-1.004 |
| VDD | 0.001 | 0.001 | 1.947 | 0.163 | 1.001 | 0.999-1.003 |
| ODR | -5.722 | 6.881 | 0.691 | 0.406 | 0.003 | 0.000-2357.086 |
| ODT (D) | 0.003 | 0.004 | 0.574 | 0.449 | 1.003 | 0.995-1.012 |
| MRW | 0.001 | 0.001 | 2.311 | 0.128 | 1.001 | 1.000-1.002 |
| DDM | 0.000 | 0.001 | 0.043 | 0.836 | 1.000 | 0.999-1.001 |
| ODAR | 2.673 | 4.408 | 0.368 | 0.544 | 14.487 | 0.003-81893.185 |
| MFD | 0.000 | 0.000 | 2.220 | 0.136 | 1.000 | 0.999-1.000 |
| OCA | 0.000 | 0.000 | 0.259 | 0.610 | 1.000 | 1.000-1.000 |
| CHD | 0.000 | 0.000 | 0.245 | 0.621 | 1.000 | 1.000-1.000 |
| CVD | 0.000 | 0.000 | 0.252 | 0.616 | 1.000 | 1.000-1.000 |
| CIR-C | -11.870 | 6.404 | 3.435 | 0.064 | 0.000 | 0.000-1.978 |
| CDR-A | -0.110 | 0.243 | 0.207 | 0.649 | 0.895 | 0.556-1.441 |
| HCDR | -0.107 | 0.239 | 0.202 | 0.653 | 0.898 | 0.562-1.436 |
| VCDR | -0.093 | 0.208 | 0.202 | 0.653 | 0.911 | 0.606-1.368 |
| Rim-I | -0.003 | 0.003 | 0.992 | 0.319 | 0.997 | 0.991-1.003 |
| Rim-N | -0.003 | 0.003 | 1.375 | 0.241 | 0.997 | 0.991-1.002 |
| Rim-S | -0.002 | 0.003 | 0.609 | 0.435 | 0.998 | 0.992-1.004 |
| Rim-T | 0.002 | 0.002 | 0.484 | 0.487 | 1.002 | 0.997-1.006 |
| Max Rim-Cup D | 0.000 | 0.000 | 0.279 | 0.597 | 1.000 | 1.000-1.000 |
| Min Rim | 0.000 | 0.001 | 0.087 | 0.769 | 1.000 | 0.999-1.002 |

MBA, Mean Branching Angle; FD-RVN,Fractal Dimension of the Retinal Vascular Network; MRVC(Caliber) , Mean Retinal Vessel Caliber; MRVC(Caliber)<sub>0.5-1.0PD</sub>, Mean Retinal Vessel Caliber within 0.5-1.0 PD Zone; MRV(Vessel)T, Mean Retinal Vessel Tortuosity; MRVκ<sub>0.5-1.0PD</sub>, Mean Retinal Vessel Curvature within 0.5-1.0 PD Zone; MRAT, Mean Retinal Arteriolar Tortuosity; MRAC(Curvature)<sub>0.5-1.0PD</sub>, Mean Retinal Arteriolar Curvature within 0.5-1.0 PD Zone; MRV(Venular)T, Mean Retinal Venular Tortuosity; MRVC(Curvature)<sub>0.5-1.0PD</sub> ,Mean Retinal Venular Curvature within 0.5-1.0 PD Zone; VD, Vessel Density; MRAC, Mean Retinal Arteriolar Caliber ; MRAC(Caliber)<sub>0.5-1.0PD</sub> , Mean Retinal Arteriolar Caliber within 0.5-1.0 PD Zone; MRV(Venular)C, Mean Retinal Venular Caliber ; MRV(Venular)C<sub>0.5-1.0PD</sub>, Mean Retinal Venular Caliber within 0.5-1.0 PD Zone; AVR, Arteriolar-to-Venular Ratio; DA, Optic Disc Area; HDD, Horizontal Diameter of the Optic Disc; VDD, Vertical Optic Disc Diameter; ODR, Optic Disc Roundness; ODT (D), Optic Disc Tilt; MRW, Minimum Rim Width; DDM, Disc-Macula Distance; ODAR, Optic Disc Axial Ratio; MFD, Macular Fovea Distance; OCA, Optic Cup Area; CHD, Cup Horizontal Diameter; CVD, Cup Vertical Diameter; CIR-C, Cup Circularity; CDR-A, Cup-to-Disc Area Ratio; HCDR, Horizontal Cup-to-Disc Ratio; VCDR, Vertical Cup-to-Disc Ratio; Rim-I, Inferior Neuroretinal Rim Width; Rim-N, Nasal Neuroretinal Rim Width; Rim-S, Superior Neuroretinal Rim Width; Rim-T, Temporal Neuroretinal Rim Width; Max Rim-Cup D, Maximum Rim-to-Cup Distance; Min Rim, Minimum Neuroretinal Rim Width; Results of univariate logistic regression are presented as odds ratios (OR) with 95% confidence intervals (CI). An OR > 1 indicates increased odds of the outcome, and an OR < 1 indicates decreased odds; P < 0.05 was considered significant.

Supplemental Table 10. Univariable logistic regression analysis of the primary cohort for the T2DM with HTN group and the CHD with T2DM and HTN group.

|  | B | SE | Wald | *P* | OR | 95％*CI* |
| --- | --- | --- | --- | --- | --- | --- |
| Age | -0.017 | 0.018 | 0.849 | 0.357 | 0.984 | 0.950-1.019 |
| Sex |  |  |  |  |  |  |
| Male | 1.678 | 0.380 | 19.459 | 0.000 | 5.354 | 2.540-11.283 |
| Female | Ref. |  |  |  |  |  |
| Smoking history |  |  |  |  |  |  |
| No smoking | Ref. |  |  |  |  |  |
| Still smoking | 1.458 | 0.401 | 13.257 | 0.000 | 4.298 | 1.961-9.423 |
| Drinking history |  |  |  |  |  |  |
| No drinking | Ref. |  |  |  |  |  |
| Still drinking | 1.551 | 0.632 | 6.030 | 0.014 | 1.551 | 1.368-16.262 |
| FBG | -0.048 | 0.064 | 0.571 | 0.450 | 0.953 | 0.841-1.080 |
| Cr | 0.028 | 0.010 | 8.177 | 0.004 | 1.029 | 1.009-1.049 |
| Lp(a) | 0.006 | 0.007 | 0.762 | 0.383 | 1.006 | 0.993-1.020 |
| TG | -0.084 | 0.081 | 1.067 | 0.302 | 0.920 | 0.784-1.078 |
| TC | -0.747 | 0.176 | 17.917 | 0.000 | 0.474 | 0.335-0.670 |
| LDL | -0.683 | 0.189 | 13.118 | 0.000 | 0.505 | 0.349-0.731 |
| HDL | -1.602 | 0.567 | 7.972 | 0.005 | 0.202 | 0.066-0.613 |
| HbA1c | 0.221 | 0.164 | 1.828 | 0.176 | 1.248 | 0.905-1.719 |
| MBA | 0.046 | 0.017 | 7.556 | 0.006 | 1.047 | 1.013-1.081 |
| FD-RVN | 4.426 | 2.377 | 3.466 | 0.063 | 83.590 | 0.792-8825.609 |
| MRVC(Caliber) | -0.067 | 0.019 | 12.633 | 0.000 | 0.935 | 0.902-0.970 |
| MRVC(Caliber)<sub>0.5–1.0PD</sub> | -0.020 | 0.013 | 2.408 | 0.121 | 0.981 | 0.957-1.005 |
| MRVC(Caliber)<sub>1.0–1.5PD</sub> | -0.032 | 0.015 | 4.473 | 0.034 | 0.968 | 0.940-0.998 |
| MRVC(Caliber)<sub>1.5–2.0PD</sub> | -0.035 | 0.016 | 4.788 | 0.029 | 0.966 | 0.936-0.996 |
| MRVC(Caliber)<sub>2.0–2.5PD</sub> | -0.036 | 0.015 | 5.478 | 0.019 | 0.965 | 0.936-0.994 |
| MRV(Vessel)T | 1855.209 | 1136.121 | 2.666 | 0.102 | - | - |
| MRVκ<sub>0.5–1.0PD</sub> | 794.760 | 915.134 | 0.754 | 0.385 | - | - |
| MRVκ<sub>1.0–1.5PD</sub> | 1013.073 | 840.677 | 1.452 | 0.228 | - | - |
| MRVκ<sub>1.5–2.0PD</sub> | 553.928 | 740.769 | 0.559 | 0.455 | >100 | - |
| MRVκ<sub>2.0–2.5PD</sub> | 2037.044 | 871.497 | 5.463 | 0.019 | - |  |
| MRAT | 1156.815 | 1062.334 | 1.186 | 0.276 | - | - |
| MRAC(Curvature)<sub>0.5-1.0PD</sub> | 297.669 | 665.412 | 0.200 | 0.655 | >100 | - |
| MRAC(Curvature)<sub>1.0-1.5PD</sub> | 474.019 | 679.953 | 0.486 | 0.486 | >100 | - |
| MRAC(Curvature)<sub>1.5-2.0PD</sub> | 981.190 | 724.173 | 1.836 | 0.175 | - | - |
| MRAC(Curvature)<sub>2.0-2.5PD</sub> | 947.690 | 868.182 | 1.192 | 0.275 | - | - |
| MRV(Venular)T | 1604.731 | 943.362 | 2.894 | 0.089 | - | - |
| MRVC(Curvature)<sub>0.5-1.0PD</sub> | 882.691 | 750.829 | 1.382 | 0.240 | - | - |
| MRVC(Curvature)<sub>1.0-1.5PD</sub> | 943.577 | 695.091 | 1.843 | 0.175 | - | - |
| MRVC(Curvature)<sub>1.5-2.0PD</sub> | 387.697 | 592.321 | 0.428 | 0.513 | >100 | - |
| MRVC(Curvature)<sub>2.0-2.5PD</sub> | 1606.475 | 663.300 | 5.866 | 0.015 | - | - |
| VD | 17.478 | 8.915 | 3.843 | 0.050 | 38948105.050 | 1.004->100 |
| MRAC | -0.197 | 0.038 | 26.381 | <0.001 | 0.821 | 0.762-0.885 |
| MRAC(Caliber)<sub>0.5-1.0PD</sub> | -0.077 | 0.022 | 12.682 | <0.001 | 0.926 | 0.888-0.966 |
| MRAC(Caliber)<sub>1.0-1.5PD</sub> | -0.075 | 0.021 | 12.765 | <0.001 | 0.928 | 0.890-0.967 |
| MRAC(Caliber)<sub>1.5-2.0PD</sub> | -0.086 | 0.023 | 14.400 | <0.001 | 0.918 | 0.878-0.959 |
| MRAC(Caliber)<sub>2.0-2.5PD</sub> | -0.086 | 0.021 | 16.032 | <0.001 | 0.918 | 0.880-0.957 |
| MRV(Venular)C | -0.045 | 0.016 | 7.500 | 0.006 | 0.956 | 0.926-0.987 |
| MRV(Venular)C<sub>0.5-1.0PD</sub> | -0.019 | 0.011 | 2.956 | 0.086 | 0.981 | 0.959-1.003 |
| MRV(Venular)C<sub>1.0-1.5PD</sub> | -0.029 | 0.014 | 4.510 | 0.034 | 0.971 | 0.946-0.998 |
| MRV(Venular)C<sub>1.5-2.0PD</sub> | -0.011 | 0.014 | 0.641 | 0.423 | 0.989 | 0.961-1.017 |
| MRV(Venular)C<sub>2.0-2.5PD</sub> | -0.021 | 0.013 | 2.738 | 0.098 | 0.979 | 0.955-1.004 |
| AVR | -6.911 | 2.408 | 8.238 | 0.004 | 0.001 | 0.000-0.112 |
| AVR <sub>0.5-1.0PD</sub> | -1.753 | 1.438 | 1.488 | 0.223 | 0.173 | 0.010-2.898 |
| AVR <sub>1.0-1.5PD</sub> | -3.209 | 1.720 | 3.482 | 0.062 | 0.040 | 0.001-1.175 |
| AVR <sub>1.5-2.0PD</sub> | -6.092 | 1.902 | 10.257 | 0.001 | 0.002 | 0.000-0.094 |
| AVR <sub>2.0-2.5PD</sub> | -3.977 | 1.553 | 6.557 | 0.010 | 0.019 | 0.001-0.393 |
| DA | 0.000 | 0.000 | 5.528 | 0.019 | 1.000 | 1.000-1.000 |
| HDD | 0.000 | 0.001 | 0.549 | 0.459 | 1.000 | 0.998-1.001 |
| VDD | -0.001 | 0.001 | 0.656 | 0.418 | 0.999 | 0.998-1.001 |
| ODR | -1.583 | 2.439 | 0.421 | 0.516 | 0.205 | 0.002-24.487 |
| ODT (D) | -0.005 | 0.003 | 4.058 | 0.044 | 0.995 | 0.990-1.000 |
| MRW | 0.000 | 0.000 | 0.012 | 0.913 | 1.000 | 0.999-1.000 |
| DDM | -0.002 | 0.001 | 9.050 | 0.003 | 0.998 | 0.997-0.999 |
| ODAR | 0.521 | 1.306 | 0.160 | 0.690 | 1.684 | 0.130-21.763 |
| MFD | 0.000 | 0.000 | 3.799 | 0.051 | 1.000 | 1.000-1.001 |
| OCA | 0.000 | 0.000 | 2.596 | 0.107 | 1.000 | 1.000-1.000 |
| CHD | -0.001 | 0.001 | 0.962 | 0.327 | 0.999 | 0.998-1.001 |
| CVD | 0.000 | 0.001 | 0.412 | 0.521 | 1.000 | 0.998-1.001 |
| CIR-C | 1.565 | 2.530 | 0.383 | 0.536 | 4.781 | 0.034-680.302 |
| CDR-A | 0.118 | 2.020 | 0.003 | 0.953 | 1.125 | 0.021-59.013 |
| HCDR | -0.547 | 1.742 | 0.099 | 0.754 | 0.579 | 0.019-17.598 |
| VCDR | 0.713 | 2.046 | 0.121 | 0.728 | 2.040 | 0.037-112.380 |
| Rim-I | -0.002 | 0.002 | 1.018 | 0.313 | 0.998 | 0.994-1.002 |
| Rim-N | 0.001 | 0.002 | 0.416 | 0.519 | 1.001 | 0.998-1.004 |
| Rim-S | -0.004 | 0.002 | 4.355 | 0.037 | 0.996 | 0.992-1.000 |
| Rim-T | -0.004 | 0.002 | 5.103 | 0.024 | 0.996 | 0.993-0.999 |
| Max Rim-Cup D | -0.001 | 0.001 | 0.880 | 0.348 | 0.999 | 0.998-1.001 |
| Min Rim | -0.005 | 0.002 | 3.533 | 0.060 | 0.995 | 0.991-1.000 |

MBA, Mean Branching Angle; FD-RVN,Fractal Dimension of the Retinal Vascular Network; MRVC(Caliber) , Mean Retinal Vessel Caliber; MRVC(Caliber)<sub>0.5-1.0PD</sub>, Mean Retinal Vessel Caliber within 0.5-1.0 PD Zone; MRV(Vessel)T, Mean Retinal Vessel Tortuosity; MRVκ<sub>0.5-1.0PD</sub>, Mean Retinal Vessel Curvature within 0.5-1.0 PD Zone; MRAT, Mean Retinal Arteriolar Tortuosity; MRAC(Curvature)<sub>0.5-1.0PD</sub>, Mean Retinal Arteriolar Curvature within 0.5-1.0 PD Zone; MRV(Venular)T, Mean Retinal Venular Tortuosity; MRVC(Curvature)<sub>0.5-1.0PD</sub> ,Mean Retinal Venular Curvature within 0.5-1.0 PD Zone; VD, Vessel Density; MRAC, Mean Retinal Arteriolar Caliber ; MRAC(Caliber)<sub>0.5-1.0PD</sub> , Mean Retinal Arteriolar Caliber within 0.5-1.0 PD Zone; MRV(Venular)C, Mean Retinal Venular Caliber ; MRV(Venular)C<sub>0.5-1.0PD</sub>, Mean Retinal Venular Caliber within 0.5-1.0 PD Zone; AVR, Arteriolar-to-Venular Ratio; DA, Optic Disc Area; HDD, Horizontal Diameter of the Optic Disc; VDD, Vertical Optic Disc Diameter; ODR, Optic Disc Roundness; ODT (D), Optic Disc Tilt; MRW, Minimum Rim Width; DDM, Disc-Macula Distance; ODAR, Optic Disc Axial Ratio; MFD, Macular Fovea Distance; OCA, Optic Cup Area; CHD, Cup Horizontal Diameter; CVD, Cup Vertical Diameter; CIR-C, Cup Circularity; CDR-A, Cup-to-Disc Area Ratio; HCDR, Horizontal Cup-to-Disc Ratio; VCDR, Vertical Cup-to-Disc Ratio; Rim-I, Inferior Neuroretinal Rim Width; Rim-N, Nasal Neuroretinal Rim Width; Rim-S, Superior Neuroretinal Rim Width; Rim-T, Temporal Neuroretinal Rim Width; Max Rim-Cup D, Maximum Rim-to-Cup Distance; Min Rim, Minimum Neuroretinal Rim Width; Results of univariate logistic regression are presented as odds ratios (OR) with 95% confidence intervals (CI). An OR > 1 indicates increased odds of the outcome, and an OR < 1 indicates decreased odds; P < 0.05 was considered significant.

Supplemental Table 11. Univariable logistic regression analysis of the PSM cohort for the matched control group and the CHD group.

|  | B | SE | Wald | *P* | OR | 95％*CI* |
| --- | --- | --- | --- | --- | --- | --- |
| Age | -0.002 | 0.021 | 0.013 | 0.910 | 0.998 | 0.958-1.039 |
| Sex |  |  |  |  |  |  |
| Male | 0.000 | 0.493 | 0.000 | 1.000 | 1.000 | 0.381-2.627 |
| Female | Ref. |  |  |  |  |  |
| Smoking history |  |  |  |  |  |  |
| No smoking | Ref. |  |  |  |  |  |
| Still smoking | 0.000 | 0.499 | 0.000 | 1.000 | 1.000 | 0.376-2.660 |
| Drinking history |  |  |  |  |  |  |
| No drinking | Ref. |  |  |  |  |  |
| Still drinking | 0.217 | 0.661 | 0.108 | 0.742 | 1.243 | 0.340-4.540 |
| FBG | -0.051 | 0.130 | 0.154 | 0.695 | 0.950 | 0.736-1.226 |
| Cr | -0.003 | 0.012 | 0.050 | 0.822 | 0.997 | 0.975-1.020 |
| Lp(a) | -0.003 | 0.009 | 0.114 | 0.735 | 0.997 | 0.980-1.014 |
| TG | -0.013 | 0.248 | 0.003 | 0.959 | 0.987 | 0.607-1.606 |
| TC | -0.004 | 0.213 | 0.000 | 0.985 | 0.996 | 0.656-1.513 |
| LDL | 0.055 | 0.239 | 0.054 | 0.817 | 1.057 | 0.661-1.690 |
| HDL | -0.785 | 0.781 | 1.009 | 0.315 | 0.456 | 0.099-2.109 |
| HbA1c | 0.140 | 0.222 | 0.399 | 0.528 | 1.150 | 0.745-1.776 |
| MBA | -0.041 | 0.025 | 2.678 | 0.102 | 0.960 | 0.914-1.008 |
| FD-RVN | -15.139 | 6.049 | 6.263 | 0.012 | 0.000 | 0.000-0.038 |
| MRVC(Caliber) | 0.297 | 0.079 | 14.137 | 0.000 | 1.346 | 1.153-1.572 |
| MRVC(Caliber)<sub>0.5–1.0PD</sub> | 0.043 | 0.026 | 2.669 | 0.102 | 1.044 | 0.991-1.100 |
| MRVC(Caliber)<sub>1.0–1.5PD</sub> | 0.049 | 0.028 | 3.174 | 0.075 | 1.051 | 0.995-1.109 |
| MRVC(Caliber)<sub>1.5–2.0PD</sub> | 0.088 | 0.033 | 7.176 | 0.007 | 1.091 | 1.024-1.164 |
| MRVC(Caliber)<sub>2.0–2.5PD</sub> | 0.083 | 0.031 | 7.070 | 0.008 | 1.086 | 1.022-1.154 |
| MRV(Vessel)T | -2304.171 | 1916.510 | 1.445 | 0.229 | 0.000 | 0.000 |
| MRVκ<sub>0.5–1.0PD</sub> | -1344.507 | 1265.801 | 1.128 | 0.288 | 0.000 | 0.000 |
| MRVκ<sub>1.0–1.5PD</sub> | -442.880 | 953.854 | 0.216 | 0.642 | 0.000 | 0.000 |
| MRVκ<sub>1.5–2.0PD</sub> | -1952.979 | 1255.403 | 2.420 | 0.120 | 0.000 | 0.000->100 |
| MRVκ<sub>2.0–2.5PD</sub> | -680.133 | 1087.213 | 0.391 | 0.532 | 0.000 | 0.000 |
| MRAT | -576.036 | 1572.820 | 0.134 | 0.714 | 0.000 | 0.000 |
| MRAC(Curvature)<sub>0.5-1.0PD</sub> | -149.004 | 981.288 | 0.023 | 0.879 | 0.000 | 0.000 |
| MRAC(Curvature)<sub>1.0-1.5PD</sub> | 88.940 | 983.752 | 0.008 | 0.928 | >100 | 0.000 |
| MRAC(Curvature)<sub>1.5-2.0PD</sub> | -1569.724 | 1127.498 | 1.938 | 0.164 | 0.000 | 0.000->100 |
| MRAC(Curvature)<sub>2.0-2.5PD</sub> | -93.259 | 1039.503 | 0.008 | 0.929 | 0.000 | 0.000 |
| MRV(Venular)T | -2110.573 | 1528.019 | 1.908 | 0.167 | 0.000 | 0.000 |
| MRVC(Curvature)<sub>0.5-1.0PD</sub> | -1361.949 | 1064.167 | 1.638 | 0.201 | 0.000 | 0.000 |
| MRVC(Curvature)<sub>1.0-1.5PD</sub> | -230.989 | 704.771 | 0.107 | 0.743 | 0.000 | 0.000 |
| MRVC(Curvature)<sub>1.5-2.0PD</sub> | -1135.878 | 963.512 | 1.390 | 0.238 | 0.000 | 0.000 |
| MRVC(Curvature)<sub>2.0-2.5PD</sub> | -871.712 | 884.495 | 0.971 | 0.324 | 0.000 | 0.000 |
| VD | -42.974 | 18.303 | 5.513 | 0.019 | 0.000 | 0.000-0.001 |
| MRAC | 0.078 | 0.047 | 2.709 | 0.100 | 1.081 | 0.985-1.186 |
| MRAC(Caliber)<sub>0.5-1.0PD</sub> | 0.012 | 0.032 | 0.147 | 0.701 | 1.012 | 0.951-1.077 |
| MRAC(Caliber)<sub>1.0-1.5PD</sub> | 0.008 | 0.030 | 0.066 | 0.797 | 1.008 | 0.951-1.068 |
| MRAC(Caliber)<sub>1.5-2.0PD</sub> | 0.008 | 0.030 | 0.075 | 0.784 | 1.008 | 0.950-1.070 |
| MRAC(Caliber)<sub>2.0-2.5PD</sub> | -0.010 | 0.028 | 0.133 | 0.715 | 0.990 | 0.936-1.046 |
| MRV(Venular)C | 0.278 | 0.069 | 16.262 | <0.001 | 1.320 | 1.154-1.511 |
| MRV(Venular)C<sub>0.5-1.0PD</sub> | 0.068 | 0.024 | 8.312 | 0.004 | 1.070 | 1.022-1.121 |
| MRV(Venular)C<sub>1.0-1.5PD</sub> | 0.074 | 0.028 | 7.031 | 0.008 | 1.077 | 1.019-1.137 |
| MRV(Venular)C<sub>1.5-2.0PD</sub> | 0.095 | 0.030 | 9.977 | 0.002 | 1.100 | 1.037-1.166 |
| MRV(Venular)C<sub>2.0-2.5PD</sub> | 0.057 | 0.020 | 8.071 | 0.004 | 1.059 | 1.018-1.101 |
| AVR | -26.659 | 6.825 | 15.258 | <0.001 | 0.000 | 0.000 |
| AVR <sub>0.5-1.0PD</sub> | -10.203 | 4.166 | 5.998 | 0.014 | 0.000 | 0.000-0.130 |
| AVR <sub>1.0-1.5PD</sub> | -6.814 | 3.839 | 3.150 | 0.076 | 0.001 | 0.000-2.037 |
| AVR <sub>1.5-2.0PD</sub> | -7.137 | 2.892 | 6.091 | 0.014 | 0.001 | 0.000-0.230 |
| AVR <sub>2.0-2.5PD</sub> | -6.098 | 2.146 | 8.072 | 0.004 | 0.002 | 0.000-0.151 |
| DA | -6.098 | 2.146 | 8.072 | 0.004 | 0.002 | 0.000-0.151 |
| HDD | 0.002 | 0.001 | 3.064 | 0.080 | 1.002 | 1.000-1.004 |
| VDD | 0.001 | 0.001 | 0.462 | 0.497 | 1.001 | 0.999-1.003 |
| ODR | 3.728 | 3.149 | 1.401 | 0.237 | 41.578 | 0.087-19920.858 |
| ODT (D) | 0.003 | 0.004 | 0.649 | 0.420 | 1.003 | 0.996-1.011 |
| MRW | 0.001 | 0.000 | 10.943 | 0.001 | 1.001 | 1.000-1.002 |
| DDM | -0.001 | 0.001 | 1.712 | 0.191 | 0.999 | 0.998-1.000 |
| ODAR | -2.306 | 2.277 | 1.026 | 0.311 | 0.100 | 0.001-8.638 |
| MFD | -0.001 | 0.000 | 13.560 | 0.000 | 0.999 | 0.998-0.999 |
| OCA | 0.000 | 0.000 | 1.005 | 0.316 | 1.000 | 1.000-1.000 |
| CHD | 0.002 | 0.001 | 1.923 | 0.166 | 1.002 | 0.999-1.004 |
| CVD | 0.001 | 0.001 | 0.629 | 0.428 | 1.001 | 0.999-1.003 |
| CIR-C | -0.449 | 2.356 | 0.036 | 0.849 | 0.638 | 0.006-64.627 |
| CDR-A | 1.974 | 3.291 | 0.360 | 0.549 | 7.200 | 0.011-4553.717 |
| HCDR | 1.817 | 2.701 | 0.452 | 0.501 | 6.153 | 0.031-1226.292 |
| VCDR | 2.179 | 3.171 | 0.472 | 0.492 | 8.838 | 0.018-4420.274 |
| Rim-I | 0.001 | 0.002 | 0.179 | 0.672 | 1.001 | 0.997-1.005 |
| Rim-N | 0.000 | 0.002 | 0.019 | 0.891 | 1.000 | 0.996-1.004 |
| Rim-S | 0.000 | 0.002 | 0.015 | 0.902 | 1.000 | 0.996-1.005 |
| Rim-T | 0.002 | 0.002 | 0.924 | 0.336 | 1.002 | 0.998-1.006 |
| Max Rim-Cup D | 0.002 | 0.001 | 1.684 | 0.194 | 1.002 | 0.999-1.004 |
| Min Rim | 0.000 | 0.002 | 0.010 | 0.919 | 1.000 | 0.996-1.005 |

MBA, Mean Branching Angle; FD-RVN,Fractal Dimension of the Retinal Vascular Network; MRVC(Caliber) , Mean Retinal Vessel Caliber; MRVC(Caliber)<sub>0.5-1.0PD</sub>, Mean Retinal Vessel Caliber within 0.5-1.0 PD Zone; MRV(Vessel)T, Mean Retinal Vessel Tortuosity; MRVκ<sub>0.5-1.0PD</sub>, Mean Retinal Vessel Curvature within 0.5-1.0 PD Zone; MRAT, Mean Retinal Arteriolar Tortuosity; MRAC(Curvature)<sub>0.5-1.0PD</sub>, Mean Retinal Arteriolar Curvature within 0.5-1.0 PD Zone; MRV(Venular)T, Mean Retinal Venular Tortuosity; MRVC(Curvature)<sub>0.5-1.0PD</sub> ,Mean Retinal Venular Curvature within 0.5-1.0 PD Zone; VD, Vessel Density; MRAC, Mean Retinal Arteriolar Caliber ; MRAC(Caliber)<sub>0.5-1.0PD</sub> , Mean Retinal Arteriolar Caliber within 0.5-1.0 PD Zone; MRV(Venular)C, Mean Retinal Venular Caliber ; MRV(Venular)C<sub>0.5-1.0PD</sub>, Mean Retinal Venular Caliber within 0.5-1.0 PD Zone; AVR, Arteriolar-to-Venular Ratio; DA, Optic Disc Area; HDD, Horizontal Diameter of the Optic Disc; VDD, Vertical Optic Disc Diameter; ODR, Optic Disc Roundness; ODT (D), Optic Disc Tilt; MRW, Minimum Rim Width; DDM, Disc-Macula Distance; ODAR, Optic Disc Axial Ratio; MFD, Macular Fovea Distance; OCA, Optic Cup Area; CHD, Cup Horizontal Diameter; CVD, Cup Vertical Diameter; CIR-C, Cup Circularity; CDR-A, Cup-to-Disc Area Ratio; HCDR, Horizontal Cup-to-Disc Ratio; VCDR, Vertical Cup-to-Disc Ratio; Rim-I, Inferior Neuroretinal Rim Width; Rim-N, Nasal Neuroretinal Rim Width; Rim-S, Superior Neuroretinal Rim Width; Rim-T, Temporal Neuroretinal Rim Width; Max Rim-Cup D, Maximum Rim-to-Cup Distance; Min Rim, Minimum Neuroretinal Rim Width; Results of univariate logistic regression are presented as odds ratios (OR) with 95% confidence intervals (CI). An OR > 1 indicates increased odds of the outcome, and an OR < 1 indicates decreased odds; P < 0.05 was considered significant.

Supplemental Table 12. Univariable logistic regression analysis of the PSM cohort for the HTN group and the CHD with HTN group.

|  | B | SE | | Wald | | *P* | OR | 95％*CI* |
| --- | --- | --- | --- | --- | --- | --- | --- | --- |
| Age | 0.023 | | 0.018 | | 1.625 | 0.202 | 1.023 | 0.988-1.060 |
| Sex |  | |  | |  |  |  |  |
| Male | -0.158 | | 0.397 | | 0.157 | 0.691 | 0.854 | 0.392-1.861 |
| Female | Ref. | |  | |  |  |  |  |
| Smoking history |  | |  | |  |  |  |  |
| No smoking | Ref. | |  | |  |  |  |  |
| Still smoking | 0.156 | | 0.395 | | 0.156 | 0.693 | 1.169 | 0.539-2.536 |
| Drinking history |  | |  | |  |  |  |  |
| No drinking | Ref. | |  | |  |  |  |  |
| Still drinking | -0.119 | | 0.489 | | 0.060 | 0.807 | 0.887 | 0.340-2.314 |
| FBG | 0.017 | | 0.223 | | 0.006 | 0.938 | 1.018 | 0.657-1.576 |
| Cr | -0.009 | | 0.012 | | 0.507 | 0.476 | 0.991 | 0.967-1.016 |
| Lp(a) | 0.001 | | 0.008 | | 0.006 | 0.940 | 1.001 | 0.985-1.016 |
| TG | -0.063 | | 0.193 | | 0.108 | 0.742 | 0.939 | 0.644-1.369 |
| TC | 0.022 | | 0.169 | | 0.016 | 0.899 | 1.022 | 0.734-1.423 |
| LDL | -0.006 | | 0.196 | | 0.001 | 0.976 | 0.994 | 0.677-1.460 |
| HDL | 0.247 | | 0.528 | | 0.218 | 0.640 | 1.280 | 0.454-3.607 |
| HbA1c | 0.039 | | 0.396 | | 0.010 | 0.921 | 1.040 | 0.479-2.260 |
| MBA | 0.030 | | 0.017 | | 3.119 | 0.077 | 1.031 | 0.997-1.066 |
| FD-RVN | 1.001 | | 2.955 | | 0.115 | 0.735 | 2.720 | 0.008-890.953 |
| MRVC(Caliber) | -0.178 | | 0.036 | | 24.227 | 0.000 | 0.837 | 0.780-0.899 |
| MRVC(Caliber)<sub>0.5–1.0PD</sub> | -0.086 | | 0.023 | | 14.356 | 0.000 | 0.918 | 0.878-0.959 |
| MRVC(Caliber)<sub>1.0–1.5PD</sub> | -0.075 | | 0.021 | | 12.977 | 0.000 | 0.928 | 0.891-0.966 |
| MRVC(Caliber)<sub>1.5–2.0PD</sub> | -0.114 | | 0.028 | | 16.833 | 0.000 | 0.893 | 0.846-0.942 |
| MRVC(Caliber)<sub>2.0–2.5PD</sub> | -0.090 | | 0.026 | | 12.110 | 0.001 | 0.914 | 0.869-0.961 |
| MRV(Vessel)T | 2850.593 | | 1281.521 | | 4.948 | 0.026 | - | >100 |
| MRVκ<sub>0.5–1.0PD</sub> | 2839.739 | | 1057.109 | | 7.216 | 0.007 | - | . |
| MRVκ<sub>1.0–1.5PD</sub> | 1169.393 | | 960.408 | | 1.483 | 0.223 | - | 0.000 |
| MRVκ<sub>1.5–2.0PD</sub> | 1833.700 | | 933.752 | | 3.856 | 0.050 | - | 35.834 |
| MRVκ<sub>2.0–2.5PD</sub> | 899.469 | | 932.634 | | 0.930 | 0.335 | - | 0.000 |
| MRAT | 2561.829 | | 1216.694 | | 4.433 | 0.035 | - | >100 |
| MRAC(Curvature)<sub>0.5-1.0PD</sub> | 1344.788 | | 796.217 | | 2.853 | 0.091 | - | 0.000 |
| MRAC(Curvature)<sub>1.0-1.5PD</sub> | -27.568 | | 671.179 | | 0.002 | 0.967 | 0.000 | 0.000 |
| MRAC(Curvature)<sub>1.5-2.0PD</sub> | 2635.236 | | 949.345 | | 7.705 | 0.006 | - | - |
| MRAC(Curvature)<sub>2.0-2.5PD</sub> | -235.358 | | 897.370 | | 0.069 | 0.793 | 0.000 | 0.000 |
| MRV(Venular)T | 1590.912 | | 1028.978 | | 2.390 | 0.122 | - | 0.000 |
| MRVC(Curvature)<sub>0.5-1.0PD</sub> | 1447.356 | | 802.663 | | 3.251 | 0.071 | - | 0.000 |
| MRVC(Curvature)<sub>1.0-1.5PD</sub> | 895.833 | | 754.397 | | 1.410 | 0.235 | - | 0.000 |
| MRVC(Curvature)<sub>1.5-2.0PD</sub> | 455.422 | | 678.705 | | 0.450 | 0.502 | >100 | 0.000 |
| MRVC(Curvature)<sub>2.0-2.5PD</sub> | 845.037 | | 728.369 | | 1.346 | 0.246 | - | 0.000 |
| VD | -1.039 | | 10.597 | | 0.010 | 0.922 | 0.354 | 0.000->100 |
| MRAC | -1.515 | | 0.445 | | 11.602 | 0.001 | 0.220 | 0.092-0.526 |
| MRAC(Caliber)<sub>0.5-1.0PD</sub> | -0.223 | | 0.045 | | 24.930 | <0.001 | 0.800 | 0.733-0.873 |
| MRAC(Caliber)<sub>1.0-1.5PD</sub> | -0.270 | | 0.056 | | 23.620 | <0.001 | 0.764 | 0.685-0.851 |
| MRAC(Caliber)<sub>1.5-2.0PD</sub> | -0.185 | | 0.040 | | 21.534 | <0.001 | 0.831 | 0.768-0.899 |
| MRAC(Caliber)<sub>2.0-2.5PD</sub> | -0.154 | | 0.034 | | 20.145 | <0.001 | 0.858 | 0.802-0.917 |
| MRV(Venular)C | -0.095 | | 0.024 | | 15.136 | <0.001 | 0.909 | 0.867-0.954 |
| MRV(Venular)C<sub>0.5-1.0PD</sub> | -0.056 | | 0.016 | | 12.691 | <0.001 | 0.946 | 0.917-0.975 |
| MRV(Venular)C<sub>1.0-1.5PD</sub> | -0.044 | | 0.014 | | 9.349 | 0.002 | 0.957 | 0.930-0.984 |
| MRV(Venular)C<sub>1.5-2.0PD</sub> | -0.064 | | 0.020 | | 10.581 | 0.001 | 0.938 | 0.902-0.975 |
| MRV(Venular)C<sub>2.0-2.5PD</sub> | -0.035 | | 0.016 | | 4.798 | 0.028 | 0.965 | 0.935-0.996 |
| AVR | -8.378 | | 2.642 | | 10.058 | 0.002 | 0.000 | 0.000-0.041 |
| AVR <sub>0.5-1.0PD</sub> | -4.124 | | 1.696 | | 5.916 | 0.015 | 0.016 | 0.001-0.449 |
| AVR <sub>1.0-1.5PD</sub> | -6.737 | | 2.112 | | 10.174 | 0.001 | 0.001 | 0.000-0.074 |
| AVR <sub>1.5-2.0PD</sub> | -6.330 | | 2.018 | | 9.843 | 0.002 | 0.002 | 0.000-0.093 |
| AVR <sub>2.0-2.5PD</sub> | -5.736 | | 1.808 | | 10.065 | 0.002 | 0.003 | 0.000-0.112 |
| DA | 0.000 | | 0.000 | | 11.039 | 0.001 | 1.000 | 1.000-1.000 |
| HDD | -0.001 | | 0.001 | | 4.699 | 0.030 | 0.999 | 0.997-1.000 |
| VDD | -0.002 | | 0.001 | | 8.430 | 0.004 | 0.998 | 0.996-0.999 |
| ODR | -1.970 | | 2.044 | | 0.929 | 0.335 | 0.139 | 0.003-7.663 |
| ODT (D) | -0.004 | | 0.003 | | 1.654 | 0.198 | 0.996 | 0.991-1.002 |
| MRW | 0.000 | | 0.000 | | 1.282 | 0.258 | 1.000 | 0.999-1.000 |
| DDM | -0.002 | | 0.001 | | 10.343 | 0.001 | 0.998 | 0.997-0.999 |
| ODAR | 0.752 | | 0.917 | | 0.673 | 0.412 | 2.121 | 0.352-12.787 |
| MFD | 0.000 | | 0.000 | | 1.847 | 0.174 | 1.000 | 1.000-1.001 |
| OCA | 0.000 | | 0.000 | | 10.256 | 0.001 | 1.000 | 1.000-1.000 |
| CHD | -0.002 | | 0.001 | | 6.903 | 0.009 | 0.998 | 0.996-0.999 |
| CVD | -0.002 | | 0.001 | | 7.372 | 0.007 | 0.998 | 0.996-0.999 |
| CIR-C | -2.315 | | 2.348 | | 0.972 | 0.324 | 0.099 | 0.001-9.847 |
| CDR-A | -4.934 | | 2.318 | | 4.530 | 0.033 | 0.007 | 0.000-0.677 |
| HCDR | -4.182 | | 2.005 | | 4.349 | 0.037 | 0.015 | 0.000-0.777 |
| VCDR | -3.928 | | 2.219 | | 3.134 | 0.077 | 0.020 | 0.000-1.523 |
| Rim-I | -0.003 | | 0.002 | | 1.469 | 0.225 | 0.997 | 0.993-1.002 |
| Rim-N | -0.001 | | 0.002 | | 0.278 | 0.598 | 0.999 | 0.996-1.002 |
| Rim-S | -0.001 | | 0.002 | | 0.369 | 0.544 | 0.999 | 0.995-1.003 |
| Rim-T | 0.003 | | 0.002 | | 1.926 | 0.165 | 1.003 | 0.999-1.006 |
| Max Rim-Cup D | -0.002 | | 0.001 | | 6.547 | 0.011 | 0.998 | 0.997-1.000 |
| Min Rim | -0.001 | | 0.002 | | 0.509 | 0.475 | 0.999 | 0.994-1.003 |

MBA, Mean Branching Angle; FD-RVN,Fractal Dimension of the Retinal Vascular Network; MRVC(Caliber) , Mean Retinal Vessel Caliber; MRVC(Caliber)<sub>0.5-1.0PD</sub>, Mean Retinal Vessel Caliber within 0.5-1.0 PD Zone; MRV(Vessel)T, Mean Retinal Vessel Tortuosity; MRVκ<sub>0.5-1.0PD</sub>, Mean Retinal Vessel Curvature within 0.5-1.0 PD Zone; MRAT, Mean Retinal Arteriolar Tortuosity; MRAC(Curvature)<sub>0.5-1.0PD</sub>, Mean Retinal Arteriolar Curvature within 0.5-1.0 PD Zone; MRV(Venular)T, Mean Retinal Venular Tortuosity; MRVC(Curvature)<sub>0.5-1.0PD</sub> ,Mean Retinal Venular Curvature within 0.5-1.0 PD Zone; VD, Vessel Density; MRAC, Mean Retinal Arteriolar Caliber ; MRAC(Caliber)<sub>0.5-1.0PD</sub> , Mean Retinal Arteriolar Caliber within 0.5-1.0 PD Zone; MRV(Venular)C, Mean Retinal Venular Caliber ; MRV(Venular)C<sub>0.5-1.0PD</sub>, Mean Retinal Venular Caliber within 0.5-1.0 PD Zone; AVR, Arteriolar-to-Venular Ratio; DA, Optic Disc Area; HDD, Horizontal Diameter of the Optic Disc; VDD, Vertical Optic Disc Diameter; ODR, Optic Disc Roundness; ODT (D), Optic Disc Tilt; MRW, Minimum Rim Width; DDM, Disc-Macula Distance; ODAR, Optic Disc Axial Ratio; MFD, Macular Fovea Distance; OCA, Optic Cup Area; CHD, Cup Horizontal Diameter; CVD, Cup Vertical Diameter; CIR-C, Cup Circularity; CDR-A, Cup-to-Disc Area Ratio; HCDR, Horizontal Cup-to-Disc Ratio; VCDR, Vertical Cup-to-Disc Ratio; Rim-I, Inferior Neuroretinal Rim Width; Rim-N, Nasal Neuroretinal Rim Width; Rim-S, Superior Neuroretinal Rim Width; Rim-T, Temporal Neuroretinal Rim Width; Max Rim-Cup D, Maximum Rim-to-Cup Distance; Min Rim, Minimum Neuroretinal Rim Width; Results of univariate logistic regression are presented as odds ratios (OR) with 95% confidence intervals (CI). An OR > 1 indicates increased odds of the outcome, and an OR < 1 indicates decreased odds; P < 0.05 was considered significant.

Supplemental Table 13. Univariable logistic regression analysis of the PSM cohort for the T2DM group and the CHD with T2DM group.

|  | B | SE | Wald | *P* | OR | 95％*CI* |
| --- | --- | --- | --- | --- | --- | --- |
| Age | -0.024 | 0.057 | 0.179 | 0.672 | 0.976 | 0.872-1.092 |
| Sex |  |  |  |  |  |  |
| Male | 0.000 | 0.949 | 0.000 | 1.000 | 1.000 | 0.156-6.420 |
| Female | Ref. |  |  |  |  |  |
| Smoking history |  |  |  |  |  |  |
| No smoking | Ref. |  |  |  |  |  |
| Still smoking | -21.608 | 23205.422 | 0.000 | 0.999 | 0.000 | 0.000 |
| Drinking history |  |  |  |  |  |  |
| No drinking | Ref. |  |  |  |  |  |
| Still drinking | -21.454 | 28420.722 | 0.000 | 0.999 | 0.000 | 0.000 |
| FBG | -0.104 | 0.195 | 0.282 | 0.595 | 0.902 | 0.615-1.322 |
| Cr | 0.050 | 0.048 | 1.059 | 0.303 | 1.051 | 0.956-1.156 |
| Lp(a) | -0.002 | 0.017 | 0.017 | 0.896 | 0.998 | 0.966-1.031 |
| TG | -0.072 | 0.275 | 0.068 | 0.794 | 0.931 | 0.543-1.595 |
| TC | -0.123 | 0.507 | 0.059 | 0.809 | 0.885 | 0.328-2.388 |
| LDL | -0.310 | 0.643 | 0.233 | 0.629 | 0.733 | 0.208-2.584 |
| HDL | 1.221 | 1.393 | 0.769 | 0.381 | 3.392 | 0.221-52.034 |
| HbA1c | 0.164 | 0.489 | 0.113 | 0.737 | 1.178 | 0.452-3.070 |
| MBA | 0.040 | 0.049 | 0.681 | 0.409 | 1.041 | 0.946-1.146 |
| FD-RVN | -17.047 | 9.298 | 3.361 | 0.067 | 0.000 | 0.000-3.247 |
| MRVC(Caliber) | 0.091 | 0.061 | 2.250 | 0.134 | 1.095 | 0.972-1.233 |
| MRVC(Caliber)<sub>0.5–1.0PD</sub> | 0.006 | 0.043 | 0.016 | 0.898 | 1.006 | 0.924-1.094 |
| MRVC(Caliber)<sub>1.0–1.5PD</sub> | 0.051 | 0.048 | 1.129 | 0.288 | 1.052 | 0.958-1.156 |
| MRVC(Caliber)<sub>1.5–2.0PD</sub> | 0.063 | 0.052 | 1.444 | 0.230 | 1.065 | 0.961-1.180 |
| MRVC(Caliber)<sub>2.0–2.5PD</sub> | 0.002 | 0.044 | 0.002 | 0.968 | 1.002 | 0.918-1.093 |
| MRV(Vessel)T | -1664.398 | 3618.712 | 0.212 | 0.646 | 0.000 | 0.000 |
| MRVκ<sub>0.5–1.0PD</sub> | -494.112 | 2650.703 | 0.035 | 0.852 | 0.000 | 0.000 |
| MRVκ<sub>1.0–1.5PD</sub> | -246.928 | 3025.317 | 0.007 | 0.935 | 0.000 | 0.000 |
| MRVκ<sub>1.5–2.0PD</sub> | -751.732 | 2647.893 | 0.081 | 0.776 | 0.000 | 0.000 |
| MRVκ<sub>2.0–2.5PD</sub> | -1334.721 | 3749.627 | 0.127 | 0.722 | 0.000 | 0.000 |
| MRAT | 412.396 | 2996.242 | 0.019 | 0.891 | >100 | 0.000 |
| MRAC(Curvature)<sub>0.5-1.0PD</sub> | 116.817 | 1973.602 | 0.004 | 0.953 | >100 | 0.000 |
| MRAC(Curvature)<sub>1.0-1.5PD</sub> | -4162.756 | 2826.017 | 2.170 | 0.141 | 0.000 | 0.000 |
| MRAC(Curvature)<sub>1.5-2.0PD</sub> | -1771.992 | 2041.530 | 0.753 | 0.385 | 0.000 | 0.000 |
| MRAC(Curvature)<sub>2.0-2.5PD</sub> | 789.379 | 1145.350 | 0.475 | 0.491 | - | 0.000 |
| MRV(Venular)T | -1171.647 | 3044.040 | 0.148 | 0.700 | 0.000 | 0.000 |
| MRVC(Curvature)<sub>0.5-1.0PD</sub> | 381.976 | 1389.091 | 0.076 | 0.783 | >100 | 0.000 |
| MRVC(Curvature)<sub>1.0-1.5PD</sub> | 1212.484 | 2144.539 | 0.320 | 0.572 | - | 0.000 |
| MRVC(Curvature)<sub>1.5-2.0PD</sub> | -346.180 | 2130.160 | 0.026 | 0.871 | 0.000 | 0.000 |
| MRVC(Curvature)<sub>2.0-2.5PD</sub> | -3477.184 | 3795.509 | 0.839 | 0.360 | 0.000 | 0.000 |
| VD | -51.208 | 28.135 | 3.313 | 0.069 | 0.000 | 0.000-51.416 |
| MRAC | 0.113 | 0.076 | 2.195 | 0.139 | 1.119 | 0.964-1.300 |
| MRAC(Caliber)<sub>0.5-1.0PD</sub> | 0.038 | 0.050 | 0.600 | 0.438 | 1.039 | 0.943-1.145 |
| MRAC(Caliber)<sub>1.0-1.5PD</sub> | 0.007 | 0.043 | 0.027 | 0.869 | 1.007 | 0.925-1.097 |
| MRAC(Caliber)<sub>1.5-2.0PD</sub> | 0.056 | 0.058 | 0.929 | 0.335 | 1.057 | 0.944-1.184 |
| MRAC(Caliber)<sub>2.0-2.5PD</sub> | -0.046 | 0.066 | 0.476 | 0.490 | 0.955 | 0.839-1.088 |
| MRV(Venular)C | 0.061 | 0.052 | 1.386 | 0.239 | 1.063 | 0.960-1.176 |
| MRV(Venular)C<sub>0.5-1.0PD</sub> | 0.010 | 0.026 | 0.146 | 0.702 | 1.010 | 0.961-1.062 |
| MRV(Venular)C<sub>1.0-1.5PD</sub> | 0.009 | 0.045 | 0.040 | 0.842 | 1.009 | 0.924-1.101 |
| MRV(Venular)C<sub>1.5-2.0PD</sub> | -0.006 | 0.050 | 0.017 | 0.896 | 0.994 | 0.901-1.095 |
| MRV(Venular)C<sub>2.0-2.5PD</sub> | -0.020 | 0.033 | 0.353 | 0.553 | 0.981 | 0.919-1.046 |
| AVR | 5.041 | 7.746 | 0.424 | 0.515 | 154.626 | 0.000->100 |
| AVR <sub>0.5-1.0PD</sub> | 2.308 | 4.309 | 0.287 | 0.592 | 10.057 | 0.002-46773.597 |
| AVR <sub>1.0-1.5PD</sub> | -1.443 | 2.786 | 0.268 | 0.604 | 0.236 | 0.001-55.546 |
| AVR <sub>1.5-2.0PD</sub> | 8.212 | 7.164 | 1.314 | 0.252 | 3685.482 | 0.003->100 |
| AVR <sub>2.0-2.5PD</sub> | 1.705 | 6.142 | 0.077 | 0.781 | 5.501 | 0.000-929862.665 |
| DA | 0.000 | 0.000 | 0.013 | 0.908 | 1.000 | 1.000-1.000 |
| HDD | 0.002 | 0.002 | 1.292 | 0.256 | 1.002 | 0.999-1.005 |
| VDD | 0.002 | 0.002 | 0.818 | 0.366 | 1.002 | 0.998-1.005 |
| ODR | 0.132 | 14.972 | 0.000 | 0.993 | 1.142 | 0.000->100 |
| ODT (D) | 0.013 | 0.009 | 2.342 | 0.126 | 1.013 | 0.996-1.030 |
| MRW | 0.000 | 0.001 | 0.021 | 0.886 | 1.000 | 0.999-1.002 |
| DDM | 0.002 | 0.001 | 1.335 | 0.248 | 1.002 | 0.999-1.004 |
| ODAR | 0.986 | 8.375 | 0.014 | 0.906 | 2.679 | 0.000-36026201.940 |
| MFD | 0.000 | 0.000 | 0.015 | 0.903 | 1.000 | 0.999-1.001 |
| OCA | 0.000 | 0.000 | 0.186 | 0.666 | 1.000 | 1.000-1.000 |
| CHD | 0.000 | 0.000 | 0.149 | 0.700 | 1.000 | 1.000-1.000 |
| CVD | 0.000 | 0.000 | 0.157 | 0.692 | 1.000 | 1.000-1.000 |
| CIR-C | -4.322 | 12.767 | 0.115 | 0.735 | 0.013 | 0.000->100 |
| CDR-A | -0.947 | 6.114 | 0.024 | 0.877 | 0.388 | 0.000-62129.321 |
| HCDR | -0.630 | 5.506 | 0.013 | 0.909 | 0.533 | 0.000-25886.088 |
| VCDR | -0.170 | 0.651 | 0.069 | 0.793 | 0.843 | 0.236-3.020 |
| Rim-I | 0.000 | 0.005 | 0.000 | 0.999 | 1.000 | 0.991-1.010 |
| Rim-N | 0.002 | 0.004 | 0.362 | 0.547 | 1.002 | 0.995-1.010 |
| Rim-S | 0.001 | 0.005 | 0.054 | 0.817 | 1.001 | 0.992-1.010 |
| Rim-T | 0.002 | 0.004 | 0.311 | 0.577 | 1.002 | 0.995-1.009 |
| Max Rim-Cup D | 0.000 | 0.000 | 0.290 | 0.590 | 1.000 | 1.000-1.000 |
| Min Rim | 0.017 | 0.011 | 2.308 | 0.129 | 1.017 | 0.995-1.040 |

MBA, Mean Branching Angle; FD-RVN,Fractal Dimension of the Retinal Vascular Network; MRVC(Caliber) , Mean Retinal Vessel Caliber; MRVC(Caliber)<sub>0.5-1.0PD</sub>, Mean Retinal Vessel Caliber within 0.5-1.0 PD Zone; MRV(Vessel)T, Mean Retinal Vessel Tortuosity; MRVκ<sub>0.5-1.0PD</sub>, Mean Retinal Vessel Curvature within 0.5-1.0 PD Zone; MRAT, Mean Retinal Arteriolar Tortuosity; MRAC(Curvature)<sub>0.5-1.0PD</sub>, Mean Retinal Arteriolar Curvature within 0.5-1.0 PD Zone; MRV(Venular)T, Mean Retinal Venular Tortuosity; MRVC(Curvature)<sub>0.5-1.0PD</sub> ,Mean Retinal Venular Curvature within 0.5-1.0 PD Zone; VD, Vessel Density; MRAC, Mean Retinal Arteriolar Caliber ; MRAC(Caliber)<sub>0.5-1.0PD</sub> , Mean Retinal Arteriolar Caliber within 0.5-1.0 PD Zone; MRV(Venular)C, Mean Retinal Venular Caliber; MRV(Venular)C<sub>0.5-1.0PD</sub>, Mean Retinal Venular Caliber within 0.5-1.0 PD Zone; AVR, Arteriolar-to-Venular Ratio; DA, Optic Disc Area; HDD, Horizontal Diameter of the Optic Disc; VDD, Vertical Optic Disc Diameter; ODR, Optic Disc Roundness; ODT (D), Optic Disc Tilt; MRW, Minimum Rim Width; DDM, Disc-Macula Distance; ODAR, Optic Disc Axial Ratio; MFD, Macular Fovea Distance; OCA, Optic Cup Area; CHD, Cup Horizontal Diameter; CVD, Cup Vertical Diameter; CIR-C, Cup Circularity; CDR-A, Cup-to-Disc Area Ratio; HCDR, Horizontal Cup-to-Disc Ratio; VCDR, Vertical Cup-to-Disc Ratio; Rim-I, Inferior Neuroretinal Rim Width; Rim-N, Nasal Neuroretinal Rim Width; Rim-S, Superior Neuroretinal Rim Width; Rim-T, Temporal Neuroretinal Rim Width; Max Rim-Cup D, Maximum Rim-to-Cup Distance; Min Rim, Minimum Neuroretinal Rim Width; Results of univariate logistic regression are presented as odds ratios (OR) with 95% confidence intervals (CI). An OR > 1 indicates increased odds of the outcome, and an OR < 1 indicates decreased odds; P < 0.05 was considered significant.

Supplemental Table 14. Univariable logistic regression analysis of the PSM cohort for the T2DM with HTN group and the CHD with T2DM and HTN group.

|  | B | SE | Wald | *P* | OR | 95％*CI* |
| --- | --- | --- | --- | --- | --- | --- |
| Age | 0.006 | 0.025 | 0.063 | 0.802 | 1.006 | 0.958-1.057 |
| Sex |  |  |  |  |  |  |
| Male | 0.134 | 0.518 | 0.067 | 0.796 | 1.144 | 0.414-3.157 |
| Female | Ref. |  |  |  |  |  |
| Smoking history |  |  |  |  |  |  |
| No smoking | Ref. |  |  |  |  |  |
| Still smoking | 0.162 | 0.570 | 0.081 | 0.776 | 1.176 | 0.385-3.595 |
| Drinking history |  |  |  |  |  |  |
| No drinking | Ref. |  |  |  |  |  |
| Still drinking | 0.000 | 1.034 | 0.000 | 1.000 | 1.000 | 0.132-7.587 |
| FBG | -0.105 | 0.116 | 0.820 | 0.365 | 0.900 | 0.717-1.130 |
| Cr | -0.004 | 0.011 | 0.121 | 0.728 | 0.996 | 0.976-1.017 |
| Lp(a) | -0.001 | 0.010 | 0.013 | 0.908 | 0.999 | 0.979-1.019 |
| TG | -0.033 | 0.196 | 0.029 | 0.865 | 0.967 | 0.659-1.420 |
| TC | -0.059 | 0.252 | 0.055 | 0.815 | 0.943 | 0.576-1.544 |
| LDL | 0.014 | 0.272 | 0.003 | 0.958 | 1.015 | 0.595-1.729 |
| HDL | -0.394 | 0.773 | 0.260 | 0.610 | 0.674 | 0.148-3.066 |
| HbA1c | -0.403 | 0.303 | 1.762 | 0.184 | 0.669 | 0.369-1.211 |
| MBA | 0.044 | 0.024 | 3.481 | 0.062 | 1.045 | 0.998-1.095 |
| FD-RVN | 3.680 | 3.781 | 0.947 | 0.330 | 39.653 | 0.024-65625.643 |
| MRVC(Caliber) | -0.064 | 0.027 | 5.553 | 0.018 | 0.938 | 0.889-0.989 |
| MRVC(Caliber)<sub>0.5–1.0PD</sub> | -0.029 | 0.020 | 2.122 | 0.145 | 0.971 | 0.934-1.010 |
| MRVC(Caliber)<sub>1.0–1.5PD</sub> | -0.023 | 0.022 | 1.165 | 0.280 | 0.977 | 0.936-1.019 |
| MRVC(Caliber)<sub>1.5–2.0PD</sub> | -0.067 | 0.029 | 5.128 | 0.024 | 0.935 | 0.883-0.991 |
| MRVC(Caliber)<sub>2.0–2.5PD</sub> | -0.049 | 0.024 | 4.302 | 0.038 | 0.952 | 0.908-0.997 |
| MRV(Vessel)T | 2857.363 | 1764.600 | 2.622 | 0.105 | - | 0.000 |
| MRVκ<sub>0.5–1.0PD</sub> | -838.880 | 1231.043 | 0.464 | 0.496 | 0.000 | 0.000 |
| MRVκ<sub>1.0–1.5PD</sub> | 729.571 | 1218.267 | 0.359 | 0.549 | - | 0.000 |
| MRVκ<sub>1.5–2.0PD</sub> | 1592.813 | 1082.085 | 2.167 | 0.141 | - | 0.000 |
| MRVκ<sub>2.0–2.5PD</sub> | 2413.598 | 1244.528 | 3.761 | 0.052 | - | 0.000 |
| MRAT | 2377.283 | 1496.488 | 2.524 | 0.112 | - | 0.000 |
| MRAC(Curvature)<sub>0.5-1.0PD</sub> | 758.668 | 810.698 | 0.876 | 0.349 | - | 0.000 |
| MRAC(Curvature)<sub>1.0-1.5PD</sub> | 581.464 | 800.161 | 0.528 | 0.467 | >100 | 0.000 |
| MRAC(Curvature)<sub>1.5-2.0PD</sub> | 1721.263 | 1168.935 | 2.168 | 0.141 | - | 0.000 |
| MRAC(Curvature)<sub>2.0-2.5PD</sub> | 1332.491 | 1227.221 | 1.179 | 0.278 | - | 0.000 |
| MRV(Venular)T | 1842.206 | 1447.384 | 1.620 | 0.203 | - | 0.000 |
| MRVC(Curvature)<sub>0.5-1.0PD</sub> | -1527.144 | 1122.941 | 1.849 | 0.174 | 0.000 | 0.000->100 |
| MRVC(Curvature)<sub>1.0-1.5PD</sub> | 398.100 | 1094.424 | 0.132 | 0.716 | >100 | 0.000 |
| MRVC(Curvature)<sub>1.5-2.0PD</sub> | 1056.756 | 851.344 | 1.541 | 0.215 | - | 0.000 |
| MRVC(Curvature)<sub>2.0-2.5PD</sub> | 1537.593 | 930.817 | 2.729 | 0.099 | - | 0.000 |
| VD | 9.996 | 13.597 | 0.540 | 0.462 | 21935.862 | 0.000->100 |
| MRAC | -0.254 | 0.070 | 13.340 | <0.001 | 0.776 | 0.677-0.889 |
| MRAC(Caliber)<sub>0.5-1.0PD</sub> | -0.079 | 0.030 | 7.059 | 0.008 | 0.924 | 0.872-0.980 |
| MRAC(Caliber)<sub>1.0-1.5PD</sub> | -0.091 | 0.033 | 7.424 | 0.006 | 0.913 | 0.856-0.975 |
| MRAC(Caliber)<sub>1.5-2.0PD</sub> | -0.088 | 0.033 | 6.975 | 0.008 | 0.916 | 0.858-0.978 |
| MRAC(Caliber)<sub>2.0-2.5PD</sub> | -0.125 | 0.040 | 9.952 | 0.002 | 0.882 | 0.816-0.954 |
| MRV(Venular)C | -0.038 | 0.021 | 3.151 | 0.076 | 0.963 | 0.924-1.004 |
| MRV(Venular)C<sub>0.5-1.0PD</sub> | -0.028 | 0.017 | 2.770 | 0.096 | 0.972 | 0.941-1.005 |
| MRV(Venular)C<sub>1.0-1.5PD</sub> | -0.033 | 0.019 | 2.990 | 0.084 | 0.968 | 0.932-1.004 |
| MRV(Venular)C<sub>1.5-2.0PD</sub> | -0.060 | 0.026 | 5.445 | 0.020 | 0.942 | 0.896-0.990 |
| MRV(Venular)C<sub>2.0-2.5PD</sub> | -0.036 | 0.019 | 3.844 | 0.050 | 0.964 | 0.930-1.000 |
| AVR | -6.181 | 3.139 | 3.879 | 0.049 | 0.002 | 0.000-0.971 |
| AVR <sub>0.5-1.0PD</sub> | -2.847 | 2.622 | 1.179 | 0.277 | 0.058 | 0.000-9.887 |
| AVR <sub>1.0-1.5PD</sub> | -2.940 | 2.360 | 1.552 | 0.213 | 0.053 | 0.001-5.396 |
| AVR <sub>1.5-2.0PD</sub> | -3.338 | 2.196 | 2.311 | 0.129 | 0.035 | 0.000-2.628 |
| AVR <sub>2.0-2.5PD</sub> | -3.652 | 2.213 | 2.723 | 0.099 | 0.026 | 0.000-1.984 |
| DA | 0.000 | 0.000 | 2.718 | 0.099 | 1.000 | 1.000-1.000 |
| HDD | 0.000 | 0.001 | 0.019 | 0.891 | 1.000 | 0.999-1.002 |
| VDD | 0.001 | 0.001 | 0.440 | 0.507 | 1.001 | 0.999-1.002 |
| ODR | -5.054 | 3.847 | 1.726 | 0.189 | 0.006 | 0.000-12.021 |
| ODT (D) | -0.004 | 0.004 | 1.568 | 0.210 | 0.996 | 0.989-1.003 |
| MRW | 0.000 | 0.000 | 0.880 | 0.348 | 1.000 | 0.999-1.000 |
| DDM | -0.001 | 0.001 | 2.056 | 0.152 | 0.999 | 0.997-1.000 |
| ODAR | 3.322 | 2.496 | 1.771 | 0.183 | 27.702 | 0.208-3690.144 |
| MFD | 0.000 | 0.000 | 1.314 | 0.252 | 1.000 | 1.000-1.001 |
| OCA | 0.000 | 0.000 | 0.393 | 0.531 | 1.000 | 1.000-1.000 |
| CHD | 0.001 | 0.001 | 0.307 | 0.579 | 1.001 | 0.999-1.003 |
| CVD | 0.001 | 0.001 | 0.855 | 0.355 | 1.001 | 0.999-1.003 |
| CIR-C | -5.314 | 3.811 | 1.945 | 0.163 | 0.005 | 0.000-8.627 |
| CDR-A | 4.267 | 3.109 | 1.883 | 0.170 | 71.274 | 0.161-31567.359 |
| HCDR | 2.918 | 2.695 | 1.173 | 0.279 | 18.513 | 0.094-3643.486 |
| VCDR | 4.644 | 3.300 | 1.981 | 0.159 | 103.930 | 0.161-66895.092 |
| Rim-I | -0.001 | 0.003 | 0.102 | 0.750 | 0.999 | 0.993-1.005 |
| Rim-N | 0.000 | 0.002 | 0.000 | 0.994 | 1.000 | 0.995-1.004 |
| Rim-S | -0.004 | 0.003 | 2.374 | 0.123 | 0.996 | 0.990-1.001 |
| Rim-T | -0.005 | 0.002 | 5.537 | 0.019 | 0.995 | 0.990-0.999 |
| Max Rim-Cup D | 0.001 | 0.001 | 0.745 | 0.388 | 1.001 | 0.999-1.003 |
| Min Rim | -0.009 | 0.004 | 6.035 | 0.014 | 0.991 | 0.985-0.998 |

MBA, Mean Branching Angle; FD-RVN,Fractal Dimension of the Retinal Vascular Network; MRVC(Caliber) , Mean Retinal Vessel Caliber; MRVC(Caliber)<sub>0.5-1.0PD</sub>, Mean Retinal Vessel Caliber within 0.5-1.0 PD Zone; MRV(Vessel)T, Mean Retinal Vessel Tortuosity; MRVκ<sub>0.5-1.0PD</sub>, Mean Retinal Vessel Curvature within 0.5-1.0 PD Zone; MRAT, Mean Retinal Arteriolar Tortuosity; MRAC(Curvature)<sub>0.5-1.0PD</sub>, Mean Retinal Arteriolar Curvature within 0.5-1.0 PD Zone; MRV(Venular)T, Mean Retinal Venular Tortuosity; MRVC(Curvature)<sub>0.5-1.0PD</sub> ,Mean Retinal Venular Curvature within 0.5-1.0 PD Zone; VD, Vessel Density; MRAC, Mean Retinal Arteriolar Caliber ; MRAC(Caliber)<sub>0.5-1.0PD</sub> , Mean Retinal Arteriolar Caliber within 0.5-1.0 PD Zone; MRV(Venular)C, Mean Retinal Venular Caliber ; MRV(Venular)C<sub>0.5-1.0PD</sub>, Mean Retinal Venular Caliber within 0.5-1.0 PD Zone; AVR, Arteriolar-to-Venular Ratio; DA, Optic Disc Area; HDD, Horizontal Diameter of the Optic Disc; VDD, Vertical Optic Disc Diameter; ODR, Optic Disc Roundness; ODT (D), Optic Disc Tilt; MRW, Minimum Rim Width; DDM, Disc-Macula Distance; ODAR, Optic Disc Axial Ratio; MFD, Macular Fovea Distance; OCA, Optic Cup Area; CHD, Cup Horizontal Diameter; CVD, Cup Vertical Diameter; CIR-C, Cup Circularity; CDR-A, Cup-to-Disc Area Ratio; HCDR, Horizontal Cup-to-Disc Ratio; VCDR, Vertical Cup-to-Disc Ratio; Rim-I, Inferior Neuroretinal Rim Width; Rim-N, Nasal Neuroretinal Rim Width; Rim-S, Superior Neuroretinal Rim Width; Rim-T, Temporal Neuroretinal Rim Width; Max Rim-Cup D, Maximum Rim-to-Cup Distance; Min Rim, Minimum Neuroretinal Rim Width; Results of univariate logistic regression are presented as odds ratios (OR) with 95% confidence intervals (CI). An OR > 1 indicates increased odds of the outcome, and an OR < 1 indicates decreased odds; P < 0.05 was considered significant.

Supplemental Table 15. Multivariable logistic regression analysis of the primary cohort.

| Group | Variable | B | SE | Wald | *P* | OR | 95％*CI* |
| --- | --- | --- | --- | --- | --- | --- | --- |
| HTN and T2DM  VS  CHD with HTN and T2DM | Intercept | 12.011 | 3.153 | 14.516 | <0.001 | 164627.987 |  |
|  | Sex |  |  |  |  |  |  |
|  | Male | 1.379 | 0.481 | 8.226 | 0.004 | 3.972 | 1.548-10.195 |
|  | Female | Ref. |  |  |  |  |  |
|  | TC | -0.842 | 0.227 | 13.721 | <0.001 | 0.431 | 0.276-0.673 |
|  | HbA1c | 0.496 | 0.242 | 4.184 | 0.041 | 1.642 | 1.021-2.641 |
|  | MRAC | -0.156 | 0.046 | 11.652 | 0.001 | 0.855 | 0.782-0.936 |
|  | Rim-T | -0.007 | 0.003 | 6.003 | 0.014 | 0.993 | 0.988-0.999 |
|  |  |  |  |  |  |  |  |
| HTN  VS  CHD with HTN | Intercept | 65.666 | 13.674 | 23.063 | <0.001 | >100 |  |
|  | Age | 0.068 | 0.033 | 4.217 | 0.04 | 1.071 | 1.003-1.143 |
|  | MRAC | -1.175 | 0.263 | 20 | <0.001 | 0.309 | 0.185-0.517 |
|  | MRAC(Caliber)<sub>2.0-2.5PD</sub> | 0.179 | 0.081 | 4.879 | 0.027 | 1.196 | 1.02-1.402 |
|  | DDM | -0.002 | 0.001 | 3.633 | 0.057 | 0.998 | 0.996-1 |
|  |  |  |  |  |  |  |  |
| T2DM  VS  CHD with T2DM | Intercept | -15.672 | 8.135 | 3.711 | 0.054 | 0 |  |
|  | FD-RVN | 0.069 | 0.034 | 4.154 | 0.042 | 1.072 | 0-1.313 |
|  | MRV(Venular)C | -15.672 | 8.135 | 3.711 | 0.054 | 0 | 1.003-1.146 |
|  |  |  |  |  |  |  |  |
|  |  |  |  |  |  |  |  |
| Matched controls  VS  CHD | Intercept | 11.396 | 17.313 | 0.433 | 0.51 | 88995.682 |  |
|  | Sex |  |  |  |  |  |  |
|  | Male | 3.247 | 1.019 | 10.153 | 0.001 | 25.717 | 3.49-189.527 |
|  | Female | Ref. |  |  |  |  |  |
|  | FD-RVN | -25.67 | 10.309 | 6.201 | 0.013 | 0 | 0-0.004 |
|  | MRV(Venular)C | 0.406 | 0.098 | 17.033 | <0.001 | 1.5 | 1.237-1.819 |
|  | CIR-C | -18.307 | 9.129 | 4.022 | 0.045 | 0 | 0-0.660 |

MRAC, Mean Retinal Arteriolar Caliber; Rim-T, Temporal Neuroretinal Rim Width; MRAC(Caliber)<sub>2.0-2.5PD</sub> , Mean Retinal Arteriolar Caliber within 2.0-2.5PD Zone; DDM, Disc-Macula Distance; FD-RVN,Fractal Dimension of the Retinal Vascular Network; MRV(Venular)C, Mean Retinal Venular Caliber; CIR-C, Cup Circularity; A multivariable logistic regression model was constructed including all variables with P < 0.10 in univariate analysis. Results are presented as adjusted odds ratios (OR) with 95% confidence intervals (CI). P < 0.05 was considered significant.

Supplemental Table 16. Multivariable logistic regression analysis of the PSM cohort.

| Group | Variable | B | SE | Wald | *P* | OR | 95％*CI* |
| --- | --- | --- | --- | --- | --- | --- | --- |
| HTN and T2DM  VS  CHD with HTN and T2DM | Intercept | 15.194 | 4.126 | 13.564 | 0 | 3968572.224 |  |
|  | MRAC | -0.254 | 0.07 | 13.34 | <0.001 | 0.776 | 0.677-0.889 |
|  |  |  |  |  |  |  |  |
|  |  |  |  |  |  |  |  |
|  |  |  |  |  |  |  |  |
| HTN  VS  CHD with HTN | Intercept | 8921.602 | 100460.522 | 0.008 | 0.929 |  |  |
|  | MRAC(Curvature)<sub>2.0-2.5PD</sub> | 42429.917 | 655998.28 | 0.004 | 0.948 | - | 0.000 |
|  | MRAC | -167.983 | 1890.94 | 0.008 | 0.929 | 0 | 0.000 |
|  | MRAC(Caliber)<sub>2.0-2.5PD</sub> | 20.388 | 232.455 | 0.008 | 0.93 | 714958125.2 | 0.000->100 |
|  | ODT (D) | -0.592 | 7.087 | 0.007 | 0.933 | 0.553 | 0.000-596567.023 |
|  |  |  |  |  |  |  |  |
| Matched controls  VS  CHD | Intercept | 11.396 | 17.313 | 0.433 | 0.51 | 88995.682 |  |
|  | FD-RVN | -28.553 | 12.679 | 5.071 | 0.024 | 0 | 0-0.025 |
|  | MRV(Venular)C | 0.237 | 0.071 | 11.001 | 0.001 | 1.268 | 1.102-1.458 |
|  | MFD | -0.001 | 0.001 | 3.352 | 0.067 | 0.999 | 0.998-1 |
|  |  |  |  |  |  |  |  |

MRAC, Mean Retinal Arteriolar Caliber; MRAC(Curvature)<sub>2.0-2.5PD</sub>,Mean Retinal Arteriolar Curvature within 2.0-2.5PD Zone; MRAC(Caliber)<sub>2.0-2.5PD</sub> , Mean Retinal Arteriolar Caliber within 2.0-2.5PD Zone; ODT (D), Optic Disc Tilt; FD-RVN,Fractal Dimension of the Retinal Vascular Network; MRV(Venular)C, Mean Retinal Venular Caliber; MFD, Macular Fovea Distance; A multivariable logistic regression model was constructed including all variables with P < 0.10 in univariate analysis. Results are presented as adjusted odds ratios (OR) with 95% confidence intervals (CI). P < 0.05 was considered significant.

Supplemental Table 17. Univariate logistic regression analysis of short-term SGLT2 inhibitor use in the primary cohort of patients with T2DM and patients with CHD combined with T2DM.

|  | B | SE | Wald | *P* | OR | 95％*CI* |
| --- | --- | --- | --- | --- | --- | --- |
| Age | -0.013 | 0.026 | 0.260 | 0.610 | 0.987 | 0.937-1.039 |
| Sex |  |  |  |  |  |  |
| Male | -0.361 | 0.546 | 0.438 | 0.508 | 0.697 | 0.239-2.032 |
| Female | Ref. |  |  |  |  |  |
| Smoking history |  |  |  |  |  |  |
| No smoking | Ref. |  |  |  |  |  |
| Still smoking | 0.373 | 0.563 | 0.438 | 0.508 | 1.451 | 0.481-4.377 |
| Drinking history |  |  |  |  |  |  |
| No drinking | Ref. |  |  |  |  |  |
| Still drinking | -0.223 | 0.733 | 0.093 | 0.761 | 0.800 | 0.190-3.364 |
| CHD |  |  |  |  |  |  |
| NO | Ref. |  |  |  |  |  |
| YES | -0.373 | 0.563 | 0.438 | 0.508 | 0.689 | 0.228-2.078 |
| Aspirin, | 0.450 | 0.711 | 0.400 | 0.527 | 1.568 | 0.389-6.319 |
| Clopidogrel | -0.223 | 0.541 | 0.170 | 0.680 | 0.800 | 0.277-2.311 |
| Ticagrelor | -0.223 | 0.733 | 0.093 | 0.761 | 0.800 | 0.190-3.364 |
| Statins | -0.038 | 1.441 | 0.001 | 0.979 | 0.963 | 0.057-16.214 |
| ARNI, ACEI or ARB | 0.041 | 0.865 | 0.002 | 0.962 | 1.042 | 0.191-5.676 |
| β-Blockers | -0.511 | 0.544 | 0.882 | 0.348 | 0.600 | 0.207-1.742 |
| CCBs | 0.000 | 0.000 | 0.000 | 0.000 | 0.000 | 0.000 |
| Nitrates, | 0.769 | 0.609 | 1.593 | 0.207 | 2.157 | 0.654-7.116 |
| MRAs | 21.316 | 28420.721 | 0.000 | 0.999 | 1809331824.000 | 0.000 |
| FBG | -0.181 | 0.121 | 2.214 | 0.137 | 0.835 | 0.658-1.059 |
| Cr | 0.000 | 0.014 | 0.000 | 0.989 | 1.000 | 0.972-1.029 |
| Lp(a) | 0.007 | 0.010 | 0.427 | 0.514 | 1.007 | 0.986-1.028 |
| TG | -0.022 | 0.207 | 0.011 | 0.915 | 0.978 | 0.652-1.468 |
| TC | 0.058 | 0.219 | 0.072 | 0.789 | 1.060 | 0.691-1.628 |
| LDL | 0.206 | 0.270 | 0.584 | 0.445 | 1.229 | 0.724-2.085 |
| HDL | -1.066 | 0.849 | 1.575 | 0.209 | 0.344 | 0.065-1.820 |
| HbA1c | -0.098 | 0.273 | 0.130 | 0.719 | 0.906 | 0.531-1.548 |
| MBA | 0.025 | 0.023 | 1.115 | 0.291 | 1.025 | 0.979-1.073 |
| FD-RVN | -0.390 | 4.177 | 0.009 | 0.926 | 0.677 | 0.000-2432.308 |
| MRVC(Caliber) | -0.138 | 0.039 | 12.637 | 0.000 | 0.871 | 0.807-0.940 |
| MRVC(Caliber)<sub>0.5–1.0PD</sub> | -0.133 | 0.040 | 11.118 | 0.001 | 0.875 | 0.810-0.947 |
| MRVC(Caliber)<sub>1.0–1.5PD</sub> | -0.089 | 0.034 | 6.904 | 0.009 | 0.915 | 0.856-0.978 |
| MRVC(Caliber)<sub>1.5–2.0PD</sub> | -0.036 | 0.030 | 1.446 | 0.229 | 0.965 | 0.910-1.023 |
| MRVC(Caliber)<sub>2.0–2.5PD</sub> | -0.015 | 0.028 | 0.280 | 0.596 | 0.985 | 0.933-1.041 |
| MRV(Vessel)T | 7194.229 | 2465.644 | 8.513 | 0.004 | - | - |
| MRVκ<sub>0.5–1.0PD</sub> | 4114.072 | 1924.191 | 4.571 | 0.033 | - | >100 |
| MRVκ<sub>1.0–1.5PD</sub> | 3199.068 | 1843.423 | 3.012 | 0.083 | - | 0.000 |
| MRVκ<sub>1.5–2.0PD</sub> | 3572.011 | 1680.794 | 4.516 | 0.034 | - | >100 |
| MRVκ<sub>2.0–2.5PD</sub> | 2759.529 | 1755.125 | 2.472 | 0.116 | - | 0.000 |
| MRAT | 1846.769 | 1833.000 | 1.015 | 0.314 | - | 0.000 |
| MRAC(Curvature)<sub>0.5-1.0PD</sub> | 65.076 | 1313.998 | 0.002 | 0.961 | >100 | 0.000 |
| MRAC(Curvature)<sub>1.0-1.5PD</sub> | 325.859 | 1101.957 | 0.087 | 0.767 | >100 | 0.000 |
| MRAC(Curvature)<sub>1.5-2.0PD</sub> | -344.630 | 1009.789 | 0.116 | 0.733 | 0.000 | 0.000 |
| MRAC(Curvature)<sub>2.0-2.5PD</sub> | -231.701 | 825.149 | 0.079 | 0.779 | 0.000 | 0.000 |
| MRV(Venular)T | 6510.716 | 2157.682 | 9.105 | 0.003 | - | . |
| MRVC(Curvature)<sub>0.5-1.0PD</sub> | 3558.164 | 1700.361 | 4.379 | 0.036 | - | >100 |
| MRVC(Curvature)<sub>1.0-1.5PD</sub> | 2797.667 | 1441.772 | 3.765 | 0.052 | - | 0.000 |
| MRVC(Curvature)<sub>1.5-2.0PD</sub> | 2691.433 | 1244.845 | 4.675 | 0.031 | - | >100 |
| MRVC(Curvature)<sub>2.0-2.5PD</sub> | 2169.050 | 1417.346 | 2.342 | 0.126 | - | 0.000 |
| VD | -5.439 | 14.746 | 0.136 | 0.712 | 0.004 | 0.000->100 |
| MRAC | -0.349 | 0.094 | 13.958 | <0.001 | 0.705 | 0.587-0.847 |
| MRAC(Caliber)<sub>0.5-1.0PD</sub> | -0.213 | 0.061 | 12.355 | <0.001 | 0.808 | 0.718-0.910 |
| MRAC(Caliber)<sub>1.0-1.5PD</sub> | -0.115 | 0.039 | 8.605 | 0.003 | 0.892 | 0.826-0.963 |
| MRAC(Caliber)<sub>1.5-2.0PD</sub> | -0.122 | 0.044 | 7.519 | 0.006 | 0.885 | 0.811-0.966 |
| MRAC(Caliber)<sub>2.0-2.5PD</sub> | -0.075 | 0.037 | 4.061 | 0.044 | 0.928 | 0.862-0.998 |
| MRV(Venular)C | -0.087 | 0.028 | 9.400 | 0.002 | 0.917 | 0.867-0.969 |
| MRV(Venular)C<sub>0.5-1.0PD</sub> | -0.087 | 0.024 | 12.761 | <0.001 | 0.917 | 0.874-0.962 |
| MRV(Venular)C<sub>1.0-1.5PD</sub> | -0.053 | 0.025 | 4.497 | 0.034 | 0.948 | 0.902-0.996 |
| MRV(Venular)C<sub>1.5-2.0PD</sub> | -0.008 | 0.024 | 0.105 | 0.746 | 0.992 | 0.946-1.041 |
| MRV(Venular)C<sub>2.0-2.5PD</sub> | 0.020 | 0.020 | 1.025 | 0.311 | 1.020 | 0.981-1.060 |
| AVR | -8.849 | 4.536 | 3.806 | 0.051 | 0.000 | 0.000-1.042 |
| AVR <sub>0.5-1.0PD</sub> | -1.196 | 2.728 | 0.192 | 0.661 | 0.302 | 0.001-63.442 |
| AVR <sub>1.0-1.5PD</sub> | -1.629 | 2.328 | 0.490 | 0.484 | 0.196 | 0.002-18.795 |
| AVR <sub>1.5-2.0PD</sub> | -9.389 | 3.884 | 5.845 | 0.016 | 0.000 | 0.000-0.169 |
| AVR <sub>2.0-2.5PD</sub> | -8.233 | 3.362 | 5.996 | 0.014 | 0.000 | 0.000-0.193 |
| DA | 0.000 | 0.000 | 11.509 | 0.001 | 1.000 | 1.000-1.000 |
| HDD | -0.004 | 0.001 | 9.380 | 0.002 | 0.996 | 0.994-0.999 |
| VDD | -0.003 | 0.001 | 8.471 | 0.004 | 0.997 | 0.995-0.999 |
| ODR | -4.556 | 6.465 | 0.497 | 0.481 | 0.011 | 0.000-3343.498 |
| ODT (D) | -0.003 | 0.004 | 0.666 | 0.415 | 0.997 | 0.989-1.005 |
| MRW | 0.000 | 0.000 | 0.571 | 0.450 | 1.000 | 0.999-1.001 |
| DDM | -0.002 | 0.001 | 7.129 | 0.008 | 0.998 | 0.997-0.999 |
| ODAR | -2.253 | 4.159 | 0.293 | 0.588 | 0.105 | 0.000-367.567 |
| MFD | 0.001 | 0.000 | 3.355 | 0.067 | 1.001 | 1.000-1.001 |
| OCA | 0.000 | 0.000 | 7.336 | 0.007 | 1.000 | 1.000-1.000 |
| CHD | -0.003 | 0.001 | 5.513 | 0.019 | 0.997 | 0.995-1.000 |
| CVD | -0.002 | 0.001 | 5.033 | 0.025 | 0.998 | 0.995-1.000 |
| CIR-C | -0.335 | 0.674 | 0.247 | 0.619 | 0.716 | 0.191-2.680 |
| CDR-A | -4.328 | 3.199 | 1.830 | 0.176 | 0.013 | 0.000-6.975 |
| HCDR | -3.526 | 2.835 | 1.547 | 0.214 | 0.029 | 0.000-7.620 |
| VCDR | -3.799 | 3.143 | 1.461 | 0.227 | 0.022 | 0.000-10.600 |
| Rim-I | -0.006 | 0.003 | 3.534 | 0.060 | 0.994 | 0.988-1.000 |
| Rim-N | -0.001 | 0.003 | 0.140 | 0.709 | 0.999 | 0.994-1.004 |
| Rim-S | -0.004 | 0.003 | 1.803 | 0.179 | 0.996 | 0.990-1.002 |
| Rim-T | -0.005 | 0.002 | 3.959 | 0.047 | 0.995 | 0.990-1.000 |
| Max Rim-Cup D | -0.003 | 0.001 | 7.271 | 0.007 | 0.997 | 0.995-0.999 |
| Min Rim | 0.000 | 0.000 | 0.377 | 0.539 | 1.000 | 1.000-1.001 |

MBA, Mean Branching Angle; FD-RVN,Fractal Dimension of the Retinal Vascular Network; MRVC(Caliber) , Mean Retinal Vessel Caliber; MRVC(Caliber)<sub>0.5-1.0PD</sub>, Mean Retinal Vessel Caliber within 0.5-1.0 PD Zone; MRV(Vessel)T, Mean Retinal Vessel Tortuosity; MRVκ<sub>0.5-1.0PD</sub>, Mean Retinal Vessel Curvature within 0.5-1.0 PD Zone; MRAT, Mean Retinal Arteriolar Tortuosity; MRAC(Curvature)<sub>0.5-1.0PD</sub>, Mean Retinal Arteriolar Curvature within 0.5-1.0 PD Zone; MRV(Venular)T, Mean Retinal Venular Tortuosity; MRVC(Curvature)<sub>0.5-1.0PD</sub> ,Mean Retinal Venular Curvature within 0.5-1.0 PD Zone; VD, Vessel Density; MRAC, Mean Retinal Arteriolar Caliber ; MRAC(Caliber)<sub>0.5-1.0PD</sub> , Mean Retinal Arteriolar Caliber within 0.5-1.0 PD Zone; MRV(Venular)C, Mean Retinal Venular Caliber; MRV(Venular)C<sub>0.5-1.0PD</sub>, Mean Retinal Venular Caliber within 0.5-1.0 PD Zone; AVR, Arteriolar-to-Venular Ratio; DA, Optic Disc Area; HDD, Horizontal Diameter of the Optic Disc; VDD, Vertical Optic Disc Diameter; ODR, Optic Disc Roundness; ODT (D), Optic Disc Tilt; MRW, Minimum Rim Width; DDM, Disc-Macula Distance; ODAR, Optic Disc Axial Ratio; MFD, Macular Fovea Distance; OCA, Optic Cup Area; CHD, Cup Horizontal Diameter; CVD, Cup Vertical Diameter; CIR-C, Cup Circularity; CDR-A, Cup-to-Disc Area Ratio; HCDR, Horizontal Cup-to-Disc Ratio; VCDR, Vertical Cup-to-Disc Ratio; Rim-I, Inferior Neuroretinal Rim Width; Rim-N, Nasal Neuroretinal Rim Width; Rim-S, Superior Neuroretinal Rim Width; Rim-T, Temporal Neuroretinal Rim Width; Max Rim-Cup D, Maximum Rim-to-Cup Distance; Min Rim, Minimum Neuroretinal Rim Width; Results of univariate logistic regression are presented as odds ratios (OR) with 95% confidence intervals (CI). An OR > 1 indicates increased odds of the outcome, and an OR < 1 indicates decreased odds; P < 0.05 was considered significant.

Supplemental Table 18. Univariable logistic regression analysis of short-term SGLT2 inhibitor use in the primary cohort of T2DM with HTN patients and CHD with T2DM and HTN patients.

|  | B | SE | Wald | *P* | OR | 95％*CI* |
| --- | --- | --- | --- | --- | --- | --- |
| Age | -0.020 | 0.016 | 1.598 | 0.206 | 0.980 | 0.950-1.011 |
| Sex |  |  |  |  |  |  |
| Male | -0.401 | 0.325 | 1.520 | 0.218 | 0.670 | 0.354-1.267 |
| Female | Ref. |  |  |  |  |  |
| Smoking history |  |  |  |  |  |  |
| No smoking | Ref. |  |  |  |  |  |
| Still smoking | -0.659 | 0.319 | 4.267 | 0.039 | 0.517 | 0.277-0.967 |
| Drinking history |  |  |  |  |  |  |
| No drinking | Ref. |  |  |  |  |  |
| Still drinking | -0.580 | 0.387 | 2.246 | 0.134 | 0.560 | 0.262-1.196 |
| CHD |  |  |  |  |  |  |
| NO | Ref. |  |  |  |  |  |
| YES | -0.193 | 0.366 | 0.279 | 0.598 | 0.824 | 0.402-1.689 |
| Aspirin, | -0.572 | 0.516 | 1.228 | 0.268 | 0.565 | 0.205-1.552 |
| Clopidogrel | 0.358 | 0.318 | 1.265 | 0.261 | 1.431 | 0.766-2.670 |
| Ticagrelor | -0.281 | 0.387 | 0.529 | 0.467 | 0.755 | 0.354-1.611 |
| Statins | 0.124 | 0.690 | 0.032 | 0.857 | 1.132 | 0.293-4.379 |
| ARNI, ACEI or ARB | -0.351 | 0.343 | 1.044 | 0.307 | 0.704 | 0.359-1.380 |
| β-Blockers | 0.042 | 0.323 | 0.017 | 0.898 | 1.042 | 0.553-1.965 |
| CCBs | 0.441 | 0.321 | 1.890 | 0.169 | 1.555 | 0.829-2.916 |
| Nitrates, | -0.069 | 0.326 | 0.044 | 0.833 | 0.934 | 0.493-1.769 |
| MRAs | -1.548 | 0.687 | 5.077 | 0.024 | 0.213 | 0.055-0.818 |
| FBG | -0.084 | 0.060 | 1.969 | 0.161 | 0.919 | 0.817-1.034 |
| Cr | 0.000 | 0.002 | 0.036 | 0.849 | 1.000 | 0.996-1.005 |
| Lp(a) | -0.006 | 0.006 | 1.200 | 0.273 | 0.994 | 0.982-1.005 |
| TG | -0.029 | 0.077 | 0.142 | 0.706 | 0.971 | 0.835-1.130 |
| TC | 0.100 | 0.134 | 0.556 | 0.456 | 1.105 | 0.850-1.436 |
| LDL | 0.169 | 0.160 | 1.105 | 0.293 | 1.184 | 0.864-1.621 |
| HDL | 0.090 | 0.502 | 0.032 | 0.858 | 1.094 | 0.409-2.926 |
| HbA1c | -0.302 | 0.139 | 4.761 | 0.029 | 0.739 | 0.563-0.970 |
| MBA | 0.028 | 0.015 | 3.432 | 0.064 | 1.029 | 0.998-1.060 |
| FD-RVN | 7.074 | 2.307 | 9.400 | 0.002 | 1180.491 | 12.829-108623.130 |
| MRVC(Caliber) | -0.177 | 0.031 | 32.404 | 0.000 | 0.838 | 0.788-0.890 |
| MRVC(Caliber)<sub>0.5–1.0PD</sub> | -0.075 | 0.017 | 18.753 | 0.000 | 0.928 | 0.897-0.960 |
| MRVC(Caliber)<sub>1.0–1.5PD</sub> | -0.076 | 0.018 | 17.112 | 0.000 | 0.927 | 0.894-0.961 |
| MRVC(Caliber)<sub>1.5–2.0PD</sub> | -0.085 | 0.021 | 16.584 | 0.000 | 0.919 | 0.882-0.957 |
| MRVC(Caliber)<sub>2.0–2.5PD</sub> | -0.093 | 0.021 | 20.206 | 0.000 | 0.911 | 0.875-0.949 |
| MRV(Vessel)T | 518.271 | 951.041 | 0.297 | 0.586 | >100 | 0.000 |
| MRVκ<sub>0.5–1.0PD</sub> | -105.616 | 808.875 | 0.017 | 0.896 | 0.000 | 0.000 |
| MRVκ<sub>1.0–1.5PD</sub> | -612.559 | 675.641 | 0.822 | 0.365 | 0.000 | 0.000 |
| MRVκ<sub>1.5–2.0PD</sub> | 811.374 | 666.853 | 1.480 | 0.224 | . | 0.000 |
| MRVκ<sub>2.0–2.5PD</sub> | 505.953 | 691.019 | 0.536 | 0.464 | >100 | 0.000 |
| MRAT | -1106.105 | 964.834 | 1.314 | 0.252 | 0.000 | 0.000 |
| MRAC(Curvature)<sub>0.5-1.0PD</sub> | -8.811 | 584.487 | 0.000 | 0.988 | 0.000 | 0.000 |
| MRAC(Curvature)<sub>1.0-1.5PD</sub> | -1134.416 | 652.947 | 3.018 | 0.082 | 0.000 | 0.000->100 |
| MRAC(Curvature)<sub>1.5-2.0PD</sub> | -434.741 | 581.759 | 0.558 | 0.455 | 0.000 | 0.000->100 |
| MRAC(Curvature)<sub>2.0-2.5PD</sub> | -395.517 | 773.117 | 0.262 | 0.609 | 0.000 | 0.000 |
| MRV(Venular)T | 1371.776 | 825.129 | 2.764 | 0.096 | . | 0.000 |
| MRVC(Curvature)<sub>0.5-1.0PD</sub> | 190.683 | 659.806 | 0.084 | 0.773 | >100 | 0.000 |
| MRVC(Curvature)<sub>1.0-1.5PD</sub> | 132.007 | 548.782 | 0.058 | 0.810 | >100 | 0.000 |
| MRVC(Curvature)<sub>1.5-2.0PD</sub> | 1198.136 | 566.079 | 4.480 | 0.034 | . | >100 |
| MRVC(Curvature)<sub>2.0-2.5PD</sub> | 909.529 | 551.316 | 2.722 | 0.099 | . | 0.000 |
| VD | 25.214 | 8.370 | 9.075 | 0.003 | >100 | 6696.341->100 |
| MRAC | -0.049 | 0.025 | 3.932 | 0.047 | 0.952 | 0.907-0.999 |
| MRAC(Caliber)<sub>0.5-1.0PD</sub> | -0.018 | 0.017 | 1.036 | 0.309 | 0.982 | 0.949-1.017 |
| MRAC(Caliber)<sub>1.0-1.5PD</sub> | -0.010 | 0.017 | 0.350 | 0.554 | 0.990 | 0.958-1.023 |
| MRAC(Caliber)<sub>1.5-2.0PD</sub> | -0.010 | 0.017 | 0.336 | 0.562 | 0.990 | 0.959-1.023 |
| MRAC(Caliber)<sub>2.0-2.5PD</sub> | -0.015 | 0.016 | 0.937 | 0.333 | 0.985 | 0.955-1.016 |
| MRV(Venular)C | -0.320 | 0.051 | 38.921 | <0.001 | 0.726 | 0.656-0.803 |
| MRV(Venular)C<sub>0.5-1.0PD</sub> | -0.055 | 0.013 | 17.504 | <0.001 | 0.947 | 0.923-0.971 |
| MRV(Venular)C<sub>1.0-1.5PD</sub> | -0.079 | 0.017 | 22.476 | <0.001 | 0.924 | 0.894-0.955 |
| MRV(Venular)C<sub>1.5-2.0PD</sub> | -0.104 | 0.021 | 25.599 | <0.001 | 0.901 | 0.866-0.938 |
| MRV(Venular)C<sub>2.0-2.5PD</sub> | -0.059 | 0.015 | 16.090 | <0.001 | 0.942 | 0.916-0.970 |
| AVR | 13.088 | 2.646 | 24.470 | <0.001 | 482982.004 | 2703.200-86294607.060 |
| AVR <sub>0.5-1.0PD</sub> | 4.853 | 1.536 | 9.985 | 0.002 | 128.142 | 6.315-2600.104 |
| AVR <sub>1.0-1.5PD</sub> | 5.555 | 1.700 | 10.674 | 0.001 | 258.417 | 9.230-7235.389 |
| AVR <sub>1.5-2.0PD</sub> | 6.574 | 1.780 | 13.644 | <0.001 | 716.475 | 21.887-23453.502 |
| AVR <sub>2.0-2.5PD</sub> | 3.938 | 1.415 | 7.746 | 0.005 | 51.324 | 3.205-821.754 |
| DA | 0.000 | 0.000 | 4.863 | 0.027 | 1.000 | 1.000-1.000 |
| HDD | -0.002 | 0.001 | 8.167 | 0.004 | 0.998 | 0.997-0.999 |
| VDD | -0.001 | 0.001 | 3.327 | 0.068 | 0.999 | 0.997-1.000 |
| ODR | -1.530 | 2.096 | 0.533 | 0.465 | 0.216 | 0.004-13.159 |
| ODT (D) | -0.001 | 0.002 | 0.159 | 0.690 | 0.999 | 0.994-1.004 |
| MRW | -0.001 | 0.000 | 4.278 | 0.039 | 0.999 | 0.999-1.000 |
| DDM | 0.000 | 0.001 | 0.006 | 0.937 | 1.000 | 0.999-1.001 |
| ODAR | 0.736 | 1.148 | 0.411 | 0.521 | 2.088 | 0.220-19.820 |
| MFD | 0.001 | 0.000 | 6.813 | 0.009 | 1.001 | 1.000-1.001 |
| OCA | 0.000 | 0.000 | 6.419 | 0.011 | 1.000 | 1.000-1.000 |
| CHD | -0.002 | 0.001 | 4.939 | 0.026 | 0.998 | 0.997-1.000 |
| CVD | -0.001 | 0.001 | 3.711 | 0.054 | 0.999 | 0.997-1.000 |
| CIR-C | 3.217 | 2.418 | 1.771 | 0.183 | 24.962 | 0.219-2851.417 |
| CDR-A | -1.739 | 1.809 | 0.925 | 0.336 | 0.176 | 0.005-6.084 |
| HCDR | -0.931 | 1.561 | 0.356 | 0.551 | 0.394 | 0.019-8.397 |
| VCDR | -2.727 | 1.871 | 2.126 | 0.145 | 0.065 | 0.002-2.558 |
| Rim-I | 0.000 | 0.002 | 0.030 | 0.863 | 1.000 | 0.996-1.003 |
| Rim-N | -0.001 | 0.001 | 0.183 | 0.669 | 0.999 | 0.997-1.002 |
| Rim-S | -0.002 | 0.002 | 1.112 | 0.292 | 0.998 | 0.994-1.002 |
| Rim-T | -0.003 | 0.002 | 4.369 | 0.037 | 0.997 | 0.994-1.000 |
| Max Rim-Cup D | -0.002 | 0.001 | 6.459 | 0.011 | 0.998 | 0.997-1.000 |
| Min Rim | -0.003 | 0.002 | 1.943 | 0.163 | 0.997 | 0.993-1.001 |

MBA, Mean Branching Angle; FD-RVN,Fractal Dimension of the Retinal Vascular Network; MRVC(Caliber) , Mean Retinal Vessel Caliber; MRVC(Caliber)<sub>0.5-1.0PD</sub>, Mean Retinal Vessel Caliber within 0.5-1.0 PD Zone; MRV(Vessel)T, Mean Retinal Vessel Tortuosity; MRVκ<sub>0.5-1.0PD</sub>, Mean Retinal Vessel Curvature within 0.5-1.0 PD Zone; MRAT, Mean Retinal Arteriolar Tortuosity; MRAC(Curvature)<sub>0.5-1.0PD</sub>, Mean Retinal Arteriolar Curvature within 0.5-1.0 PD Zone; MRV(Venular)T, Mean Retinal Venular Tortuosity; MRVC(Curvature)<sub>0.5-1.0PD</sub> ,Mean Retinal Venular Curvature within 0.5-1.0 PD Zone; VD, Vessel Density; MRAC, Mean Retinal Arteriolar Caliber ; MRAC(Caliber)<sub>0.5-1.0PD</sub> , Mean Retinal Arteriolar Caliber within 0.5-1.0 PD Zone; MRV(Venular)C, Mean Retinal Venular Caliber; MRV(Venular)C<sub>0.5-1.0PD</sub>, Mean Retinal Venular Caliber within 0.5-1.0 PD Zone; AVR, Arteriolar-to-Venular Ratio; DA, Optic Disc Area; HDD, Horizontal Diameter of the Optic Disc; VDD, Vertical Optic Disc Diameter; ODR, Optic Disc Roundness; ODT (D), Optic Disc Tilt; MRW, Minimum Rim Width; DDM, Disc-Macula Distance; ODAR, Optic Disc Axial Ratio; MFD, Macular Fovea Distance; OCA, Optic Cup Area; CHD, Cup Horizontal Diameter; CVD, Cup Vertical Diameter; CIR-C, Cup Circularity; CDR-A, Cup-to-Disc Area Ratio; HCDR, Horizontal Cup-to-Disc Ratio; VCDR, Vertical Cup-to-Disc Ratio; Rim-I, Inferior Neuroretinal Rim Width; Rim-N, Nasal Neuroretinal Rim Width; Rim-S, Superior Neuroretinal Rim Width; Rim-T, Temporal Neuroretinal Rim Width; Max Rim-Cup D, Maximum Rim-to-Cup Distance; Min Rim, Minimum Neuroretinal Rim Width; Results of univariate logistic regression are presented as odds ratios (OR) with 95% confidence intervals (CI). An OR > 1 indicates increased odds of the outcome, and an OR < 1 indicates decreased odds; P < 0.05 was considered significant.

Supplemental Table 19. Univariable logistic regression analysis of short-term SGLT2 inhibitor use in the PSM cohort of T2DM with HTN patients and CHD with T2DM and HTN patients.

|  | B | SE | Wald | *P* | OR | 95％*CI* |
| --- | --- | --- | --- | --- | --- | --- |
| Age | -0.001 | 0.020 | 0.005 | 0.943 | 0.999 | 0.960-1.039 |
| Sex |  |  |  |  |  |  |
| Male | -0.294 | 0.384 | 0.585 | 0.445 | 0.746 | 0.351-1.582 |
| Female | Ref. |  |  |  |  |  |
| Smoking history |  |  |  |  |  |  |
| No smoking | Ref. |  |  |  |  |  |
| Still smoking | -0.485 | 0.374 | 1.682 | 0.195 | 0.616 | 0.296-1.282 |
| Drinking history |  |  |  |  |  |  |
| No drinking | Ref. |  |  |  |  |  |
| Still drinking | -0.399 | 0.450 | 0.788 | 0.375 | 0.671 | 0.278-1.619 |
| CHD |  |  |  |  |  |  |
| NO | Ref. |  |  |  |  |  |
| YES | 0.000 | 0.424 | 0.000 | 1.000 | 1.000 | 0.436-2.296 |
| Aspirin, | -0.816 | 0.539 | 2.287 | 0.130 | 0.442 | 0.154-1.273 |
| Clopidogrel | 0.990 | 0.384 | 6.652 | 0.010 | 2.692 | 1.268-5.712 |
| Ticagrelor | -1.021 | 0.499 | 4.189 | 0.041 | 0.360 | 0.136-0.958 |
| Statins | 0.000 | 0.838 | 0.000 | 1.000 | 1.000 | 0.193-5.173 |
| ARNI, ACEI or ARB | -0.082 | 0.405 | 0.041 | 0.840 | 0.921 | 0.417-2.037 |
| β-Blockers | -0.075 | 0.389 | 0.038 | 0.846 | 0.927 | 0.433-1.986 |
| CCBs | 0.566 | 0.379 | 2.234 | 0.135 | 1.762 | 0.838-3.703 |
| Nitrates, | 0.000 | 0.383 | 0.000 | 1.000 | 1.000 | 0.472-2.117 |
| MRAs | -21.351 | 14210.361 | 0.000 | 0.999 | 0.000 | 0.000 |
| FBG | 0.006 | 0.068 | 0.008 | 0.930 | 1.006 | 0.881-0.149 |
| Cr | 0.001 | 0.003 | 0.283 | 0.595 | 1.001 | 0.996-1.007 |
| Lp(a) | -0.001 | 0.006 | 0.007 | 0.932 | 0.999 | 0.987-1.012 |
| TG | 0.181 | 0.146 | 1.524 | 0.217 | 1.198 | 0.899-1.597 |
| TC | -0.009 | 0.163 | 0.003 | 0.954 | 0.991 | 0.720-1.364 |
| LDL | -0.105 | 0.190 | 0.307 | 0.579 | 0.900 | 0.621-1.305 |
| HDL | -0.078 | 0.563 | 0.019 | 0.890 | 0.925 | 0.307-2.791 |
| HbA1c | -0.054 | 0.170 | 0.099 | 0.753 | 0.948 | 0.679-1.324 |
| MBA | 0.021 | 0.018 | 1.372 | 0.241 | 1.021 | 0.986-1.057 |
| FD-RVN | 5.346 | 2.656 | 4.053 | 0.044 | 209.802 | 1.152-38212.363 |
| MRVC(Caliber) | -0.172 | 0.037 | 22.140 | 0.000 | 0.842 | 0.783-0.904 |
| MRVC(Caliber)<sub>0.5–1.0PD</sub> | -0.066 | 0.021 | 10.238 | 0.001 | 0.936 | 0.899-0.975 |
| MRVC(Caliber)<sub>1.0–1.5PD</sub> | -0.075 | 0.024 | 10.151 | 0.001 | 0.928 | 0.886-0.971 |
| MRVC(Caliber)<sub>1.5–2.0PD</sub> | -0.080 | 0.025 | 10.258 | 0.001 | 0.923 | 0.879-0.969 |
| MRVC(Caliber)<sub>2.0–2.5PD</sub> | -0.080 | 0.024 | 11.122 | 0.001 | 0.923 | 0.881-0.967 |
| MRV(Vessel)T | 92.361 | 1024.667 | 0.008 | 0.928 | >100 | 0.000 |
| MRVκ<sub>0.5–1.0PD</sub> | -862.374 | 950.030 | 0.824 | 0.364 | 0.000 | 0.000 |
| MRVκ<sub>1.0–1.5PD</sub> | -571.698 | 752.442 | 0.577 | 0.447 | 0.000 | 0.000 |
| MRVκ<sub>1.5–2.0PD</sub> | 430.261 | 722.737 | 0.354 | 0.552 | >100 | 0.000 |
| MRVκ<sub>2.0–2.5PD</sub> | 39.484 | 791.705 | 0.002 | 0.960 | >100 | 0.000 |
| MRAT | -1550.463 | 1108.639 | 1.956 | 0.162 | 0.000 | 0.000->100 |
| MRAC(Curvature)<sub>0.5-1.0PD</sub> | -1153.985 | 791.308 | 2.127 | 0.145 | 0.000 | 0.000->100 |
| MRAC(Curvature)<sub>1.0-1.5PD</sub> | -1405.338 | 803.214 | 3.061 | 0.080 | 0.000 | 0.000->100 |
| MRAC(Curvature)<sub>1.5-2.0PD</sub> | -829.941 | 712.459 | 1.357 | 0.244 | 0.000 | 0.000->100 |
| MRAC(Curvature)<sub>2.0-2.5PD</sub> | -654.267 | 921.585 | 0.504 | 0.478 | 0.000 | 0.000 |
| MRV(Venular)T | 901.480 | 869.034 | 1.076 | 0.300 | - | 0.000 |
| MRVC(Curvature)<sub>0.5-1.0PD</sub> | -239.634 | 753.446 | 0.101 | 0.750 | 0.000 | 0.000 |
| MRVC(Curvature)<sub>1.0-1.5PD</sub> | 233.079 | 617.687 | 0.142 | 0.706 | >100 | 0.000 |
| MRVC(Curvature)<sub>1.5-2.0PD</sub> | 1079.226 | 634.080 | 2.897 | 0.089 | - | 0.000 |
| MRVC(Curvature)<sub>2.0-2.5PD</sub> | 613.785 | 640.838 | 0.917 | 0.338 | >100 | 0.000 |
| VD | 21.063 | 9.792 | 4.627 | 0.031 | >100 | 6.497->100 |
| MRAC | -0.064 | 0.031 | 4.280 | 0.039 | 0.938 | 0.883-0.997 |
| MRAC(Caliber)<sub>0.5-1.0PD</sub> | -0.007 | 0.020 | 0.110 | 0.741 | 0.993 | 0.955-1.033 |
| MRAC(Caliber)<sub>1.0-1.5PD</sub> | -0.019 | 0.021 | 0.887 | 0.346 | 0.981 | 0.942-1.021 |
| MRAC(Caliber)<sub>1.5-2.0PD</sub> | -0.018 | 0.020 | 0.781 | 0.377 | 0.982 | 0.944-1.022 |
| MRAC(Caliber)<sub>2.0-2.5PD</sub> | -0.027 | 0.021 | 1.681 | 0.195 | 0.973 | 0.935-1.014 |
| MRV(Venular)C | -0.312 | 0.059 | 28.369 | <0.001 | 0.732 | 0.653-0.821 |
| MRV(Venular)C<sub>0.5-1.0PD</sub> | -0.042 | 0.015 | 7.441 | 0.006 | 0.959 | 0.930-0.988 |
| MRV(Venular)C<sub>1.0-1.5PD</sub> | -0.077 | 0.020 | 14.229 | <0.001 | 0.926 | 0.890-0.964 |
| MRV(Venular)C<sub>1.5-2.0PD</sub> | -0.082 | 0.023 | 12.951 | <0.001 | 0.921 | 0.881-0.963 |
| MRV(Venular)C<sub>2.0-2.5PD</sub> | -0.045 | 0.016 | 7.728 | 0.005 | 0.956 | 0.926-0.987 |
| AVR | 12.332 | 3.188 | 14.962 | <0.001 | 226847.208 | 438.507->100 |
| AVR <sub>0.5-1.0PD</sub> | 4.125 | 1.804 | 5.228 | 0.022 | 61.896 | 1.803-2125.410 |
| AVR <sub>1.0-1.5PD</sub> | 4.318 | 1.874 | 5.306 | 0.021 | 75.003 | 1.904-2954.669 |
| AVR <sub>1.5-2.0PD</sub> | 4.561 | 2.010 | 5.147 | 0.023 | 95.665 | 1.860-4919.231 |
| AVR <sub>2.0-2.5PD</sub> | 2.887 | 1.652 | 3.055 | 0.081 | 17.938 | 0.704-456.864 |
| DA | 0.000 | 0.000 | 3.121 | 0.077 | 1.000 | 1.000-1.000 |
| HDD | -0.003 | 0.001 | 9.334 | 0.002 | 0.997 | 0.995-0.999 |
| VDD | -0.001 | 0.001 | 2.917 | 0.088 | 0.999 | 0.997-1.000 |
| ODR | -3.149 | 2.601 | 1.466 | 0.226 | 0.043 | 0.000-7.019 |
| ODT (D) | 0.001 | 0.003 | 0.035 | 0.852 | 1.001 | 0.995-1.006 |
| MRW | -0.001 | 0.000 | 4.746 | 0.029 | 0.999 | 0.999-1.000 |
| DDM | -0.001 | 0.001 | 0.897 | 0.344 | 0.999 | 0.998-1.001 |
| ODAR | 2.264 | 1.571 | 2.076 | 0.150 | 9.621 | 0.442-209.239 |
| MFD | 0.001 | 0.000 | 4.715 | 0.030 | 1.001 | 1.000-1.002 |
| OCA | 0.000 | 0.000 | 4.653 | 0.031 | 1.000 | 1.000-1.000 |
| CHD | -0.002 | 0.001 | 5.366 | 0.021 | 0.998 | 0.996-1.000 |
| CVD | -0.002 | 0.001 | 4.998 | 0.025 | 0.998 | 0.996-1.000 |
| CIR-C | 2.088 | 2.728 | 0.586 | 0.444 | 8.069 | 0.038-1693.689 |
| CDR-A | -3.357 | 2.264 | 2.199 | 0.138 | 0.035 | 0.000-2.946 |
| HCDR | -1.705 | 1.920 | 0.789 | 0.374 | 0.182 | 0.004-7.826 |
| VCDR | -4.752 | 2.304 | 4.253 | 0.039 | 0.009 | 0.000-0.790 |
| Rim-I | -0.001 | 0.002 | 0.121 | 0.727 | 0.999 | 0.995-1.003 |
| Rim-N | -0.002 | 0.002 | 0.998 | 0.318 | 0.998 | 0.995-1.002 |
| Rim-S | -0.001 | 0.002 | 0.111 | 0.739 | 0.999 | 0.995-1.003 |
| Rim-T | -0.003 | 0.002 | 2.460 | 0.117 | 0.997 | 0.993-1.001 |
| Max Rim-Cup D | -0.002 | 0.001 | 6.479 | 0.011 | 0.998 | 0.996-0.999 |
| Min Rim | -0.002 | 0.003 | 0.513 | 0.474 | 0.998 | 0.993-1.003 |

MBA, Mean Branching Angle; FD-RVN,Fractal Dimension of the Retinal Vascular Network; MRVC(Caliber) , Mean Retinal Vessel Caliber; MRVC(Caliber)<sub>0.5-1.0PD</sub>, Mean Retinal Vessel Caliber within 0.5-1.0 PD Zone; MRV(Vessel)T, Mean Retinal Vessel Tortuosity; MRVκ<sub>0.5-1.0PD</sub>, Mean Retinal Vessel Curvature within 0.5-1.0 PD Zone; MRAT, Mean Retinal Arteriolar Tortuosity; MRAC(Curvature)<sub>0.5-1.0PD</sub>, Mean Retinal Arteriolar Curvature within 0.5-1.0 PD Zone; MRV(Venular)T, Mean Retinal Venular Tortuosity; MRVC(Curvature)<sub>0.5-1.0PD</sub> ,Mean Retinal Venular Curvature within 0.5-1.0 PD Zone; VD, Vessel Density; MRAC, Mean Retinal Arteriolar Caliber ; MRAC(Caliber)<sub>0.5-1.0PD</sub> , Mean Retinal Arteriolar Caliber within 0.5-1.0 PD Zone; MRV(Venular)C, Mean Retinal Venular Caliber; MRV(Venular)C<sub>0.5-1.0PD</sub>, Mean Retinal Venular Caliber within 0.5-1.0 PD Zone; AVR, Arteriolar-to-Venular Ratio; DA, Optic Disc Area; HDD, Horizontal Diameter of the Optic Disc; VDD, Vertical Optic Disc Diameter; ODR, Optic Disc Roundness; ODT (D), Optic Disc Tilt; MRW, Minimum Rim Width; DDM, Disc-Macula Distance; ODAR, Optic Disc Axial Ratio; MFD, Macular Fovea Distance; OCA, Optic Cup Area; CHD, Cup Horizontal Diameter; CVD, Cup Vertical Diameter; CIR-C, Cup Circularity; CDR-A, Cup-to-Disc Area Ratio; HCDR, Horizontal Cup-to-Disc Ratio; VCDR, Vertical Cup-to-Disc Ratio; Rim-I, Inferior Neuroretinal Rim Width; Rim-N, Nasal Neuroretinal Rim Width; Rim-S, Superior Neuroretinal Rim Width; Rim-T, Temporal Neuroretinal Rim Width; Max Rim-Cup D, Maximum Rim-to-Cup Distance; Min Rim, Minimum Neuroretinal Rim Width; Results of univariate logistic regression are presented as odds ratios (OR) with 95% confidence intervals (CI). An OR > 1 indicates increased odds of the outcome, and an OR < 1 indicates decreased odds; P < 0.05 was considered significant.

Supplemental Table 20. Subgroup analysis of short-term SGLT2 inhibitor use in the primary cohort of patients with T2DM and HTN and patients with CHD, T2DM, and HTN.

| CHD(N/Y) | Variable | B | SE | Wald | *P* | OR | 95％*CI* |
| --- | --- | --- | --- | --- | --- | --- | --- |
| No | Intercept | 228.368 | 256.073 | 0.795 | 0.372 | >100 |  |
|  | MRV(Venular)C | -2.521 | 2.819 | 0.800 | 0.371 | 0.080 | 0.000-20.162 |
| Yes | Intercept | 42.597 | 9.505 | 20.083 | 0.000 | >100 |  |
|  | Ticagrelor | -2.354 | 0.834 | 7.970 | 0.005 | 0.095 | 0.019-0.487 |
|  | MRAC(Curvature)<sub>2.0-2.5PD</sub> | -8784.237 | 2411.214 | 13.272 | 0.000 | 0.000 | 0.000 |
|  | MRV(Venular)C | -0.538 | 0.112 | 23.208 | <0.001 | 0.584 | 0.469-0.727 |
|  | AVR <sub>1.5-2.0PD</sub> | 14.395 | 4.848 | 8.817 | 0.003 | 1785742.893 | 133.400->100 |

MRV(Venular)C, Mean Retinal Venular Caliber; MRAC(Curvature)<sub>2.0-2.5PD</sub>, Mean Retinal Arteriolar Curvature within 2.0-2.5 PD Zone; AVR<sub>1.5-2.0 PD</sub>, AVR within 1.5-2.0 PD Zone; Results of subgroup analysis are presented as odds ratios (OR) with 95% confidence intervals (CI). An OR > 1 indicates increased odds of the outcome, and an OR < 1 indicates decreased odds; P < 0.05 was considered significant.

Supplemental Table 21. Subgroup analysis of AMI patients with short-term SGLT2 inhibitor use in the primary cohort of CHD with HTN and T2DM.

| AMI(N/Y) | Variable | B | SE | Wald | *P* | OR | 95％*CI* |
| --- | --- | --- | --- | --- | --- | --- | --- |
| No | Intercept | 220.433 | 192.269 | 1.314 | 0.252 | >100 |  |
|  | Drinking history (Still drinking) | -15.490 | 14.104 | 1.206 | 0.272 | 0.000 | 0.000-189922.814 |
|  | Ticagrelor (Y) | -26.449 | 113.819 | 0.054 | 0.816 | 0.000 | 0.000->100 |
|  | MRV(Venular)C | -2.487 | 2.171 | 1.312 | 0.252 | 0.083 | 0.001-5.861 |
| Yes | Intercept | 51.449 | 11.689 | 19.373 | 0.000 | >100 |  |
|  | CHD(Y) | -2.104 | 1.043 | 4.070 | 0.044 | 0.122 | 0.016-0.942 |
|  | Sex (male) | -1.765 | 0.812 | 4.729 | 0.030 | 0.171 | 0.035-0.840 |
|  | MRAC(Curvature)<sub>2.0-2.5PD</sub> | -5311.746 | 1710.419 | 9.644 | 0.002 | 0.000 | 0.000 |
|  | MRV(Venular)C | -0.596 | 0.128 | 21.810 | <0.001 | 0.551 | 0.429-0.708 |
|  | AVR <sub>1.5-2.0PD</sub> | 9.311 | 4.135 | 5.071 | 0.024 | 11058.482 | 3.345-36562935.790 |

MRV(Venular)C, Mean Retinal Venular Caliber; MRAC(Curvature)<sub>2.0-2.5PD</sub>, Mean Retinal Arteriolar Curvature within 2.0-2.5 PD Zone; AVR<sub>1.5-2.0 PD</sub>, AVR within 1.5-2.0 PD Zone; Results of subgroup analysis are presented as odds ratios (OR) with 95% confidence intervals (CI). An OR > 1 indicates increased odds of the outcome, and an OR < 1 indicates decreased odds; P < 0.05 was considered significant.

Supplemental Table 22. Subgroup analysis of AMI patients with short-term SGLT2 inhibitor use in the primary cohort of CHD with T2DM.

| AMI(N/Y) | Variable | B | SE | Wald | *P* | OR | 95％*CI* |
| --- | --- | --- | --- | --- | --- | --- | --- |
| No | Intercept | 284.727 | 107671.644 | 0.000 | 0.998 | >100 |  |
|  | MRAC | -7.253 | 2883.425 | 0.000 | 0.998 | 0.001 | 0.000 |
|  | MRAC(Caliber)<sub>2.0-2.5PD</sub> | 2.792 | 1524.589 | 0.000 | 0.999 | 16.314 | 0.000 |
|  |  |  |  |  |  |  |  |
| Yes | Intercept | 594.619 | 48109.351 | 0.000 | 0.990 | >100 | 0.000 |
|  | Ticagrelor (Y) | -77.611 | 5703.580 | 0.000 | 0.989 | 0.000 | 0.000 |
|  | TG | 46.728 | 3572.015 | 0.000 | 0.990 | >100 | 0.000 |
|  | MRAC(Curvature)<sub>0.5-1.0PD</sub> | -61821.100 | 6413221.809 | 0.000 | 0.992 | 0.000 | 0.000 |
|  | MRAC | -20.018 | 1331.162 | 0.000 | 0.988 | 0.000 | 0.000 |
|  | MRV(Venular)C<sub>2.0-2.5PD</sub> | 7.379 | 516.488 | 0.000 | 0.989 | 1601.429 | 0.000 |

MRAC, Mean Retinal Arteriolar Caliber ; MRAC(Caliber)<sub>2.0-2.5PD</sub> , Mean Retinal Arteriolar Caliber within 2.0-2.5 PD Zone; MRAC(Curvature)<sub>0.5-1.0PD</sub>, Mean Retinal Arteriolar Curvature within 0.5-1.0 PD Zone; MRV(Venular)C<sub>2.0-2.5PD</sub>, Mean Retinal Venular Caliber within 2.0-2.5 PD Zone; Results of subgroup analysis are presented as odds ratios (OR) with 95% confidence intervals (CI). An OR > 1 indicates increased odds of the outcome, and an OR < 1 indicates decreased odds; P < 0.05 was considered significant.

Supplemental Table 23. Multivariable logistic regression analysis of short-term SGLT2 inhibitor use in the primary cohort.

| Group | Variable | B | SE | Wald | *P* | OR | 95％*CI* |
| --- | --- | --- | --- | --- | --- | --- | --- |
| HTN and T2DM  VS  CHD with HTN and T2DM | Intercept | 41.687 | 7.987 | 27.243 | 0.000 | >100 |  |
|  | Smoking history |  |  |  |  |  |  |
|  | No smoking | Ref. |  |  |  |  |  |
|  | Still smoking | -1.166 | 0.651 | 3.206 | 0.073 | 0.312 | 0.087-1.117 |
|  | Ticagrelor | -1.809 | 0.756 | 5.718 | 0.017 | 0.164 | 0.037-0.722 |
|  | TC | 0.850 | 0.313 | 7.363 | 0.007 | 2.339 | 1.266-4.322 |
|  | MRAC(Curvature)<sub>2.0-2.5PD</sub> | -7046.750 | 2049.665 | 11.820 | 0.001 | 0.000 | 0.000 |
|  | MRV(Venular)C | -0.639 | 0.109 | 34.314 | <0.001 | 0.528 | 0.426-0.654 |
|  | MRV(Venular)C<sub>2.0-2.5PD</sub> | 0.084 | 0.032 | 6.854 | 0.009 | 1.088 | 1.021-1.158 |
|  | AVR <sub>1.5-2.0PD</sub> | 11.839 | 3.990 | 8.805 | 0.003 | 138537.642 | 55.646->100 |
|  |  |  |  |  |  |  |  |
| T2DM  VS  CHD with T2DM | Intercept | 1078.056 | 50787.029 | 0.000 | 0.983 | - |  |
|  | HDL | -122.651 | 6467.271 | 0.000 | 0.985 | 0.000 | 0.000 |
|  | MRVC(Caliber)<sub>1.5–2.0PD</sub> | 12.042 | 533.036 | 0.001 | 0.982 | 169721.407 | 0.000 |
|  | MRAC | -40.745 | 1802.069 | 0.001 | 0.982 | 0.000 | 0.000 |
|  | ODR | 473.021 | 28211.305 | 0.000 | 0.987 | >100 | 0.000 |
|  | MRW | 0.109 | 5.269 | 0.000 | 0.983 | 1.116 | 0.000-34061.256 |
|  |  |  |  |  |  |  |  |

MRAC(Curvature)<sub>2.0-2.5PD</sub>, Mean Retinal Arteriolar Curvature within 2.0-2.5 PD Zone; MRV(Venular)C, Mean Retinal Venular Caliber; MRV(Venular)C<sub>2.0-2.5PD</sub>, Mean Retinal Venular Caliber within 2.0-2.5 PD Zone; AVR<sub>1.5-2.0 PD</sub>, AVR within 1.5-2.0 PD Zone; MRVC(Caliber)<sub>1.5-2.0 PD</sub>, Mean Retinal Vessel Caliber within 1.5-2.0 PD Zone; MRAC, Mean Retinal Arteriolar Caliber ; ODR, Optic Disc Roundness; MRW, Minimum Rim Width; A multivariable logistic regression model was constructed including all variables with P < 0.10 in univariate analysis. Results are presented as adjusted odds ratios (OR) with 95% confidence intervals (CI). P < 0.05 was considered significant.

Supplemental Table 24. Multivariable logistic regression analysis of short-term SGLT2 inhibitor use in the PSM cohort.

| Group | Variable | B | SE | Wald | *P* | OR | 95％*CI* |
| --- | --- | --- | --- | --- | --- | --- | --- |
| HTN and T2DM  VS  CHD with HTN and T2DM | Intercept | 73.444 | 16.830 | 19.044 | 0.000 | >100 |  |
|  | Sex |  |  |  |  |  |  |
|  | Male | -4.650 | 1.441 | 10.415 | 0.001 | 0.010 | 0.001-0.1 |
|  | Female | Ref. |  |  |  |  |  |
|  | CCBs | 3.077 | 1.022 | 9.066 | 0.003 | 21.686 | 2.927-160.685 |
|  | MRAT | -15025.094 | 4375.853 | 11.790 | 0.001 | 0.000 | 0.000 |
|  | MRAC(Curvature)<sub>1.5-2.0PD</sub> | 4937.796 | 2346.398 | 4.429 | 0.035 | - | >100 |
|  | MRV(Venular)C | -0.914 | 0.207 | 19.551 | <0.001 | 0.401 | 0.267-0.601 |
|  | MRV(Venular)C<sub>2.0-2.5PD</sub> | 0.138 | 0.049 | 8.048 | 0.005 | 1.148 | 1.043-1.262 |

MRAT, Mean Retinal Arteriolar Tortuosity; MRAC(Curvature)<sub>1.5-2.0PD</sub>, Mean Retinal Arteriolar Curvature within 1.5-2.0 PD Zone; MRV(Venular)C, Mean Retinal Venular Caliber; MRV(Venular)C<sub>2.0-2.5PD</sub>, Mean Retinal Venular Caliber within 2.0-2.5 PD Zone; A multivariable logistic regression model was constructed including all variables with P < 0.10 in univariate analysis. Results are presented as adjusted odds ratios (OR) with 95% confidence intervals (CI). P < 0.05 was considered significant.
